# Characterization of *Ferredoxin-Dependent Glutamine-Oxoglutarate Amidotransferase (Fd-GOGAT)* Genes and Their Relationship with Grain Protein Content QTL in Wheat

**DOI:** 10.1371/journal.pone.0103869

**Published:** 2014-08-06

**Authors:** Domenica Nigro, Antonio Blanco, Olin D. Anderson, Agata Gadaleta

**Affiliations:** 1 Department of Soil, Plant and Food Sciences, Section of Genetic and Plant Breeding, University of Bari “Aldo Moro”, Bari, Italy; 2 Genomics and Gene Discovery Research Unit, Western Regional Research Center, USDA-ARS, Albany, California, United States of America; Nanjing Forestry University, China

## Abstract

**Background:**

In higher plants, inorganic nitrogen is assimilated via the glutamate synthase cycle or GS-GOGAT pathway. GOGAT enzyme occurs in two distinct forms that use NADH (NADH-GOGAT) or Fd (Fd-GOGAT) as electron carriers. The goal of the present study was to characterize wheat *Fd-GOGAT* genes and to assess the linkage with grain protein content (GPC), an important quantitative trait controlled by multiple genes.

**Results:**

We report the complete genomic sequences of the three homoeologous A, B and D Fd-GOGAT genes from hexaploid wheat (*Triticum aestivum*) and their localization and characterization. The gene is comprised of 33 exons and 32 introns for all the three homoeologues genes. The three genes show the same exon/intron number and size, with the only exception of a series of indels in intronic regions. The partial sequence of the Fd-GOGAT gene located on A genome was determined in two durum wheat (*Triticum turgidum* ssp. *durum*) cvs Ciccio and Svevo, characterized by different grain protein content. Genomic differences allowed the gene mapping in the centromeric region of chromosome 2A. QTL analysis was conducted in the Svevo×Ciccio RIL mapping population, previously evaluated in 5 different environments. The study co-localized the *Fd-GOGAT-A* gene with the marker GWM-339, identifying a significant major QTL for GPC.

**Conclusions:**

The wheat Fd-GOGAT genes are highly conserved; both among the three homoeologous hexaploid wheat genes and in comparison with other plants. In durum wheat, an association was shown between the *Fd-GOGAT* allele of cv Svevo with increasing GPC - potentially useful in breeding programs.

## Introduction

Glutamate is a central molecule in amino acid metabolism in higher plants. The α-amino group of glutamate is directly involved in both the assimilation and dissimilation of ammonia and is transferred to all other amino acids. In addition, both the carbon skeleton and α-amino group form the basis for the synthesis of γ-aminobutyric acid (GABA), arginine, and proline. Glutamate is also the precursor for chlorophyll synthesis in developing leaves [1].

As reviewed by Forde and Lea [2], glutamate synthase (GOGAT) is the key enzyme involved in the *de novo* synthesis of glutamate. It catalyzes the transfer of the amide group of glutamine to 2-oxoglutarate, with the result of two molecules of glutamate yielded.

The history of the discovery of the two enzymes, their structure, and gene regulation has been well documented [3], [4]. In plants, GOGAT enzyme occurs in two forms, depending on the electron donor involved in the reaction: it exist as a ferredoxin (Fd) dependent (EC 1.4.7.1), and a NADH dependent (EC 1.4.1.14) form.

Both forms are located in plastids, but, while Fd-dependent enzyme is usually present in high activities in the chloroplasts of photosynthetic tissues, NADH-dependent enzyme is predominantly located in non-photosynthesizing cells. The role of GOGAT enzymes have been well discussed in rice and conifers [5], [6]. Several studies showed that GOGAT mutations or gene knockouts, with a consequent reduced enzyme activity for both forms, seems to be involved in changes in amino acid metabolism [7], [8], [9], [10]. Only a few studies have reported plant gene isolation and sequencing, probably due togene lengths and structural complexity. For these reasons, the first reported studies on gene sequences described the isolation and sequencing of a full-length cDNA clone for maize *Fd-GOGAT* [11] and a partial cDNA clone for tobacco *Fd-GOGAT* [12]. The first plant complete genomic sequences identified were two genes from *Arabidopsis* [13]. For the *Triticeae* crops (wheat, barley, rye), no complete sequence has been known although partial sequences were reported for barley [14] and fragments for wheat [15].

Recently, NADH-GOGAT genomic sequences has been reported for the A and B genomes of tetraploid durum wheat (*Triticum turgidum ssp. durum)* and for the A, B, and D genomes of hexaploid wheat (*Triticum aestivum*) [16]. Analysis of the gene sequences indicates that all wheat *NADH-GOGAT* genes are composed of 22 exons and 21 introns. A comparative analysis of sequences among di- and mono-cotyledons plants shows both regions of high conservation and of divergence. qRT-PCR performed with the two durum wheat cvs Svevo and Ciccio (characterized by an high and low protein content, respectively) indicates different expression levels of the two *NADH-GOGAT-3A* and *NADH-GOGAT-3B* genes.

The Fd-GOGAT protein is a monomeric enzyme of 140–160 kDa and has been purified from barley leaves as a single polypeptide chain containing iron-sulfur and flavin. [17]. Fd-GOGAT activity has been mapped to the centromeric region of chromosome 2A [18] where we have previously reported a QTL for grain protein content (GPC) [19], [20].

GPC partially determines the nutritional value and the baking properties of common wheat (*T. aestivum*) as well as the pasta-making technology characteristics of durum wheat (*T. turgidum* ssp. *durum*). GPC is a typical quantitative trait controlled by a complex genetic system and influenced by environmental factors and management practices (nitrogen and water availability, temperature and light intensity). Breeding for high grain protein concentration has been one of the main goals for wheat breeders for several decades. Simultaneous increases of GPC and grain yield has been difficult to achieve because of a negative correlation between grain yield and GPC [21], [22]. This occurs because grain yield is mostly a consequence of starch accumulation, whereas protein accounts for less than 10–15% of the grain dry weight, and any increase in starch accumulation causes a dilution of the protein content if it is not accompanied by an equivalent increase in N accumulation. So far, any environmental factor that affects grain yield also affects GPC.

Recent investigations [23], [24], [25], [26], [27], [28], [29], [30], [31], [32], [20] indicated that factors influencing protein concentration in cultivated and wild wheats are located on all chromosomes. Heritability estimates for GPC ranged from 0.41 [33] to 0.70 [31], depending on the genetic material, environment and the computational methods. For this reason, the study of this important character through traditional methods is difficult and time consuming.

In the present work, we determined the DNA sequence of the three homoeologous *Fd-GOGAT* genes in hexaploid wheat, analyzed the exon/intron structure, compared the wheat sequences to other plants, and studied tetraploid durum grain protein content by QTL analysis and identification of candidate genes. In particular, we focused our attention on 2A chromosome where the *Fd*-*GOGAT* gene is located - identifying and characterizing the genomic sequence in durum wheat and determining its correlation with QTL for grain protein content (GPC).

## Results and Discussion

### Determination of genomic *Fd-glutamate synthase* (GOGAT) gene sequences

The complete sequences of A, B and D *Fd*-*GOGAT* genes of hexaploid wheat were obtained by assembling 454 sequences of cv Chinese Spring using a partial barley sequence (NCBI accession S58774; Gene ID: 548298) as the initial query. The Chinese Spring 454 assembly produced one contig comprised of the three A, B and D-genome sequences. Three independent contigs were then obtained by splitting the three sequences and assigned to A, B and D genomes by amplifying genome specific primers [15] on a set of nulli-tetrasomic lines (NTs) for chromosome group 2 of *Triticum aestivum* cv Chinese Spring [34], [35]. Examples of the analysis are shown for the A and B genomes in Figure S1 in File S1. The three homoeologous sequences are given in Text S1 in File S1.

**Figure 1:**
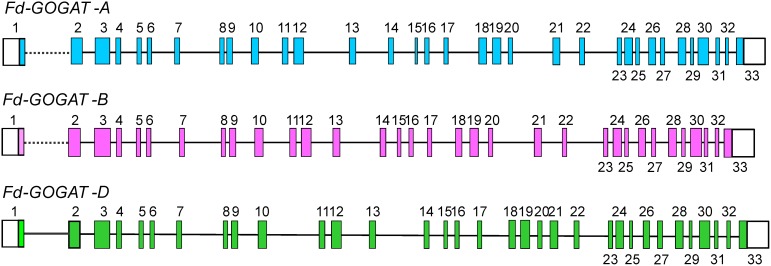
Diagrammatic representation of the structure of *Fd-GOGAT* genes. The three homoeologous Fd-GOGAT genes of wheat cv Chinese Spring are shown. Exons are numbered boxes. Coding sequences are colored, non-coding sequences are uncoloured boxes. Introns are intervening lines. Intron 1 is indicated by the dotted line.

Analysis of wheat gene sequences indicates that the wheat *Fd-GOGAT* gene is comprised of 33 exons and 32 introns for all the three homoeologous (Figure 1; Figure S2 in File S1). The sequence for intron 1 and the part of exon 1 containing the 5-coding portion of the exon could only be assigned for the D-genome gene (Figure 1; Text S1 and S2 in File S1). The cause of missing these sequences for the A- and B-genome genes is not known, but among possible causes are randomness of the shotgun sequencing, difficulty in next generation sequencing through the number of homopolymers in this region, or that the sequence is so similar in all three homoeologous that the software and manual assemblies mis-assigned some sequence reads to the D-genome sequence. For the A- and B-genome *Fd-GOGAT* genes, the 5′ extension ended with the final 13 amino acids of the signal peptide (Figure S2 in File S1). The 5′-UTR and the remainder of the coding sequence for the A-genome was found in the Transcriptome Shotgun Assembly (TRA) data at Genbank (Figure S3 in File S1).

The exon/intron borders for all three homoeologous wheat *Fd-GOGAT* genes matched the canonical plant borders (GT…AG) for all 32 identified introns (Figure 1). Consensus exon/introns boundaries were determined using grass EST sequences aligned to the genomic sequence and TRA assemblies. The structure of the *Fd-GOGAT* genes is highly conserved among the three homoeologues. In addition to the same number of exons/introns, no differences were observed in exons size and all three encoded mature enzymes have identical sequence lengths of 1519 amino acids residues (Figure S4 in File S1, Text S2 in File S1). Differences were observed in the intron sequences - in particular differences in length of 1–50 bases in introns 3–5, 8, 14–16, 22–23, 25–26, 28–31; 50–100 bp in introns 6, 12, 13, 21; and more than 500 bases in introns 9, 11, and 19 (Figure S2 in File S1). The three genes vary in length only by intron differences: the *Fd-GOGAT-A* gene is the longest with 15,337 bp, the *Fd-GOGAT-D* is 15,176 bp long and the gene located on chromosome 2B is the shortest at14,750 bp (all determined from the beginning of the sequence encoding the mature protein through the stop codon). The sequences among the three homoeologues are highly conserved – with only seven amino acid residue differences among the three mature polypeptides (Figure S4 in File S1).

### Comparison of Fd-GOGAT genes in other species

**Figure 2:**
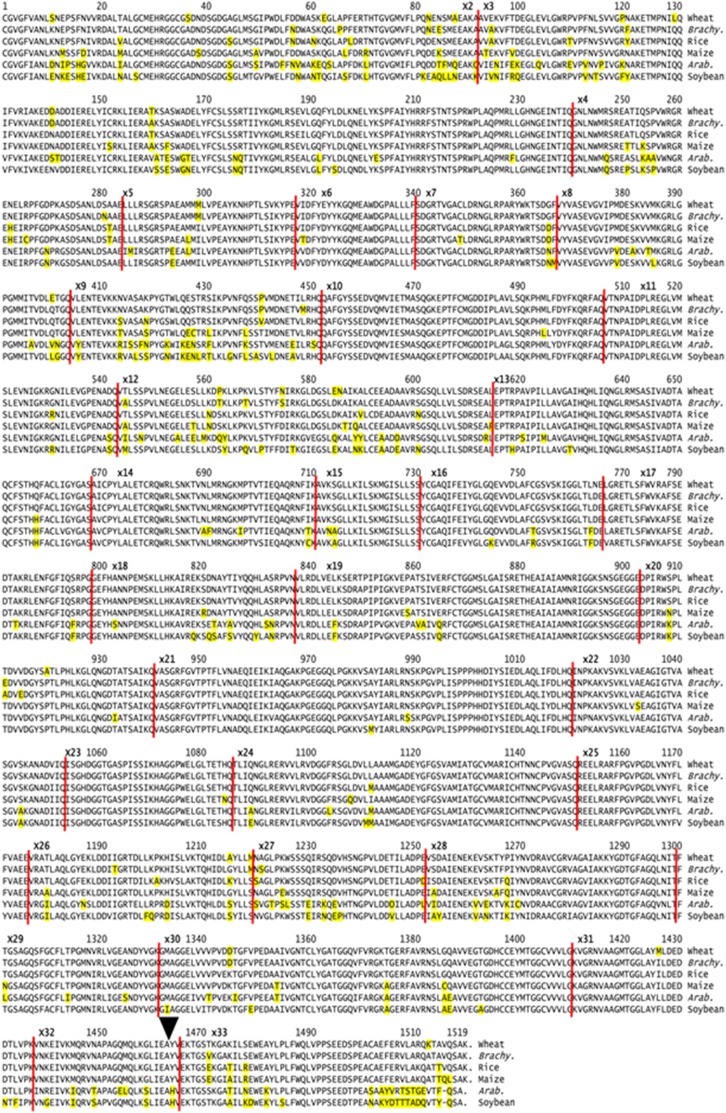
Plant Fd-GOGAT amino acid sequences. Four monocot and two dicot Fd-GOGAT amino acid sequences for the mature protein are aligned with Clustal V: Wheat (A genome; present report), *Brachypodium* (BRADI1G19080), Rice (Os07g46460), Maize (NM_001112223), *Arabidopsis* (CP002688), Soybean. (AK245357).

Conservation of the Fd-GOGAT sequences was further examined by comparing the A-genome wheat Fd-GOGAT mature amino acid sequence with a selection of monocot and dicot sequences available in public databases (Figure 2). The closest match was found with *Brachypodium* Fd-GOGAT (97%), with rice at 94% and maize at 92%. As expected, the dicot sequences were more divergent, at 82% for *Arabidopsis* and 84% for poplar. The monocots are all of the same length (1519 amino acids), and the dicot examples are of identical length through the first 1501 residues. The only length different, and the major sequence differences between the monocots and dicots is with the signal peptide (Figure S3 in File S1) and residues beyond 1501 (Figure 2).

**Figure 3:**
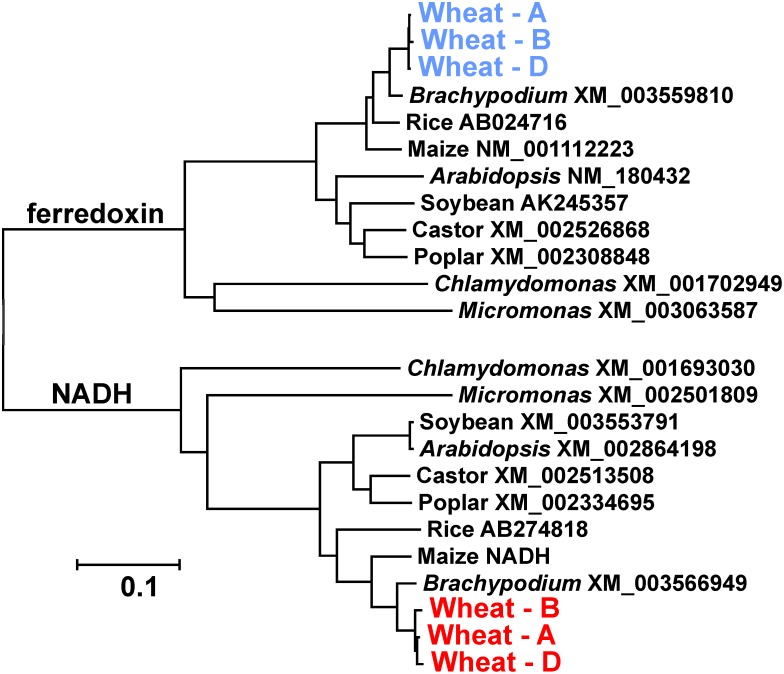
Phylogenetic tree of GOGAT polypeptides. The mature polypeptides for Fd- and NADH-GOGAT from a selection of plants an green algae were aligned with Clustal W and a nearest-neighbour tree generated with MEGA5. All three wheat homoeologues for Fd-GOGAT are in blue and red for the NADH-GOGAT version.

As noted above, there are two versions of GOGAT in plants – one requiring ferrodoxin and the other NADH. We have previously described the wheat *NADH-GOGAT* genes [16]. Figure 3 compares the amino acids sequences of the three wheat Fd-GOGAT homoeologous polypeptides with other monocots, dicots, and two green algae – the later being the most primitive organismal grouping yet found to contain both GOGAT versions. As expected, the two higher plant groups form two distinct branches for both GOGATs, with the green algae sequences distantly related within each of those two branches – consistent with the evolutionary distances. Less clarity is found when the analysis includes sequences from additional diverse groups; e.g., bacteria, cyanobacteria, Archeae, fungi, arthropods (Figure S5 in File S1).

### Glutamate synthase gene mapping

In order to find polymorphisms in the *Fd-GOGAT-A* gene, the “*CEL1* technology” was used in the durum wheat cvs Svevo and Ciccio - characterized by high and low grain protein content, respectively.

Allelic variations, SSRs, Indels, and SNPs are the major types of molecular markers that can be developed to detect DNA sequence. In particular, SNPs are nucleotide variations in the DNA sequence of individuals in a population and constitute the most abundant molecular markers in the genome. SNPs are widely distributed throughout genomes [36], vary in occurrence and distribution among species, and are usually more prevalent in the noncoding regions of the genome. There are several methods to discover SNPs; e.g., Sanger sequencing, DGCE (denaturing gradient capillary electrophoresis), denaturating HPLC and enzymatic mismatch cleavage. One of the most efficient and reliable enzymatic cleavage based method is TILLING (Targeting Induced Local Lesion In Genomes) [37], [38]. This approach requires a treatment of the amplified DNA with *CEL*I endonuclease, or any of a number of single strand endonucleases, after heteroduplex formation between the DNA of lines to be investigated. *CEL*I is a glycoprotein from celery, and many green plants. It cuts a DNA heteroduplex that contains a base-substitution or a DNA loop at the 3′ most phosphodiester bond of the mismatched nucleotides and produces two complementary sized fragments from the original amplified product (up to ∼1,500 bp amplicons). Electrophoresis size-separation on polyacrylamide or agarose gels is required to visualize any cleaved fragment.

**Figure 4:**
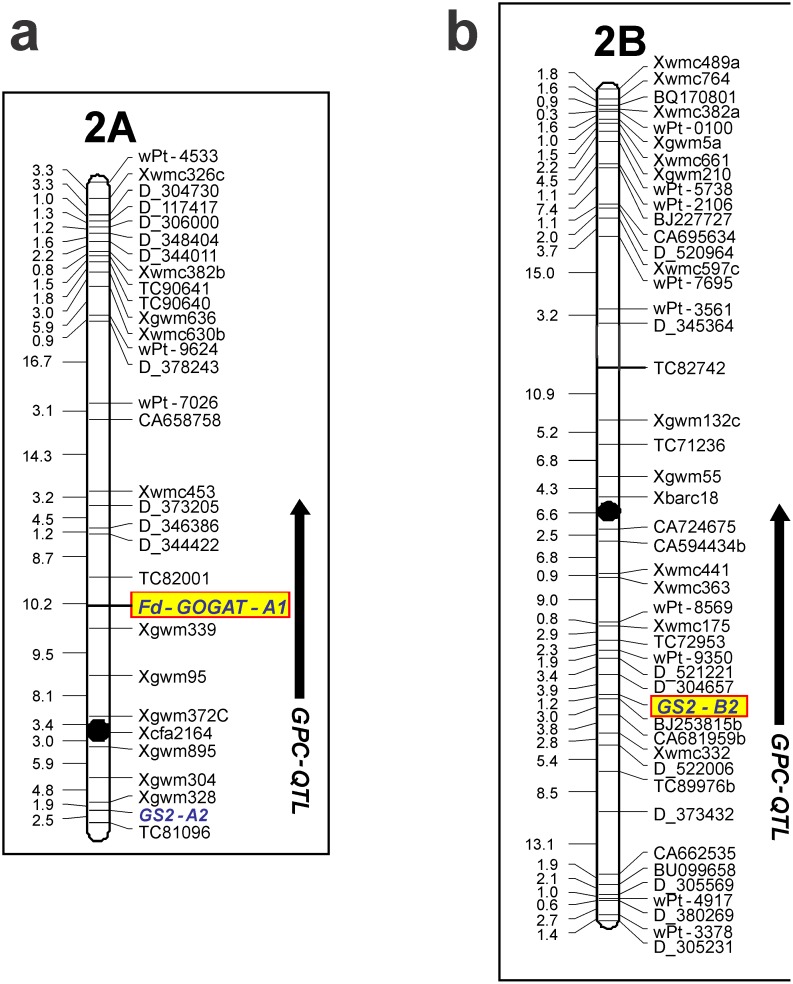
Genetic map position of Fd-GOGAT. The map positions and QTLs for grain protein content are shown in the side panels. *Fd-GOGAT-A* and *GS2-B2* loci, associated with GPC QTLs, respectively on chromosomes 2A and 2B, are highlighted in yellow. Black dots represent centromere. The genetic maps of wheat chromosomes 2A and 2B are from the Svevo×Ciccio RIL mapping population.

A set of primer pairs covering the entire *Fd-GOGAT-A* genomic sequence in (Table 1) was analyzed in the two parental lines Svevo and Ciccio in order to find SNPs. Out of 19 primer combinations amplified and digested with *CEL*1, four of them, (combinations 2, 4, 5 and 19) showed a digestion pattern in the heteroduplex lane but not in the single parental lines digestions, suggesting real differences exists between the two cvs (Figure 4). Mutations were confirmed by sequencing the fragments (see Materials and Method). A partial sequence of *Fd-GOGAT-A* gene was obtained for both cvs Ciccio and Svevo, which allowed finding a total of five SNPs and two indels between the two cultivars. Of the five SNPs detected, 2 were located in intronic regions (one each for intron 5 and intron 31) and 3 were located in exonic region (one for each exon 6, exon 31, and exon 32). Both indels were located in introns, a 2 bp indel in intron 29 and an 8 bp indel in intron 5.

**Table 1:** *Fd-GOGAT-A1* specific primer name, sequence and PCR condition used for SNPs detection.

Primer name	Forward sequence (5′-3′)	Reverse sequence (5′-3′)	PCR conditions
FdGOGAT/A_1	CGCCGTCGCTGTTGCCGC	TGCTGGCCCAATCATTAAACAAGT	69°C,
FdGOGAT/A_2	GCTTGTGGTGTTGGGTTTGTC	CTCTCTATCAGCTTTCGGCAG	60°C,
FdGOGAT/A_3	CGGTTCCTTTCAATCTATCAGT	AATCGGATGCTTTAGGGTCAC	58°C,
FdGOGAT/A_4_F	GGAAGCCACAATACAATCTC	CTAAACAAAAGTAAAGCAGG	54°C
DN_FdGOGAT/A_5	TCTATGAATACTACAAAGGT	CAACATAAACAAAACCATCT	54°C,
DN_FdGOGAT/A_6	TGACGGAAGGACGGTAGGGG	GAGGATTGGAAGTTGACAGGCT	63°C
DN_FdGOGAT/A_7	AAACCCTATGGAACTTGGCT	AAATCGCTGCTTGAAATAAT	65°C
DN_FdGOGAT/A_8	TCACAAGGGAAGGAGCCAACAT	TCAGAACGATCGGAAAGCAC	62°C
DN_FdGOGAT/A_9	TACCCTATCAAGTCCTGTCCTG	CAAACTGATGGGTGCTGAAA	58°C
DN_FdGOGAT/A_10	CACGGCCTGCTGTCCCAATAC	TGGTCACTGTGGGCATCTTG	68°C
DN_FdGOGAT/A_11	CATATCTGGCATTGGAAACAT	AGTTTTCCAGCCTCTTTGCG	65°C
DN_FdGOGAT/A_12	CTGGGTCGAGAAACACTATCA	TTGTTGGTAGATGGTGTATGC	58°C
DN_FdGOGAT/A_13	GTCAAAGCTGCTGCACAAAG	TTAATGGCACTTGTGGCGGT	60°C
DN_FdGOGAT/A_14	CAGATGTTGTTGATGGGTATT	CCCTTGTGCAATCTTTATCTCA	58°C
DN_FdGOGAT/A_15	TGCATCTGGACGTTTTGGTG	GCATTTGCCTTAGATACTCCAG	60°C
DN_FdGOGAT/A_16	GGTGTCGGTAAAGCTTGTAG	TGTGTTTCCGTAAGACCAAG	58°C
DN_FdGOGAT/A_17	CTCAATCAAGCATGCTGGGGG	CAAGATCAATGTGCTGCGTTT	62°C
DN_FdGOGAT/A_18	TACGAGCCACATTAGCCCAGT	CACATAATCGTTGGCCTCTC	60°C
DN_FdGOGAT/A_19	GGCAGTCCTTTGGTTGTTTTCT	TCTTCTTCGCTGGGTGGCA	62°C

Each PCR starts with 5 min at 94°C, followed by 35 cycles of 1 min denaturation at 94°C, 1 min annealing at the specific annealing T° reported above, and 2 min elongation at 72°C, and ends with a final elongation of 20 min at 72°C.

With the objective to genetically map the wheat *Fd-GOGAT-A* gene, a primer pair (Fd-GF, Fd-GR) was designed in one of the genomic region polymorphic between the cvs Ciccio and Svevo and analyzes in the RIL population. The linkage map “Svevo×Ciccio” developed by Gadaleta et al. [39] enriched of new DArT markers, were used for the gene mapping. The Fd-G marker produced, as expected, a single polymorphic fragment of 284 bp present in Svevo and lacking in Ciccio. The fragment was also physically mapped in the centromeric region of chromosome 2A on the bin C-2AS5-0.78 (data not shown). The linkage group identifying the 2A chromosome was of 129,9 cM including 31 markers (12 SSRs, 5 EST-SSRs, and 12DArT) and the *GS2-A2* and *Fd-GOGAT-A* genes (Figure 4A).

### Relationship between Fd-GOGAT-A and grain protein content

We focused our attention on the *Fd-GOGAT* gene located on chromosome 2A, where several authors found QTLs for GPC not linked to pleiotropic effects of low productivity in different genetic materials [18], [20], [40].

The RIL population Ciccio×Svevo was evaluated for grain protein content (GPC) and grain yield components in five field trials in southern Italy. The analysis of variance for GPC revealed highly significant differences between the parental lines Ciccio and Svevo and among the RIL in each of the field trials, suggesting that the RIL population was suitable for studying the putative involvement of the *GOGAT* genes in the control of grain protein content.

QTL analysis reported in Blanco et al. [20] detected ten QTLs for grain protein content on chromosome arms 1AS, 1AL, 2AS (two loci), 2BL, 3BS, 4AL, 4BL, 5AL and 6BS. In order to assess the putative relation between the *Fd-GOGAT-A* genes and GPC we re-analyzed the RIL data with the Inclusive Composite Interval Mapping method [41] in each of the five environments and across environments using the “Ciccio×Svevo” map data [39] enriched with new DArT marker and including the segregation data of the new Fd-GOGAT-A marker.

**Figure 5:**
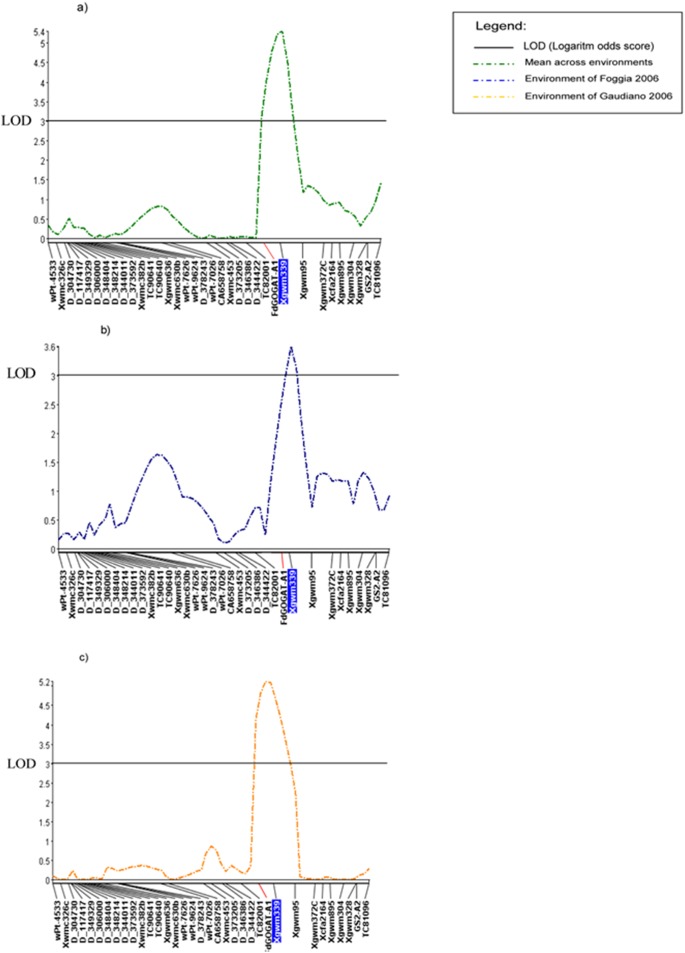
Grain protein QTLs. LOD score scan on chromosome 2A for QTLs associated with grain protein content. The significant scan for QTLs for each environment: A) mean across environments; B) Foggia 2006; C) Gaudiano 2006. The position and the name of molecular markers are shown on the chromosome along the horizontal axis. The LOD score scan was obtained by ICIM with highlights the markers used as cofactors. LOD stands for logarithm of the odds (to the base 10). A LOD score of three or more is generally considered significant - a LOD score of three means the odds are a thousand to one in favour of genetic linkage.

Among all the putative QTL for GPC in individual environments and across environments, only QTLs with LOD≥3.0 values were considered in the present work. This new QTL analysis revealed that the *Fd-GOGAT-A* gene mapped in the present work co-localized with a major QTL for GPC. In particular the *Fd-GOGAT-A* gene co-localised with a GPC-QTL detected on chromosome arm 2AS, in the region comprised between the markers *Xgwm372c* and the EST-SSR TC82001 (including the two closer markers *Xgwm339* and *Xgwm95)* significant in two environment and across environments. (Table 2, Figures 4 and 5. The Svevo allele increasing the trait had a positive additive effects ranging from 0.13 to 0.27 with a mean value of 0.24. R^2^ value, and the percentage of phenotypic variance explained by the additive effects of the mapped QTL, ranged from 6% to 19.4% between environments and the mean was 19.4 across environments.

**Table 2:** Additive gene effects of the detected QTL for grain protein content in the 2AS region (flanked by the markers TC82001 and *Xgwm372c* and including *FdGOGAT-A1*) in the Svevo×Ciccio RIL population grown at five environments.

Environment	Effect^a^	LOD	R^2^
Valenzano 2006	0,13	1,6	6,4
Foggia 2006	**0,27**	**4,7**	**17,4**
Gaudiano 2006	**0,27**	**3,5**	**13,3**
Valenzano2007	*0,18*	*2,8*	*10,7*
Foggia 2007	0,20	1,5	6,0
Across environments	**0,24**	**5,3**	**19,4**

a Effect: positive additive effects are associated with an increased effect from Svevo allele.

R^2^: Percentage of phenotypic variance explained by the additive effects of the mapped QTL.

## Conclusions

The current report describes the characterization of the bread wheat genomic sequence of *Fd-GOGAT* genes and their association with grain protein content (GPC). These gene sequences were useful to study the grain protein content in durum wheat, a quantitative trait controlled by multiple genes and influenced by environmental conditions.

The involvement of *Fd-GOGAT-A* gene in the control of GPC was carried out with three actions: isolation of homoeologous allele located on 2A chromosome in two elite durum wheat cultivars with different GPC, gene mapping in a segregant population, and association between the gene and GPC evaluated in five environments. In the present work, we were able to assemble the three complete homoeologous genes from the three hexaploid wheat genomes using as initial query a partial barley sequence to extract and assembly 454 reads from public databases. The three homoeologous genes have the same intron/exon structure with several differences in both intron and exon. We then used a set of aneuploid lines that led us to attribute PCR fragments to the A and B genomes.

In order to quickly screen the gene sequence looking for SNPs between our durum cultivars, we followed a “PCR/*CEL1* strategy” similar to a TILLING approach. In this approach, *CEL1* cleaves at the site of heteroduplex indicating mismatches in the sequences. This allowed us to identify only SNPs between our two cultivars avoiding the sequencing of the complete genes. A total of five SNPs and two indels were found of which an insertion of 8 bp in cv Svevo was used to construct specific markers and map the gene in a segregant population (Svevo×Ciccio). Mapping data of the polymorphic fragment allowed us to identify the locus named *Fd-GOGAT-A*, in the centromeric region of chromosome 2A. QTL analysis performed with CIM (Composite Interval Mapping) confirmed the presence of the marker in a major QTL for grain protein content. Several studies localized QTLs for GPC on chromosomes of group 2 [18], [42], [43], [44]. The QTL analysis carried out in the RIL population Ciccio×Svevo, previously evaluated for grain protein content in five environments, showed that *Fd-GOGAT-A* co-localized with QTLs for GPC on chromosome arms 2AS – the CIM analysis identified a major QTL with a stable effect in two environments and across environments. The genomic differences existing between the two cvs might modify the final predicted protein functionality or might have a key role in the gene regulation and gene expression, determining a different GPC. Further investigation are needed to prove this involvement through genetic transformation and/or sexual complementation.

The influence of the group 2 chromosomes on GPC control was reported in different genetic material have previously indicated the key role of these chromosomes play in the control of GPC. QTLs for GPC on 2A and 2B were firstly reported by Joppa and Cantrell [45] using durum wheat ssp. *dicoccoides* chromosome substitution lines. Prasad et al. [23] reported a protein content QTL on a distal region of the chromosome arm 2AS, while Blanco et al. [40] identified a GPC-QTL on 2AS near the centromere. More recently was found a stable QTL on 2A (*QGPC.usw-A2)* expressed in three environments and a QTL on 2B (*QGpc.usw-B3)* significant in four of the six environments analyzed [31].

The coding sequences of the homoeologous wheat *Fd-GOGAT* genes showed higher conservation among the three than the *NADH-GOGAT* homoeologues previously described [16], with only seven amino acid differences among the three *Fd-GOGAT* homoeologues. The conservation extended in comparisons to other plant Fd-GOGAT amino acid sequences, with only the C-terminal region having major sequence divergence in monocots vs dicots. The two forms of GOGAT are clear when the analysis compares among plants and green algae (Figure 3).

The precise role and location of the two forms of GOGAT are not completely understood, but the NADH form seems involved in development and re-assimilation of ammonia with either the cytosol or amyloplasts [46], while the ferredoxin form in a key component in photosynthesis and nitrogen fixation within the chloroplasts [47]. Both plastid forms are believed to have originated from endosymbiotic cyanobacteria [48]. The phylogenetic tree of Figure S5 in File S1 initially suggests cyanobacteria (*Cyanobacterium*, *Cyanothece*, *Leptolyngbya*) may possess both forms, but closer examination of the sequences finds different accessions of each of those three species contains only a single gene which can cluster with either the Fd or NADH GOGAT forms (not shown). When combined with the anomalies in annotations and the complex branching in Figure S5 in File S1, a more detailed analysis of GOGAT genes in all phyla is needed to better understand GOGAT gene evolution.

The comparison of a set of plant *Fd-GOGAT* genes (wheat, *Brachypodium*, rice and maize) suggests regions of greater sequence and structure conservation likely related to critical enzymatic functions and metabolic control. The higher identity was observed between wheat and *Brachypodium* sequences, as expected due to the genetic closest between the two species.

Although the two forms of GOGAT, Fd and NADH, catalyze the same reaction, the gene structures and their roles in plant metabolism are not identical. The two forms have detectable conservation in amino acid sequence up to the point where the NADH-GOGAT form encodes a pyridine nucleotide-disulfide oxidoreductase domain at the C-terminus (arrowhead in Figure 2; dot blot in Figure S6 in File S1) – indicating additional metabolic aspect of the *NADH-GOGAT gene*.

In higher plants, ammonium, whether resulting from nitrate assimilation or from secondary sources, is first incorporated into glutamine in a reaction catalysed by glutamine synthetase, and then glutamate synthase (glutamine:oxoglutarate amidotransferase; GOGAT) catalyses the combination of glutamine with 2:oxoglutarate to form two molecules of glutamate, one of which serves as substrate for GS, while the other one is available for transport, storage or further metabolism. These two reactions form a cycle referred to as the GS/GOGAT pathway [49]. Tissues and subcellular localization of both *GS* and *GOGAT* genes, as well as their different expression level during plant growth, resulting in different enzymatic activity along all phenological stages, determine a specific synergy between the two genes [47]. In particular cytosolic GS1 is involved in the pathway with NADH-GOGAT, while plastidic GS2 works preferentially with Fd-GOGAT. This seems to be confirmed in our genetic material, where both genes *Fd-GOGAT-A* and *GS2* were associated with QTLs for GPC. In fact, a *GS2* gene on chromosome 2B (*GS2-B2)* was mapped and found to be involved in GPC control (Figure 4b) [19]. So, from these studies, it’s suggested that *Fd-GOGAT-A* gene works in synergy with *GS2-B2* gene. This hypothesis could be confirmed by further analysis such as gene expression analysis.

The present work determined the DNA sequences of the three Fd-GOGAT genes in hexaploid wheat and identified an allele for the increment of GPC in durum wheat and open the way to further investigation using the forward and reverse genetic approaches that have been successfully used to validate the role of *Fd-GOGAT-A* genes for grain production both in rice and maize [43].

## Materials and Methods

### Plant material

Two durum wheat cultivars (Ciccio and Svevo) were used to investigate the relation of *Fd-GOGAT* genomic sequences to GPC. The durum cultivars are parents of a mapping population represented by a set of 120 recombinant inbred lines (RILs) [39]. The two parents were chosen for differences in important qualitative and quantitative traits; i.e., grain yield components, grain protein content, yellow pigment, and adaptive traits.

Genomic DNA was isolated from fresh leaves using the method described by Sharp et al. [50] and subsequently purified by phenol-chloroform extraction. DNA amplifications were carried out as described by Gadaleta et al. [39].

Nulli-tetrasomic lines (NTs) of *Triticum aestivum* cv Chinese Spring [34], [35] were used to physically localize *Fd-GOGAT* markers to chromosomes. Chinese Spring di-telosomic lines [51] were used for the assignment of markers to each chromosomal arm. Physical location on chromosome bins of each PCR fragment was obtained using a set of wheat deletions lines dividing the A and B genome chromosomes in bins (kindly provided by B. S. Gill, USDA-ARS, Kansas State University) [52].

### Genes in cv Chinese spring

To isolate the complete sequences of *Fd*-*GOGAT* genes in bread wheat, we used the cDNA sequence of a partial barley Fd-GOGAT mRNA (Gene S58774) [14]. This sequence was used as the initial query probe to extract matching 454 genomic sequences of cv Chinese Spring publicly available (http://www.cerealsdb.uk.net/CerealsDB/Documents/DOC_search_reads.php), which were then assembled using the SeqMan module of the Lasergene software (DNAStar, Inc.).

### Fd-GOGAT sequences in the Italian durum wheat cvs Ciccio and Svevo

By using Oligo Explorer and Primer3 (http://frodo.wi.mit.edu/primer3/) software, a set of genome specific primer pairs were designed for the distinct *Fd-GOGAT* hexaploid sequences previously obtained: Chinese Spring 2A and 2B *Fd-GOGAT*. A-genome specific primer pairs were used to amplify target DNA from both Ciccio and Svevo parental lines using PCR condition reported in Table 1.

Single PCR fragments were directly purified with EuroGold Cycle Pure Kit and sequenced. Multiple PCR products were first cloned into the pCR4-TOPO vector (Invitrogen, Cloning Kit) following the manufacturer’s instructions, and then each fragment was sequenced (http://www.bmr-genomics.it/).

Sequences assembly were carried out using CodonCode Aligner and Geneious software. Gaps and uncertain sequence were resolved by primer walking. Regions of less coverage or ambiguous reads were rechecked with primers designed to cover those regions.

### Digestion with CEL1 and revelation fragments

In order to discover and map unknown mutations between the genomic sequences of the two varieties Ciccio and Svevo, Single Nucleotide Polymorphisms (SNPs) were detected using the Surveyor nuclease kit (Transgenomic, Inc.), following manufacture’s instruction. This approach requires a treatment of the amplified DNA with *CELI* endonuclease, or any of a number of single strand endonucleases, after heteroduplex formation between the lines to be investigated. Surveyor nuclease cleaves with high specificity at the 3′ side of any mismatch site in both DNA strands, including all base substitutions and insertion/deletions up to at least 12 nucleotides.

### Heteroduplex formation, CEL I digestion and gel analysis

An aliquot of each PCR product was used for hybridization to form heteroduplexes between the parental lines, following thermal cycler program: 95°C for 2 min; loop 1 for 8 cycles (94°C for 20 s, 73°C for 30 s, reduce temperature 1°C per cycle, ramp to 72°C at 0.5°C/s, 72°C for 1 min); loop 2 for 45 cycles (94°C for 20 s, 65°C for 30 s, ramp to 72°C at 0.5°C/s, 72°C for 1 min); 72°C for 5 min; 99°C for 10 min; loop 3 for 70 cycles (70°C for 20 s, reduce temperature 0.3°C per cycle); hold at 8°C. After annealing, DNA has been treated with Surveyor nuclease to cleave heteroduplexes by adding 0.2 µl of enzyme and 1.3 µl of Buffer 1X. The digestion step was done at 5°C for 90 minutes and stopped immediately by adding 5 µl of 0.225 EDTA and 2 µl of bromophenol blue loading dye per sample and mixing thoroughly.

PCR fragments of over 1000 bp were analyzed on 3% polyacrylamide gels. The 3% polyacrylamide gel was made with: 15 ml 5×TBE, 120 l dH2O, 11 ml 40% bis-acrylamide, 110 µl TEMED and 1 ml 10% APS. We used 100-lane vertical electrophoresis system (CBS Scientific, Del Mar, CA, USA). The images were analyzed manually on PowerPoint (Microsoft Corp., Seattle, WA, USA).

In order to confirm the polymorphisms within genome specific genes, the heteroduplex hybridization digestion pattern was compared to the ones obtained in each parental lines. Moreover, the PCR product giving a digestion pattern after *CEL1* treatment were re-amplified and sequenced (http://www.bmr-genomics.it/).

### Development of Fd-GOGAT specific markers, mapping and correlation with grain protein content

The *Fd-GOGAT* sequences of the two cvs Svevo and Ciccio were aligned using ClustalW from EBI website to identify polymorphisms. The marker Fd-G (Forward 5′-GCAAAACAACCAGGGCACATA-3′, Reverse 5′-TAGCTCCCTTCCCCAATACAT-3′) for *Fd-GOGAT-A,* was designed with Oligo Explorer software in the polymorphic regions. The polymorphic marker was mapped in the “Svevo×Ciccio” mapping population. The observed segregation ratio for the marker was tested by chi-square analysis for deviation from the expected 1∶1 ratio. The linkage analysis was performed by JoinMap v. 4.0 [53] and the Kosambi mapping function was used to calculate map distances [54].

Grain protein content (GPC) and yield components were evaluated in the RIL population “Svevo×Ciccio” in five different environments (Valenzano 2006, Gaudiano 2006, Foggia 2006, Valenzano 2007, Foggia 2007). QTL analysis was performed following the procedure indicated by Blanco et al. [20].

### DNA sequence analysis

DNA sequences were analyzed using the Seqman and Megalign modules of the Lasergene software (DNAstar, Inc.), and MEGA5 [55].

## Supporting Information

File S1 **Supporting figures and text. Figure S1. Chromosome mapping of the Fd-GOGAT genes on chromosomes 2A and 2B.** Genome specific markers were amplified from durum cultivars and hexaploid wheat cv Chinese Spring genetic stocks. A) The A-genome specific marker amplified in cvs Svevo, Ciccio, and Chinese Spring and sets of Chinese Spring nulli-tetrasomic deletion lines for chromosome group 2. The 350 bp fragment was absent in the nulli-2A-tetra-2B line, as indicated by arrows and confirming the localization on chromosome 2A. B) The B-genome specific marker amplified in cvs, Ciccio, Svevo, Chinese Spring and sets of nullitetrasomic deletion lines for chromosome group 2. The 450 bp fragment was absent in the nulli-2B-tetra-2A line, as indicated by arrows and confirming the localization on chromosome 2B. **Figure S2.** **Alignment of wheat FD-GOGAT genes.** Alignment of wheat cv Chinese Spring A, B, and D genome *Fd-GOGAT* genes from the beginning of exon 2 through the stop codons. Exons are indicated by red brackets and exon number above the alignments. The blue line indicates the signal peptide/mature polypeptide boundary. The stop codons are boxed in red. **Figure S3. Fd-GOGAT signal sequences.** The signal sequences encoded by six plant *Fd-GOGAT* genes are aligned with Clustal V: Wheat (A genome; present report and GAJL01283868), Brachypodium (BRADI1G19080), Rice (Os07g46460), Maize (NM_001112223), *Arabidopsis* (CP002688), Soybean. (AK245357). The red vertical line indicates the exon 1/exon 2 junction. The blue line indicates the end of the signal peptide. **Figure S4.** **Alignment of wheat Fd-GOGAT polypeptides.** Alignment of the three hexaploid wheat Fd-GOGAT polypeptides from the A, B, and D genomes. Differences in amino acid sequence are highlighted in yellow. **Figure S5. Phylogenetic tree of Fd- and NADH-GOGAT proteins from diverse species.** A selection of available GOGAT amino acid sequences from diverse phyla through genera were aligned with Clustal W and a phylogenetic tree formed by nearest-neighbor analysis. The wheat Fd-GOGAT homoeologues are in blue and red for the NADH-GOGAT version. Annotated sequences from other organisms have the same color coding as wheat. Unannotated sequences are in black. **Figure S6. Comparison of Fd-GOGAT and NADH-GOGAT amino acid sequences.** The amino acid sequences of the mature Fd-GOGAT from the A-genome is compared to the mature NADH-GOGAT from the A-genome by dot blot. Matching criteria was 80 match in a 5 amino acid residue window. **Text S1. Fasta file of the three cv Chinese Spring Fd-GOGAT genes. Text S2. Fasta file of the three cv Chinese Spring Fd-GOGAT mature proteins.**
(PDF)
