# Supplementary material for: Characterization of Ferredoxin-Dependent Glutamine-Oxoglutarate Amidotransferase (Fd-GOGAT) Genes and Their Relationship with Grain Protein Content QTL in Wheat
Source: PLoS One. 2014 Aug 6;9(8):e103869. doi: 10.1371/journal.pone.0103869 (PMC4123923; doi:10.1371/journal.pone.0103869)
Supplement: File S1 — Supporting figures and text. Figure S1. Chromosome mapping of the Fd-GOGAT genes on chromosomes 2A and 2B. Genome specific markers were amplified from durum cultivars and hexaploid wheat cv Chinese Spring genetic stocks. A) The A-genome specific marker amplified in cvs Svevo, Ciccio, and Chinese Spring and sets of Chinese Spring nulli-tetrasomic deletion lines for chromosome group 2. The 350 bp fragment was absent in the nulli-2A-tetra-2B line, as indicated by arrows and confirming the localization on chromosome 2A. B) The B-genome specific marker amplified in cvs, Ciccio, Svevo, Chinese Spring and sets of nullitetrasomic deletion lines for chromosome group 2. The 450 bp fragment was absent in the nulli-2B-tetra-2A line, as indicated by arrows and confirming the localization on chromosome 2B. Figure S2. Alignment of wheat FD-GOGAT genes. Alignment of wheat cv Chinese Spring A, B, and D genome Fd-GOGAT genes from the beginning of exon 2 through the stop codons. Exons are indicated by red brackets and exon number above the alignments. The blue line indicates the signal peptide/mature polypeptide boundary. The stop codons are boxed in red. Figure S3. Fd-GOGAT signal sequences. The signal sequences encoded by six plant Fd-GOGAT genes are aligned with Clustal V: Wheat (A genome; present report and GAJL01283868), Brachypodium (BRADI1G19080), Rice (Os07g46460), Maize (NM_001112223), Arabidopsis (CP002688), Soybean. (AK245357). The red vertical line indicates the exon 1/exon 2 junction. The blue line indicates the end of the signal peptide. Figure S4. Alignment of wheat Fd-GOGAT polypeptides. Alignment of the three hexaploid wheat Fd-GOGAT polypeptides from the A, B, and D genomes. Differences in amino acid sequence are highlighted in yellow. Figure S5. Phylogenetic tree of Fd- and NADH-GOGAT proteins from diverse species. A selection of available GOGAT amino acid sequences from diverse phyla through genera were aligned with Clustal W and a phylogenetic tree formed by neare [file pone.0103869.s001.pdf]

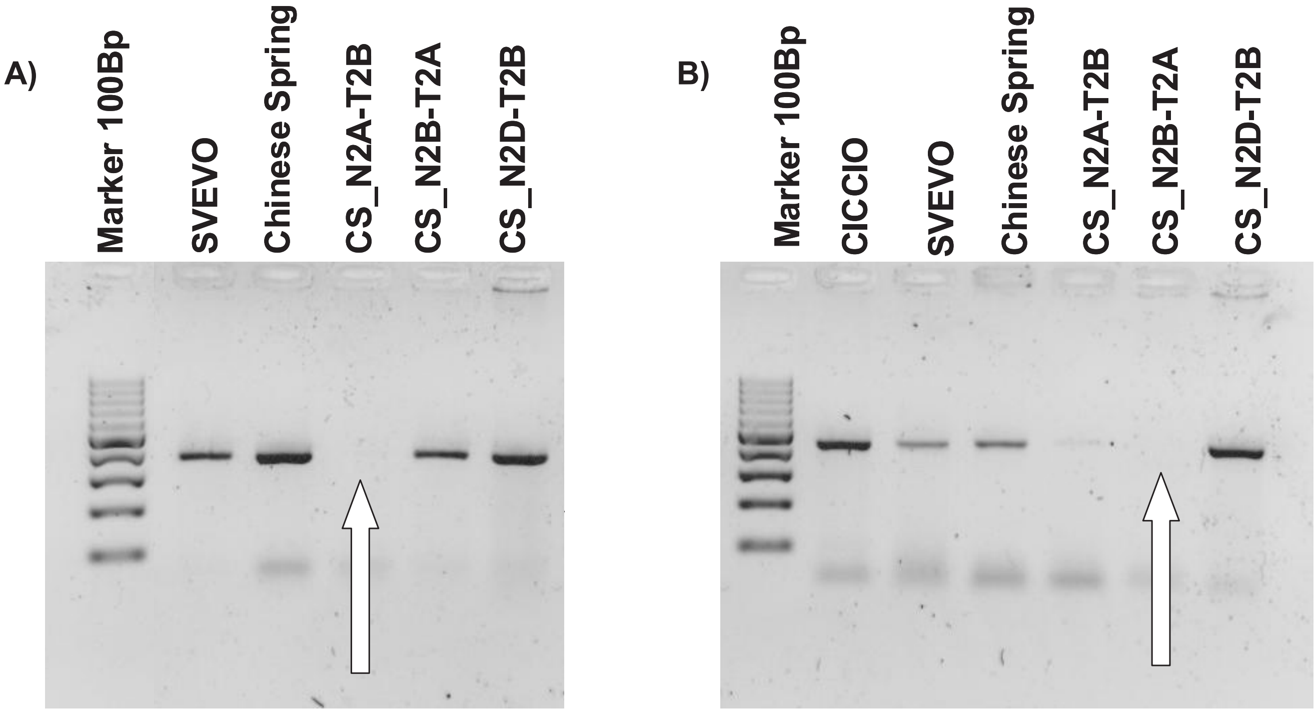
A)
Marker 100Bp
SVEVO
Chinese Spring
CS_N2A-T2B
CS_N2B-T2A
CS_N2D-T2B
B)
Marker 100Bp
CICCIO
SVEVO
Chinese Spring
CS_N2A-T2B
CS_N2B-T2A
CS_N2D-T2B

Figure S1.

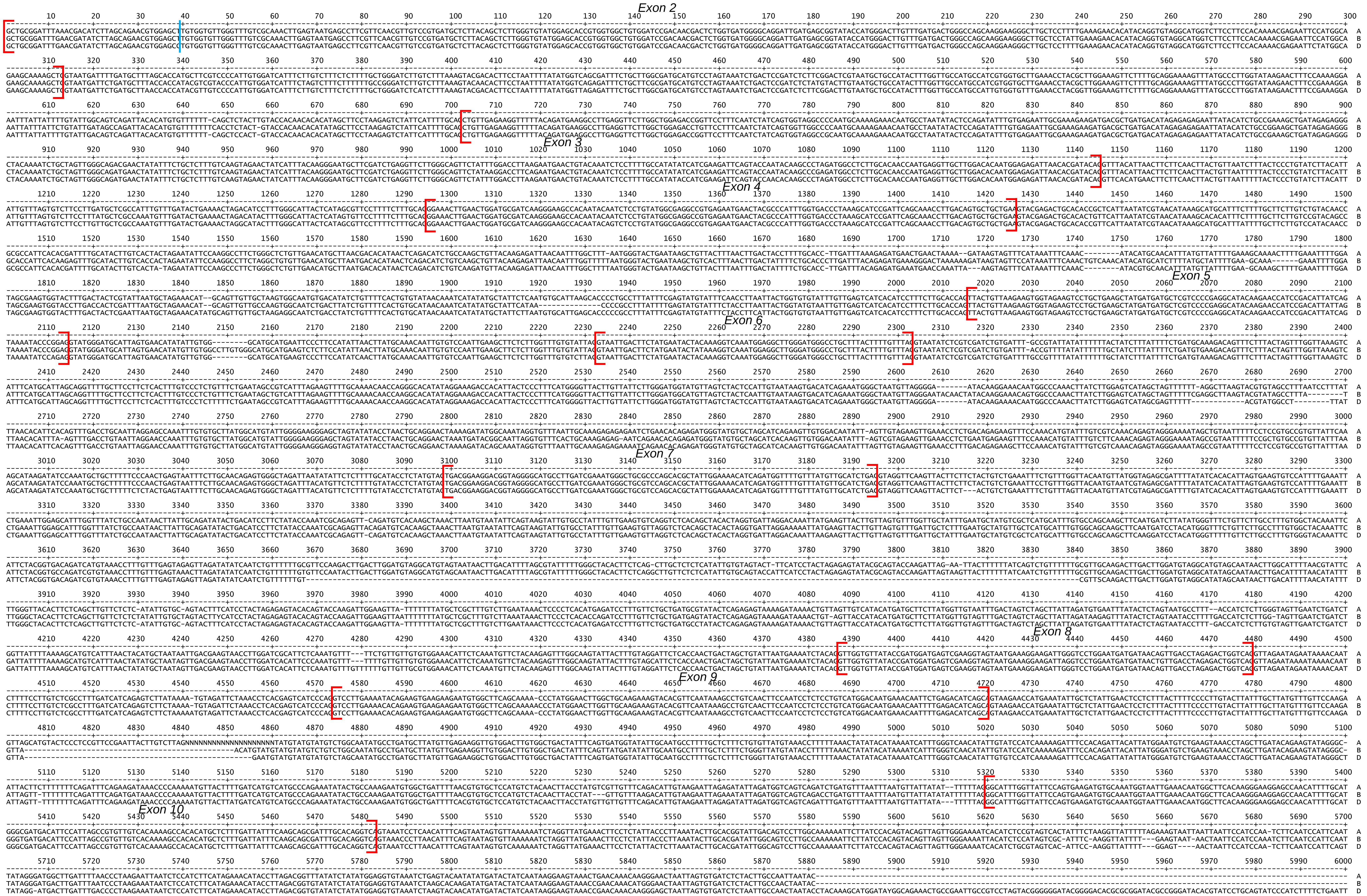
Exon 2
Exon 3
Exon 4
Exon 5
Exon 6
Exon 7
Exon 8
Exon 9
Exon 10

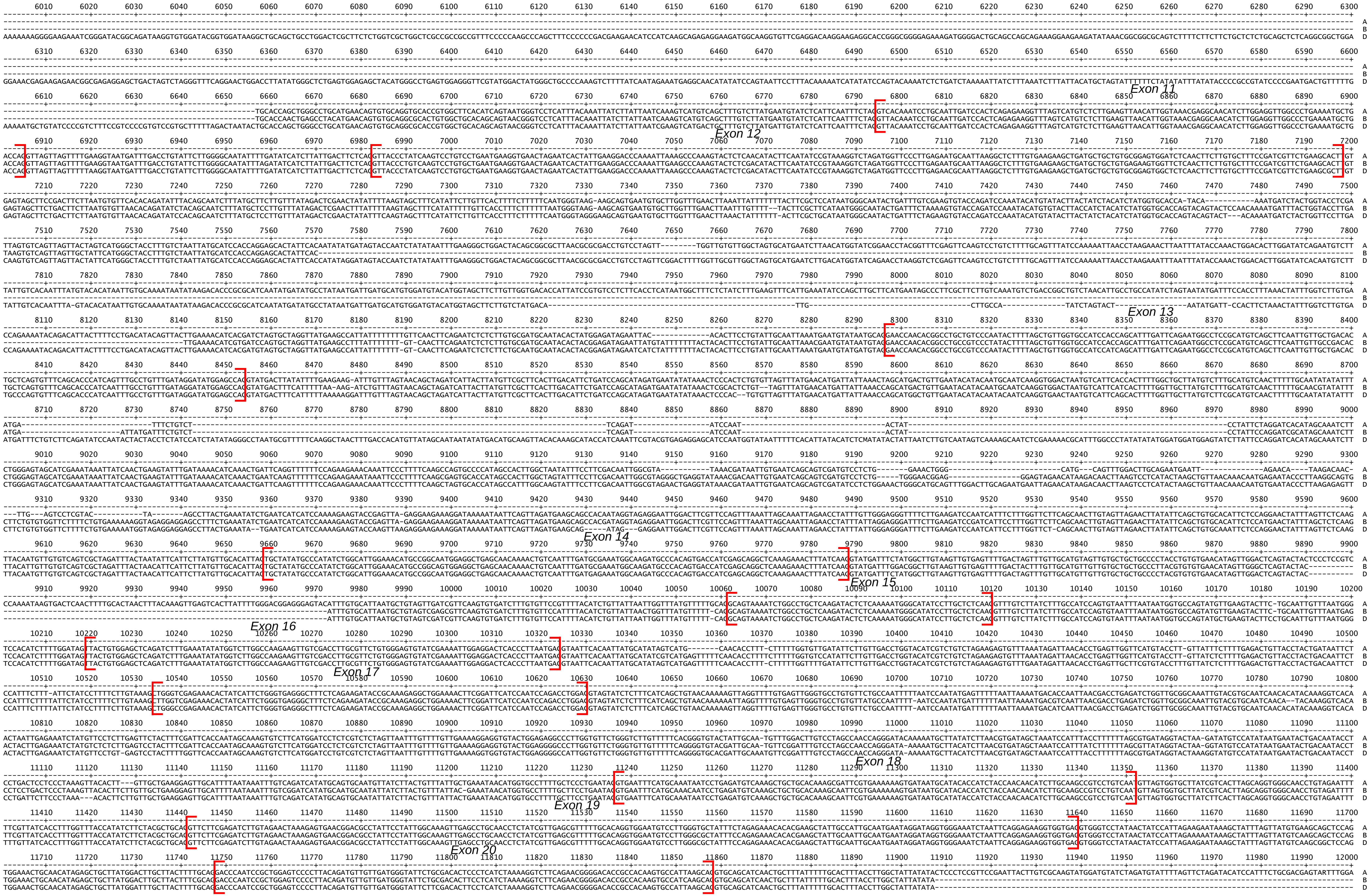

Exon 11
Exon 12
Exon 13
Exon 14
Exon 15
Exon 16
Exon 17
Exon 18
Exon 19
Exon 20

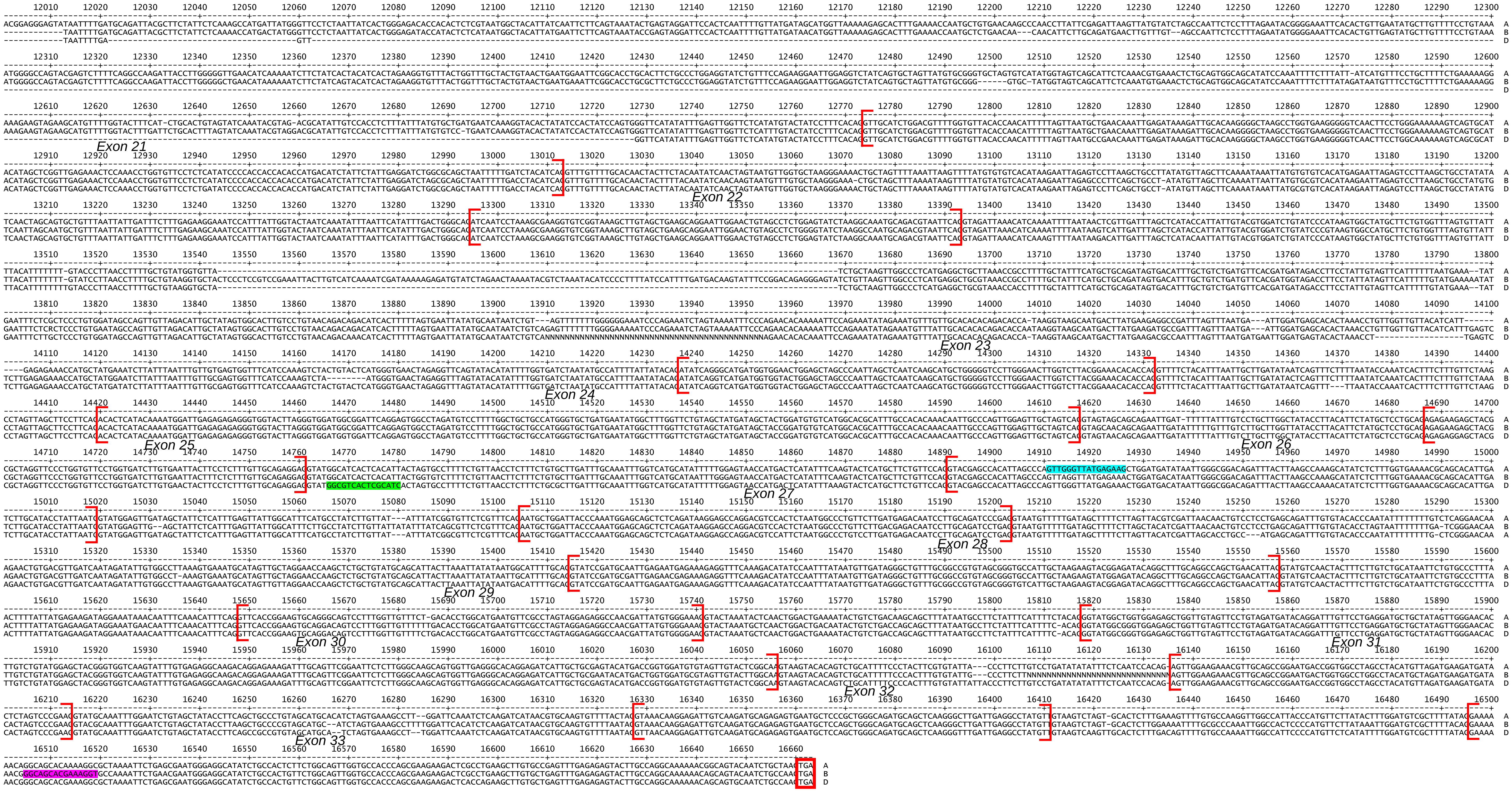
Exon 21
Exon 22
Exon 23
Exon 24
Exon 25
Exon 26
Exon 27
Exon 28
Exon 29
Exon 30
Exon 31
Exon 32
Exon 33

Figure S2

```
MAATLPRAVA-----PPPALLPLPRAAP-LLLAGRAAAR----RLRARGARTPALAATRRSWAV------SARAV  Wheat
MAATLPRAVA-----PPS-LLPLPRAAP-LLLAGRAAAR----RLRARGARAPALAAARRSWAV------STRAV  Brachy.
MA-TLPRAAAAAAPSPAAALLPLPRAAP--LLAGRAAARSAARRLRARGTRAPPLAAARRGW-----GGVSPRAV  Rice
MA-TLPRAA----PPTPAALLPLPRAAPPLLLAGRAAA-ARRSRLRARG----PSAAARRSWVVASAASSSSRAV  Maize
MA-MQS--LSPVPKLLSTTPSSVLSSDKNFFFVDFVGLYCKSKRTRRRLRGD---SSSSSRSSSSLSRLSSVRAV  Arab.
MA-LHS--VSSVSHVLRLA-EPFPSLHNAHVLLDLAPLRRKPKRRTRRLT-AFPLSSPLRH--------SAVKSV  Soybean

                                           Exon 1      Exon 2
LDLPRRR---------AP---QKPA-----------------------QEAADLNDILAERGACGVGFVAN...  Wheat
LDVPRHR---------APPPAQKPV-----------------------QEAADLNDILAERGACGVGFVAN...  Brachy.
LDLPRRRE--------AA---EKPA-----------------------QKAADLNEILSERGACGVGFVAN...  Rice
VGGVARRE--------APPAPQKPT-----------------------QQAADLNHILSERGACGVGFVAN...  Maize
IDLER--VHGVSEKDLSSPSALRPQVRFFTDINFTNTQRAKFHPLWGSFKQVANLEDILSERGACGVGFIAN...  Arab.
LHLDRSTDNRLHNSSASSSSDSKP-------------------------QVANLEDIISERGACGVGFIAN...  Soybean
```

Figure S3.

```
        10        20        30        40        50        60        70        80        90       100       110       120
---------+---------+---------+---------+---------+---------+---------+---------+---------+---------+---------+---------+
CGVGFVANLSNEPSFNVVRDALTALGCMEHRGGCGSDNDSGDGAGLMSGIPWDLFDDWASKEGLAPFERTHTGVGMVFLPQNENSMAEAKAAVEKVFTDEGLEVLGWRPVPFNLSVVGPN  A
CGVGFVANLSNEPSFNVVRDALTALGCMEHRGGCGSDNDSGDGAGLMSGIPWDLFDDWASKEGLAPIERTHTGVGMVFLPQNENSMAEAKAAVEKVFTDEGLEVLGWRPVPFNLSVVGPN  B
CGVGFVANLSNEPSFNVVRDALTALGCMEHRGGCGSDNDSGDGAGLMSGIPWDLFDDWASKEGLAPFERTHTGVGMVFLPQNENSMAEAKAAVEKVFTDEGLEVLGWRPVPFNLSVVGRN  D

       130       140       150       160       170       180       190       200       210       220       230       240
---------+---------+---------+---------+---------+---------+---------+---------+---------+---------+---------+---------+
AKETMPNILQIFVRIAKEDDADDIERELYICRKLIERATKSASWADELYFCSLSSRTIIYKGMLRSEVLGQFYLDLKNELYKSPFAIYHRRFSTNTSPRWPLAQPMRLLGHNGEINTIQG  A
AKETMPNILQIFVRIAKEDDADDIERELYICRKLIERATKSASWADELYFCSLSSRTIIYKGMLRSEVLGQFYKDLQNELYKSPFAIYHRRFSTNTSPRWPLAQPMRLLGHNGEINTIQG  B
AKETMPNILQIFVRIAKEDDADDIERELYICRKLIERATKSASWADELYFCSLSSRTIIYKGMLRSEVLGQFYLDLKNELYKSPFAIYHRRFSTNTSPRWPLAQPMRLLGHNGEINTIQG  D

       250       260       270       280       290       300       310       320       330       340       350       360
---------+---------+---------+---------+---------+---------+---------+---------+---------+---------+---------+---------+
NLNWMRSREATIQSPVWRGRENELRPFGDPKASDSANLDSAAELLLRSGRSPAEAMMMLVPEAYKNHPTLSVKYPEVIDFYEYYKGQMEAWDGPALLLFSDGRTVGACLDRNGLRPARYW  A
NLNWMRSREATIQSPVWRGRENELRPFGDPKASDSANLDSAAELLLRSGRSPAEAMMMLVPEAYKNHPTLLVKYPEVIDFYEYYKGQMEAWDGPALLLFSDGRTVGACLDRNGLRPARYW  B
NLNWMRSREATIQSPVWRGRENELRPFGDPKASDSANLDSAAELLLRSGRSPAEAMMMLVPEAYKNHPTLSVKYPEVIDFYEYYKGQMEAWDGPALLLFSDGRTVGACLDRNGLRPARYW  D

       370       380       390       400       410       420       430       440       450       460       470       480
---------+---------+---------+---------+---------+---------+---------+---------+---------+---------+---------+---------+
KTSDGFVYVASEVGVIPMDESKVVMKGRLGPGMMITVDLETGQVLENTEVKKNVASAKPYGTWLQESTRSIKPVNFQSSPVMDNETILRHQQAFGYSSEDVQMVIETMASQGKEPTFCMG  A
KTSDGFVYVASEVGVIPMDESKVVMKGRLGPGMMITVDLETGQVLENTEVKKNVASAKPYGTWLQESTRSIKPVNFQSSPVMDNETILRHQQAFGYSSEDVQMVIETMASQGKEPTFCMG  B
KTSDGFVYVASEVGVIPMDESKVVMKGRLGPGMMITVDLETGQVLENTEVKKNVASAKPYGTWLQESTRSIKPVNFQSSPVMDNETILRHQQAFGYSSEDVQMVIETMASQGKEPTFCMG  D

       490       500       510       520       530       540       550       560       570       580       590       600
---------+---------+---------+---------+---------+---------+---------+---------+---------+---------+---------+---------+
DDIPLAVLSQKPHMLFDYFKQRFAQVTNPAIDPLREGLVMSLEVNIGKRGNILEVGPENADQVTLSSPVLNEGELESLLKDPKLKPKVLSTYFNIRKGLDGSLENAIKALCEEADAAVRS  A
DDIPLAVLSQKPHMLFDYFKQRFAQVTNPAIDPLREGLVMSLEVNIGKRGNILEVGPENADQVTLSSPVLNEGELESLLKDPKLKPKVLSTYFNIRKGLDGSLENAIKALCEEADAAVRS  B
DDIPLAVLSQKPHMLFDYFKQRFAQVTNPAIDPLREGLVMSLEVNIGKRGNILEVGPENADQVTLSSPVLNEGELESLLKDPKLKPKVLSTYFNIRKGLDGSLENAIKALCEEADAAVRS  D

       610       620       630       640       650       660       670       680       690       700       710       720
---------+---------+---------+---------+---------+---------+---------+---------+---------+---------+---------+---------+
GSQLLVLSDRSEALEPTRPAVPILLAVGAIHQHLIQNGLRMSASIVADTAQCFSTHQFACLIGYGASAICPYLALETCRQWRLSNKTVNLMRNGKMPTVTIEQAQRNFIKAVKSGLLKIL  A
GSQLLVLSDRSEALEPTRPAVPILLAVGAIHQHLIQNGLRMSASIVADTAQCFSTHQFACLIGYGASAICPYLALETCRQWRLSNKTVNLMRNGKMPTVTIEQAQRNFIKAVKSGLLKIL  B
GSQLLVLSDRSEALEPTRPAVPILLAVGAIHQHLIQNGLRMSASIVADTAQCFSTHQFACLIGYGASAICPYLALETCRQWRLSNKTVNLMRNGKMPTVTIEQAQRNFIKAVKSGLLKIL  D

       730       740       750       760       770       780       790       800       810       820       830       840
---------+---------+---------+---------+---------+---------+---------+---------+---------+---------+---------+---------+
SKMGISLLSSYCGAQIFEIYGLGQEVVDLAFCGSVSKIGGLTLNELGRETLSFWVRAFSEDTAKRLENFGFIQSRPGGEFHANNPEMSKLLHKAIREKSDNAYTIYQQHLASRPVNVLRD  A
SKMGISLLSSYCGAQIFEIYGLGQEVVDLAFCGSVSKIGGLTLNELGRETLSFWVRAFSEDTAKRLENFGFIQSRPGGEFHANNPEMSKLLHKAIREKSDNAYTIYQQHLASRPVNVLRD  B
SKMGISLLSSYCGAQIFEIYGLGQEVVDLAFCGSVSKIGGLTLNELGRETLSFWVRAFSEDTAKRLENFGFIQSRPGGEFHANNPEMSKLLHKAIREKSDNAYTIYQQHLASRPVNVLRD  D

       850       860       870       880       890       900       910       920       930       940       950       960
---------+---------+---------+---------+---------+---------+---------+---------+---------+---------+---------+---------+
LVELKSERTPIPIGKVEPATSIVERFCTGGMSLGAISRETHEAIAIAMNRIGGKSNSGEGGEDPIRWSPLTDVVDGYSATLPHLKGLQNGDTATSAIKQVASGRFGVTPTFLVNAEQIEI  A
LVELKSERTPIPIGKVEPATSIVERFCTGGMSLGAISRETHEAIAIAMNRIGGKSNSGEGGEDPIRWSPLTDVVDGYSATLPHLKGLQNGDTATSAIKQVASGRFGVTPTFLVNAEQIEI  B
LVELKSERTPIPIGKVEPATSIVERFCTGGMSLGAISRETHEAIAIAMNRIGGKSNSGEGGEDPIRWSPLTDVVDGYSSTLPHLKGLQNGDTATSAIKQVASGRFGVTPTFLVNAEQIEI  D

       970       980       990      1000      1010      1020      1030      1040      1050      1060      1070      1080
---------+---------+---------+---------+---------+---------+---------+---------+---------+---------+---------+---------+
KIAQGAKPGEGGQLPGKKVSAYIARLRNSKPGVPLISPPPHHDIYSIEDLAQLIFDLHQINPKAKVSVKLVAEAGIGTVASGVSKANADVIQISGHDGGTGASPISSIKHAGGPWELGLT  A
KIAQGAKPGEGGQLPGKKVSAYIARLRNSKPGVPLISPPPHHDIYSIEDLAQLIFDLHQINPKAKVSVKLVAEAGIGTVASGVSKANADVIQISGHDGGTGASPISSIKHAGGPWELGLT  B
KIAQGAKPGEGGQLPGKKVSAYIARLRNSKPGVPLISPPPHHDIYSIEDLAQLIFDLHQINPKAKVSVKLVAEAGIGTVASGVSKANADVIQISGHDGGTGASPISSIKHAGGPWELGLT  D

      1090      1100      1110      1120      1130      1140      1150      1160      1170      1180      1190      1200
---------+---------+---------+---------+---------+---------+---------+---------+---------+---------+---------+---------+
ETHQTLIQNGLRERVVLRVDGGFRSGLDVLLAAAMGADEYGFGSVAMIATGCVMARICHTNNCPVGVASQREELRARFPGVPGDLVNYFLFVAEEVRATLAQLGYEKLDDIIGRTDLLKP  A
ETHQTLIQNGLRERVVLRVDGGFRSGLDVLLAAAMGADEYGFGSVAMIATGCVMARICHTNNCPVGVASQREELRARFPGVPGDLVNYFLFVAEEVRATLAQLGYEKLDDIIGRTDLLKP  B
ETHQTLIQNGLRERVVLRVDGGFRSGLDVLLAAAMGADEYGFGSVAMIATGCVMARICHTNNCPVGVASQREELRARFPGVPGDLVNYFLFVAEEVRATLAQLGYEKLDDIIGRTDLLKP  D

      1210      1220      1230      1240      1250      1260      1270      1280      1290      1300      1310      1320
---------+---------+---------+---------+---------+---------+---------+---------+---------+---------+---------+---------+
KHISLVKTQHIDLAYLLMNAGLPKWSSSQIRSQDVHSNGPVLDETILADPEVSDAIENEKEVSKTYPIYNVDRAVCGRVAGAIAKKYGDTGFAGQLNITFTGSAGQSFGCFLTPGMNVRL  A
KHISLVKTQHIDLAYLLMNAGLPKWSSSQIRSQDVHSNGPVLDETILADPEVSDAIENEKEVSKTYPIYNVDRAVCGRVAGAIAKKYGDTGFAGQLNITFTGSAGQSFGCFLTPGMNVRL  B
KHISLVKTQHIDLAYLLMNAGLPKWSSSQIRSQDVHSNGPVLDETILADPEVSDAIENEKEVSKTYPIYNVDRAVCGRVAGVIAKKYGDTGFAGQLNITFTGSAGQSFGCFLTPGMNVRL  D

      1330      1340      1350      1360      1370      1380      1390      1400      1410      1420      1430      1440
---------+---------+---------+---------+---------+---------+---------+---------+---------+---------+---------+---------+
VGEANDYVGKGMAGGELVVVPVDDTGFVPEDAAIVGNTCLYGATGGQVFVRGKTGERFAVRNSLGQAVVEGTGDHCCEYMTGGCVVVLGKVGRNVAAGMTGGLAYMLDEDDTLVPKVNKE  A
VGEANDYVGKGMAGGELVVVPVDDTGFVPEDAAIVGNTCLYGATGGQVFVRGKTGERFAVRNSLGQAVVEGTGDHCCEYMTGGCVVVLGKVGRNVAAGMTGGLAYMLDEDDTLVPKVNKE  B
VGEANDYVGKGMAGGELVVVPVDDTGFVPEDAAIVGNTCLYGATGGQVFVRGKTGERFAVRNSLGQAVVEGTGDHCCEYMTGGCVVVLGKVGRNVAAGMTGGLAYMLDEDDTLVPKVNKE  D

      1450      1460      1470      1480      1490      1500      1510      1520
---------+---------+---------+---------+---------+---------+---------+---------+
IVKMQRVNAPAGQMQLKGLIEAYVEKTGSTKGAKILSEWEAYLPLFWQLVPPSEEDSPEACAEFERVLARQKTAVQSAK.  A
IVKMQRVNAPAGQMQLKGLIEAYVEKTGSTKGAKILNEWEAYLPLFWQLVPPSEEDSPEACAEFERVLARQKTAVQSAK.  B
IVKMQRVNAPAGQMQLKGLIEAYVEKTGSTKGAKILSEWEAYLPLFWQLVPPSEEDSPEACAEFERVLARQKTAVQSAK.  D
```

Figure S4.

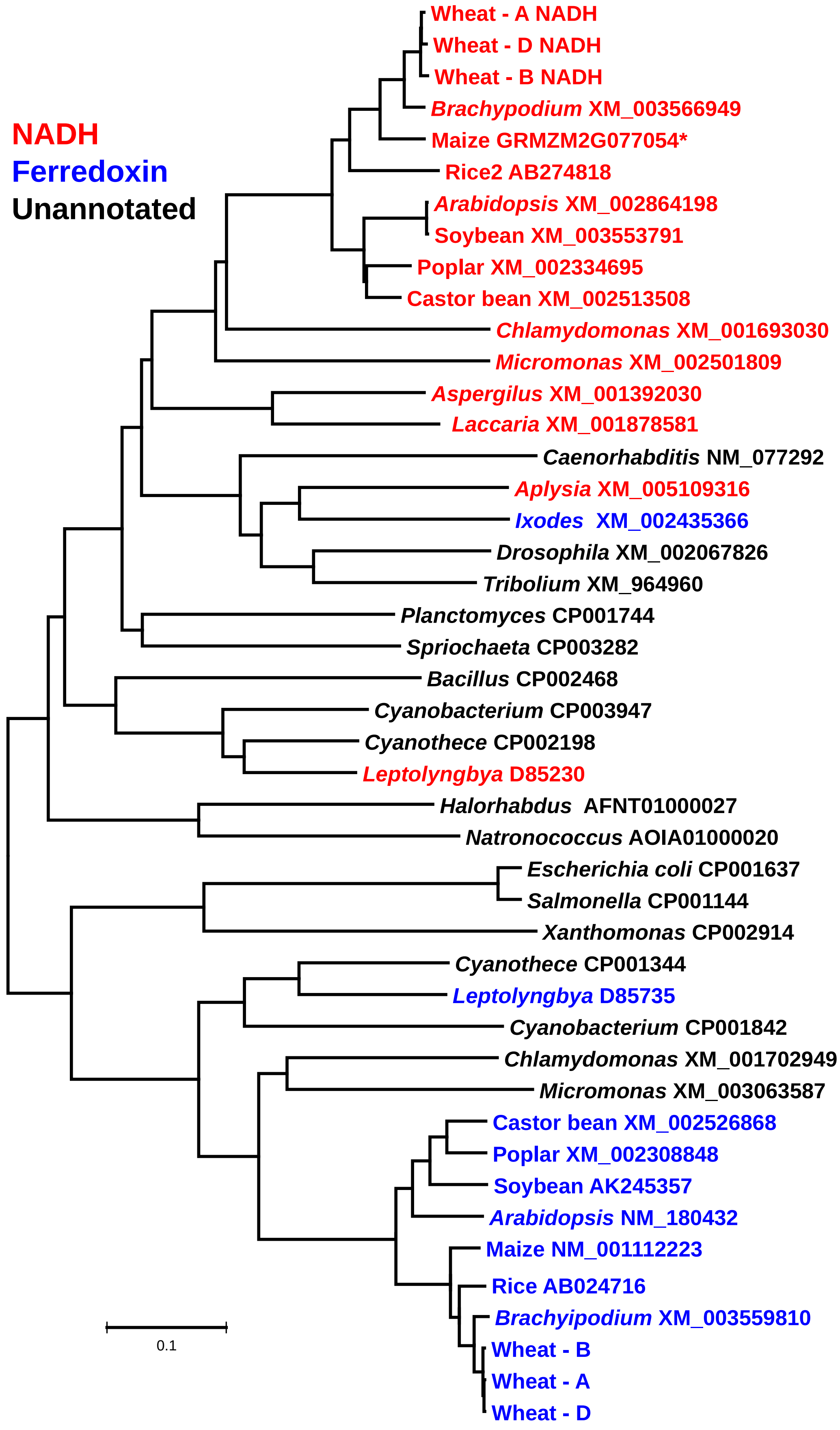
NADH
Ferredoxin
Unannotated
Wheat - A NADH
Wheat - D NADH
Wheat - B NADH
Brachypodium XM_003566949
Maize GRMZM2G077054*
Rice2 AB274818
Arabidopsis XM_002864198
Soybean XM_003553791
Poplar XM_002334695
Castor bean XM_002513508
Chlamydomonas XM_001693030
Micromonas XM_002501809
Aspergilus XM_001392030
Laccaria XM_001878581
Caenorhabditis NM_077292
Aplysia XM_005109316
Ixodes XM_002435366
Drosophila XM_002067826
Tribolium XM_964960
Planctomyces CP001744
Spriochaeta CP003282
Bacillus CP002468
Cyanobacterium CP003947
Cyanothece CP002198
Leptolyngbya D85230
Halorhabdus AFNT01000027
Natronococcus AOIA01000020
Escherichia coli CP001637
Salmonella CP001144
Xanthomonas CP002914
Cyanothece CP001344
Leptolyngbya D85735
Cyanobacterium CP001842
Chlamydomonas XM_001702949
Micromonas XM_003063587
Castor bean XM_002526868
Poplar XM_002308848
Soybean AK245357
Arabidopsis NM_180432
Maize NM_001112223
Rice AB024716
Brachyipodium XM_003559810
Wheat - B
Wheat - A
Wheat - D
0.1

Figure S5.

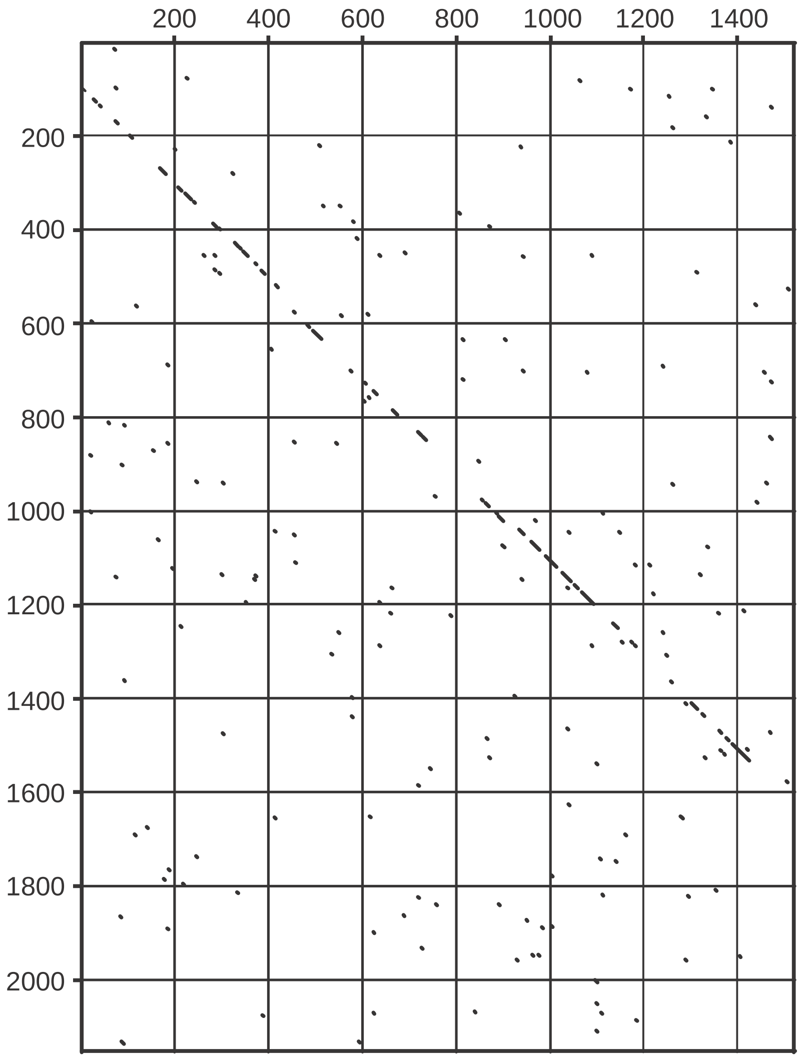

200
400
600
800
1000
1200
1400
200
400
600
800
1000
1200
1400
1600
1800
2000

Figure S6.

Text S1

>Chinese Spring A-genome
CGCACCGGCCGGGCGCCCTNAGTAAGCCCGCNCAGGAGGTATGGTTACGCTCTGCTCCCC
ACCCGCCGATCAAGTGCTCGTCTCGCGATTGGGTTTGGTTTCGGGGTTGGGGGTTTTTGT
AGGTTCGGATTGTTTGGTGCGATTGTTAGATTGGTTTATTTACGAAGTCACGGGCCATGG
CTTGTTTTGACGGATCATGGTTCTTTTATTTATTTGCGCTGTAATATGCTTCTGATAGCT
ATACGGTTGCCAAATTTTCGCGTTGGGAGATATGGGGGAGATCAGGATTCAGGAGACCCA
GGTATCTCCTGATTTCTGTATCGGACGATAAGGTTCTATGTGTTGACTCCAAAGTTCACT
GCATGTGCTTGTTTTATTAGGTCTCTAGCTCATGATGATGCACTTGCATACTATCGCTTT
TGTTATGTGGCGTTCCTTTCATGGGATTTTTATAGTACAAAGCGCAGCAAGTGCACAAAA
CACAGCAAATGCAGGTGCAATAAACAAGTGTGTGGTTATGAAAGGTTAGGTGACAGCTGT
GGTGGTAGGTACTAATCACGACCAAGTTCCAGGAGTACGACATTGTGTGATGCATAGGTG
GAGAATTTGCAACGTACTAGTACTAGTTTTCCATTTCCGTTCTGGAATGCGTTTGAATGG
GCTGTGACCTGTGAGCTGTGACAGTTATGAGTAATAACAAACCATATGTGCTGTGCTTTA
TATCTGACATTAGGAATTCCTTTTTTTGCCAGGCTGCGGATTTAAACGACATCTTAGCAG
AACGTGGAGCTTGTGGTGTTGGGTTTGTCGCAAACTTGAGTAATGAGCCTTCGTTCAACG
TTGTCCGTGATGCTCTTACAGCTCTTGGGTGTATGGAGCACCGTGGTGGCTGTGGATCCG
ACAACGACTCTGGTGATGGGGCAGGATTGATGAGCGGTATACCATGGGACTTGTTTGATG
ACTGGGCCAGCAAGGAAGGGCTTGCTCCTTTTGAAAGAACACATACAGGTGTAGGCATGG
TCTTCCTTCCACAAAACGAGAATTCCATGGCAGAAGCAAAAGCTGGTAATGATTTTGATG
CTTTAGCACCATGCTTCGTCCCCATTGTGGATCATTTCTTGTCTTTCTCTTTTGCTGGGA
TCTTGTCTTTAAAGTACGACACTTCCTAATTTTATATGGTCAGCGATTTCTGCTTGGCGA
TGCATGTCCTAGTAAATCTGACTCCGATCTCTTCGGACTCGTAATGCTGCCATACTTTGG
TTGCCATGCCATCGTGGTGCTTGAAACCTACGCTTGGAAAGTTCTTTTGCAGGAAAAGTT
TATGCCCTTGGTATAAGAACTTTCCAAAAGGAAATTTATTATTTTGTATTGGCAGTCAGA
TTACACATGTGTTTTTTCAGCTCTACTTGTACCACAACACACATAGCTTCCTAAGAGTCT
ATTCATTTTGCAGCTGTTGAGAAGGTTTTTACAGATGAAGGCCTTGAGGTTCTTGGCTGG
AGACCGGTTCCTTTCAATCTATCAGTGGTAGGCCCCAATGCAAAAGAAACAATGCCTAAT
ATACTCCAGATATTTGTGAGAATTGCGAAAGAAGATGACGCTGATGACATAGAGAGAGAA
TTATACATCTGCCGAAAGCTGATAGAGAGGGCTACAAAATCTGCTAGTTGGGCAGACGAA
CTATATTTCTGCTCTTTGTCAAGTAGAACTATCATTTACAAGGGAATGCTTCGATCTGAG
GTTCTTGGGCAGTTCTATTTGGACCTTAAGAATGAACTGTACAAATCTCCTTTTGCCATA
TATCATCGAAGATTCAGTACCAATACAAGCCCTAGATGGCCTCTTGCACAACCAATGAGG
TTGCTTGGACACAATGGAGAGATTAACACGATACAGGTTTACATTAACTTCTTCAACTTA
CTGTTAATCTTTACTCCCTGTATCTTACATTATTGTTTAGTGTCTTCCTTGATGCTCGCC
ATTTGTTTGATACTGAAAACTAGACATCCTTTGGGCATTACTCATAGCGTTCCTTTTCTT
TGCAGGGAAACTTGAACTGGATGCGATCAAGGGAAGCCACAATACAATCTCCTGTATGGC
GAGGCCGTGAGAATGAACTACGCCCATTTGGTGACCCTAAAGCATCCGATTCAGCAAACC
TTGACAGTGCTGCTGAAGTACGAGACTGCACACCGCTCATTAATATCGTAACATAAAGCA
TGCATTTCTTTTGCTTCTTGTCTGTACAACCGCGCCATTCACACGATTTTGCATACTTGT
CACTACTAGAATATTCCAAGGCCTTCTGGGCTCTGTTGAACATGCTTAACGACACATAAC
TCAGACATCTGCCAAGCTGTTACAAGAGATTAACAATTTGGCTTTAATGGGTACTGAATA
AGCTGTTACTTTAACTTGACTACCTTTTGCACCTTGATTTAAAGAGATGAACTGAACTAA
AAGATAAGTAGTTTCATAAATTTCAAACATACATGCAACATTTATGTTATTTTGAAAGCA
AAACTTTTGAAATTTTGGATAGCGAAGTGGTACTTTGACTACTCGTATTAATGCTAGAAA
CATGCAGTTGTTGCTAAGTGGCAATGTGACATATCTGTTTTCACTGTGTATAACAAATCA
TATATGCTATTCTCAATGTGCATTAAGCACCCCTGCCTTTATTTCGAGTATGTATTTCAA
CCTTAATTACTGGTGTGTATTTGTTGAGTCATCACATCCTTTCTTGCACCAGTTACTGTT
AAGAAGTGGTAGAAGTCCTGCTGAAGCTATGATGATGCTCGTCCCCGAGGCATACAAGAA
CCATCCGACATTATCAGTAAAATACCCGGAGGTATGGGATGCATTAGTGAACATATATTG
TGGGCATGCATGAATTCCCTTCCATATTAACTTATGCAAACAATTGTGTCCAATTGAAGC
TTCTCTTGGTTTGTGTATTAGGTAATTGACTTCTATGAATACTACAAAGGTCAAATGGAG
GCTTGGGATGGGCCTGCTTTACTTTTGTTTAGGTAATATCTCGTCGATCTGTGATTTGCC
GTATTATATTTTTTACTATCTTTATTTCTGATGCAAAGACAGTTTCTTTACTAGTTTGG
TTAAAGTCATTTCATGCATTAGCAGGTTTTGCTTCCTTCTCACTTTGTCCCTCTGTTTCT
GAATAGCCGTCATTTAGAAGTTTTGCAAAACAACCAGGGCACATATAGGAAAGACCACAT
TACTCCCTTTCATGGGGTTACTTGTTATTCTTGGGATGGTATGTTAGTCTACTCCATTGT
AATAAGTGACATCAGAAATGGGCTAATGTTAGGGGAATACAAGGAAACAATGGCCCAAAC
TTATCTTGGAGTCATAGCTAGTTTTTTAGGCTTAAGTACGTGTAGCCTTTAATCCTTTAT

TTAACACATTCACAGTTTGACCTGCAATTAGGAGCCAAATTTGTGTGCTTATGGCATGTA
TTGGGGAAGGGAGCTAGTATATACCTAACTGCAGGAACTAAAAGATATGGCAAATAGGTG
TTTAATTGCAAAGAGAGAGAATCTGAACACAGAGATGGGTATGTGCTAGCATCAGAAGTT
GTGGACAATATTAGTTGTAGAAGTTGAAACCTCTGACAGAGAAGTTTCCAAACATGTATT
TGTCGTCAAACAGAGTAGGGAAAATAGCTGTAATTTTTCCTCCGTGCCGTGTTATTCAAA
GCATAAGATATCCAAATGCTGCTTTTTCCCAACTGAGTAATTTCTTGCAACAGAGTGGGC
TAGATTAATATATTCTCTTTTGCATACCTCTATGTAGTGACGGAAGGACGGTAGGGGCAT
GCCTTGATCGAAATGGGCTGCGCCCAGCACGCTATTGGAAAACATCAGATGGTTTTGTTT
ATGTTGCATCTGAGGTAGGTTCAAGTTACTTCTTCTACTGTCTGAAATTTCTGTTTGGTT
ACAATGTTATGGTAGAGCGATTTTATATCACACATTAGTGAAGTGTCCATTTTGAAATTC
TGAAATTGGAGCATTTGGTTTATCTGCCAATAACTTATTGCAGATATACTGACATCCTTC
TATACCAAATCGCAGAGTTCAGATGTCACAAGCTAAACTTAATGTAATATTCAGTAAGTA
TTGTGCCTATTTGTTGAAGTGTCAGGTCTCACAGCTACACTAGGTGATTAGGACAAATTA
TGAAGTTACTTGTTAGTGTTTGGTTGCTATTTGAATGCTATGTCGCTCATGCATTTGTGC
CAGCAAGCTTCAATGATCTTATATGGGTTTCTGTTCTTGCCTTTGTGGCTACAAATTCAT
TCTACGGTGACAGATCATGTAAACCTTTGTTTGAGTAGAGTTAGATATATCAATCTGTTT
TTTGCGTTCCAAGACTTGACTTGGATGTAGGCATGTAGTAATAACTTGACATTTTAGCGT
ATTTTTGGGCTACACTTCTCAGCTTGCTCTCTCATATTGTGTAGTACTTTCATCCTACTA
GAGAGTATACGCAGTACCAAGATTAGAATTACTTTTTTATCAGTCTGTTTTTTGCGTTGC
AAGACTTGACTTGGATGTAGGCATGTAGCAATAACTTGGCATTTTAACGTATTCTTGGGT
TACACTTCTCAGCTTGTTCTCTCATATTGTGCAGTACTTTCATCCTACTAGAGAGTACAC
AGTACCAAGATTGGAAGTTATTTTTTTATGCTCGCTTTGTCTTGAATAAACTCCCCTCAC
ATGAGATCCTTTGTTCTGCTGATGCGTATACTCAGAGAGTAAAAGATAAAACTGTTAGTT
GTCATACATGATGCTTCTTATGGTTGTAATTTGACTAGTCTAGCTTATTAGATGTGAATT
TATACTCTAGTAATGCCTTTACCATCTCTTGGGTAGTTGAATCTGATCTGGTTATTTTAA
AAGCATGTCATTTAACTACATGCTAATAATTGACGAAGTAACCTTGGATCGCATTCTCAA
ATGTTTTTCTGTTGTTGTGTGGAAACATTCTCAAATGTTCTACAAGAGTTTGGCAAGTAT
TACTTTGTAGGATTCTCACCAACTGACTAGCTGTATTAATGAAAATCTACAGGTTGGTGT
TATACCGATGGATGAGTCGAAGGTAGTAATGAAAGGAAGATTGGGTCCTGGAATGATGAT
AACAGTTGACCTAGAGACTGGTCAGGTTAGAATAGAATAAAACAATCTTTTCCTTGTCTC
GCCTTTGATCATCAGAGTCTTATAAAATGTAGATTCTAAACCTCACGAGTCATCCCAGGT
CCTTGAAAATACAGAAGTGAAGAAGAATGTGGCTTCAGCAAAACCCTATGGAACTTGGCT
GCAAGAAAGTACACGTTCAATAAAGCCTGTCAACTTCCAATCCTCTCCTGTCATGGACAA
TGAAACAATTCTGAGACATCAGCAGTAAGAACCATGAAATATTGCTCTATTGAACTCCTC
TTTACTTTTCCCCTTGTACTTATTTGCTTATGTTTGTTCCAAGAGTTAGCATGTACTCCC
TCCGTTCCGAATTACTTGTCTTAGNNNNNNNNNNNNNNNNNNNNNNTATGTATGTATGTCTG
GCAATATGCCTGATGCTTATGTTGAGAAGGTTGTGGACTTGTGGCTGACTATTTCAGTGA
TGGTATATTGCAATGCCTTTTGCTCTTTCTGTGTTATGTAAACCTTTTTAAACTATATAC
ATAAAATCATTTGGGTCAACATATTGTATCCATCAAAAAGATTTCCACAGATTACATTAT
GGAATGTCTGAAGTAAACCTAGCTTGATACAGAAGTATAGGGCATTACTTCTTTTTTTCA
GATTTCAGAAGATAAACCCCAAAAATGTTACTTTTGATCATGTCATGCCCAGAAATATAC
TGCCAAAGAATGTGGCTGATTTTAACGTGTGCTCCATGTCTACAACTTACCTATGTCGTT
GTTTCAGACATTGTAAGAATTAGAGATATTAGATGGTCAGTCAGATCTGATGTTTAATTT
AATGTTATTATATTTTTTAGGGCATTTGGTTATTCCAGTGAAGATGTGCAAATGGTAATT
GAAACAATGGCTTCACAAGGGAAGGAGCCAACATTTTGCATGGGCGATGACATTCCATTA
GCCGTGTTGTCACAAAAGCCACACATGCTCTTTGATTATTTCAAGCAGCGATTTGCACAG
GTCAGTAAATCCTCAACATTTCAGTAATAGTGTTAAAAAATCTAGGTTATGAAACTTCCT
CTATTACCCTTAAATACTTGCACGGTATTGACAGTCCTTGGCAAAAAATTCTTATCCACA
GTACAGTTAGTTGGGAAAATCACATCTCCGTAGTCACTATTTCTAAGGTTATTTTTAGAA
AGTAATTAATTAATTCCATCCAATCTTCAATCCATTCAATTATAGGGATGGCTTGATTTT
AACCCTAAGAATTAATCTCCATCTTCATAGAAACATACCTTAGACGGTTTATATCTATAT
GGAGGTGTAAATCTGAGTACAATATATGATACTATCAATAAGGAAGTAAACTGAACAAAC
AAGGGAACTAATTAGTGTGATCTCTACTTGCCAATTAATACTGCACCAGCTGGGCCTGCA
TGAACAGTGTGCAGGTGCACCGTGGCTTCACATCAGTAATGGGTCCTCATTTACAAATTA
TCTTATTAATCAAAGTCATGTCAGCTTTGTCTTATGAATGTATCTCATTCAATTTCTAGG
TCACAAATCCTGCAATTGATCCACTCAGAGAAGGTTTAGTCATGTCTCTTGAAGTTAACA
TTGGTAAACGAGGCAACATCTTGGAGGTTGGCCCTGAAAATGCTGACCAGGTTAGTTAGT
TTTGAAGGTAATGATTTGACCTGTATTCTTGGGGCAATATTTTGATATCATCTTATTGAC
TTCTCAGGTTACCCTATCAAGTCCTGTCCTGAATGAAGGTGAACTAGAATCACTATTGAA
GGACCCAAAATTAAAGCCCAAAGTACTCTCAACATACTTCAATATCCGTAAAGGTCTAGA

TGGTTCCCTTGAGAATGCAATTAAGGCTCTTTGTGAAGAAGCTGATGCTGCTGTGCGGAG
TGGATCTCAACTTCTTGTGCTTTCCGATCGTTCTGAAGCACTTGTGAGTAGCTTCCGACT
TCTTAATGTGTTCACACAGATATTTACAGCAATCTTTATGCTTCTTGTTTATAGACTCGA
ACTATATTTTAAGTAGCTTTCATATTCTTGTTCACTTTTCTTTTTCAATGGGTAAGAAGC
AGTGAATGTGCTTGGTTTGAACTTAAATTATTTTTTTACTTCGCTCCATAATGGGCAATA
CTGATTTGTCGAAGTGTACCAGATCCAAATACATGTATACTTACTATCTACATCTATGGT
GCACCATACAAAATGATCTACTGGTACCTCGATTAGTGTCAGTTAGTTACTAGTCATGGG
CTACCTTTGTCTAATTATGCATCCACCAGGAGCACTATTCACAATATATGATAGTACCAA
TCTATATAATTTGAAGGGCTGGACTACAGCGGCGCTTAACGCGCGACCTGTCCTAGTTTG
GTTGTGTTGGCTAGTGCATGAATCTTAACATGGTATCGGAACCTACGGTTTCGAGTTCAA
GTCCTGTCTTTTGCAGTTTATCCAAAAATTAACCTAAGAAACTTAATTTATACCAAACTG
GACACTTGGATATCAGAATGTCTTTATTGTCACAATTTATGTACACATAATTGTGCAAAA
TAATATAAGACACCCGCGCATCAATATGATATGCCTATAATGATTGATGCATGTGGATGT
ACATGGTAGCTTCTTGTTGGTGACACCATTATCCGTGTCCTCTTCACCTCATAATGGCTT
TCTCTATCTTTGAAGTTTCATTGAAATATCCAGCTTGCTTCATGAATAGCCCTTCGCTTC
TTGTCAAATGTCTGACCGGCTGTCTAACATTGCCTGCCATATCTAGTAATATGATTTCCA
CCTTTAAACTATTTGGTCTTGTGACCAGAAAATACAGACATTACTTTTCCTGACATACAG
TTACTTGAAAACATCACGATCTAGTGCTAGGTTATGAAGCCATTATTTTTTTTGTTCAAC
TTCAGAATCTCTCTTGTGCGATGCAATACACTATGGAGATAGAATTACACACTTCCTGTA
TTGCAATTAAATGAATGTATAATGCAGGAACCAACACGGCCTGCTGTCCCAATACTTTTA
GCTGTTGGTGCCATCCACCAGCATTTGATTCAGAATGGCCTCCGCATGTCAGCTTCAATT
GTTGCTGACACTGCTCAGTGTTTCAGCACCCATCAGTTTGCCTGTTTGATAGGATATGGA
GCCAGGTATGACTTATATTTTGAAGAAGATTTGTTTAGTAACAGCTAGATCATTACTTAT
GTTCGCTTCACTTGACATTCTGATCCAGCATAGATGAATATATAAACTCCCACTCTGTGT
TAGTTTATGAACATGATTATTAAACTAGCATGACTGTTGAATACATACAATGCAATCAAG
GTGGACTAATGTCATTCACCACTTTTGGCTGCTTATGTCTTTGCATGTCAACTTTTTGCA
ATATATATTTATGATTTCTGTCTTCAGATATCCAATACTATCCTATTCTAGGATCACATA
GCAAATCTTCTGGGAGTAGCATCGAAATAAATTATCAACTGAAGTATTTGATAAAACATC
AAACTGATTCAGGTTTTTTCCAGAAGAAACAAATTCCCTTTTCAAGCCAGTGCCCCATAG
CCACTTGGCTAATATTTCCTTCGACAATTGGCGTATAAACGATAATTGTGAATCAGCAGT
CGATGTCCTCTGGAAACTGGGCATGCAGTTTGGACTTGCAGAATGAATTAGAACATAAGA
CAACTTGAGTCCTCGTACTAAGCCTTACTGAAATATCTGAATCATCATCCAAAAGAAGTA
CCGAGTTAGAGGAAGAAAGGATAAAAATAATTCAGTTAGATGAAGCAGCCACAATAGGTA
GAGGAATTGGACTTCGTTCCAGTTTAAATTAGCAAATTAGAACCTATTTGTTGGGAGGGT
TTTCTTGAAGATCCAATCATTTCTTTGGTTCTTCAGCAACTTGTAGTTAGAACTTATATT
CAGCTGTGCACATTCTCCAGGAACTATTTAGTTCTCAAGTTACAATGTTGTGTCAGTCGC
TAGATTTACTAATATTCATTCTTATGTTGCACATTAGTGCTATATGCCCATATCTGGCAT
TGGAAACATGCCGGCAATGGAGGCTGAGCAACAAAACTGTCAATTTGATGCGAAATGGCA
AGATGCCCACAGTGACCATCGAGCAGGCTCAAAGAAACTTTATCAAGGTATGATTTCTAT
GGCTTGTAAGTTGTGAGTTTTGACTAGTTTGTTGCATGTAGTTGTGCTGCTGCCCCTACC
TGTGTGAACATAGTTGGACTCAGTACTACTCCCTCCGTCCCAAAATAAGTGACTCAACTT
TTGCACTAACTTTACAAAGTTGAGTCACTTATTTTGGGACGGAGGGAGTACATTTGTGCA
TTAATGCTGTAGTTGATCGTTCAAGTGTGATCTTTGTGTTCCGTTTTACATCTGTTATTA
ATTGGTTTATGTTTTTGCAGGCAGTAAAATCTGGCCTGCTCAAGATACTCTCAAAAATGG
GCATATCCTTGCTCTCAAGGTTTGTCTTATCTTTGCCATCCAGTGTAATTTAATAATGGT
GCCAGTATGTTGAAGTACTTCTGCAATTGTTTAATGGGTCCACATCTTTTGGATAGTTAC
TGTGGAGCTCAGATCTTTGAAATATATGGTCTTGGCCAAGAAGTTGTCGACCTTGCGTTC
TGTGGGAGTGTATCGAAAATTGGAGGACTCACCCTTAATGAGGTAATTCACAATTATGCA
TATAGTCATGCAACACCTTTCTTTTTGGTGTTGATATTCTTGTTGACCTGGTACATCGTC
TGTCTAGAAGAGTGTTTAAATAGATTAACACCTGAGTTGGTTCATGTACCTTGTTATTTC
TTTTGAGACTGTTACCTACTGATAATTCTCCATTTCTTTATTCTATCCTTTTCTTGTAAA
GCTGGGTCGAGAAACACTATCATTCTGGGTGAGGGCTTTCTCAGAAGATACCGCAAAGAG
GCTGGAAAACTTCGGATTCATCCAATCCAGACCTGGAGGTAGTATCTCTTTCATCAGCTG
TAACAAAAAGTTAGGTTTTGTGAGTTGGGTGCCTGTGTTCTGCCAATTTTTAATCCAATA
TGAGTTTTTAATTAAAATGACACCAATTAACGACCTGAGATCTGGTTGCGGCAAATTGTA
CGTGCAATCAACACATACAAAGGTCACAACTAATTGAGAAATCTATGTTCCTCTTGAGTT
CTACTTTCGATTCACCAATAGCAAAGTGTCTTCATGGATCCTCTCGTCTCTAGTTAATTT
GTTTTGTTGAAAAGGAGGTGTACTGGAGAGGCCCTTGGTGTTCTGGGTCTTGTTTTTCAC
GGGTGTACTATTGCAATGTTTGGACTTGTCCTAGCCAACCAGGGATACAAAAATGCTTAT
ATCTTAACGTGATAGCTAAATCCATTTACCTTTTTTAGCGTGATAGGTACTAAGATATGT

CCATATAATGAATACTGACAATACCTCCTGACTCCTCCCTAAAGTTACACTTGTTGCTGA
AGGAGTTGCATTTTAATAAATTTGTCAGATCATATGCAGTGCAATGTTATCTTACTGTTT
ATTGCTGAAATAACATGGTGCCTTTTGCTCCCTGAATAGGTGAATTTCATGCAAATAATC
CTGAGATGTCAAAGCTGCTGCACAAAGCGATTCGTGAAAAAAGTGATAATGCATACACCA
TCTACCAACAACATCTTGCAAGCCGTCCTGTCAATGTTAGTGGTGCTTATCGTCACTTAG
CAGGTGGGCAACCTGTAGAATTTTTCGTTATCACCTTTGGTTTACCATATCTTCTACGCT
GCAGGTTCTTCGAGATCTTGTAGAACTAAAGAGTGAACGGACGCCTATTCCTATTGGCAA
AGTTGAGCCTGCAACCTCTATCGTTGAGCGTTTTTGCACAGGTGGAATGTCCTTGGGTGC
TATTTCTAGAGAAACACACGAAGCTATTGCCATTGCAATGAATAGGATAGGTGGGAAATC
TAATTCAGGAGAAGGTGGTGAGGTGGGTCCTATAACTATCCATTAGAAGAATAAAGCTAT
TTAGTTATGTGAAGCAGCTCCAGTGGAAACTGCAACATAGAGCTGCTTATGGACTTGCTT
ACTTTTGCAGGACCCAATCCGCTGGAGTCCCCTTACAGATGTTGTTGATGGGTATTCTGC
GACACTCCCTCATCTAAAAGGTCTTCAGAACGGGGACACCGCCACAAGTGCCATTAAGCA
GGTGCAGCATCAACTGCTTTATTTTTGCACTTTACCTTGGCTATTATATACTCCCTCCGT
TCCGAATTACTTGTCGCAAGTATGGATGTATCTAGATGTATTTTAGTTCTAGATACATCC
ATTTCTGCGACGAGTARTTTGGAACGGAGGGAGTATAATTTTGATGCAGATTACGCTTCT
ATTCTCAAAGCCATGATTATGGGTTCCTCTAATTATCACTGGGAGACACCACACTCTCGT
AATGGCTACATTATCAATTCTTCAGTAAATACTGAGTAGGATTCCACTCAATTTTGTTAT
GATAGCATGGTTAAAAAGAGCACTTTGAAAACCAATGCTGTGAACAAGCCCAACCTTATT
CGAGATTAAGTTATGTATCTAGCCAATTCTCCTTTAGAATACGGGGAAATTCACACTGTT
GAATATGCTTGTTTTCCTGTAAAATGGGGCCAGTACGAGTCTTTTCAGGCCAAGATTACC
TTGGGGGTTGAACATCAAAAATCTTCTATCACTACATCACTAGAAGGTGTTTACTGGTTT
GCTACTGTAACTGAATGGAATTCGGCACCTGCACTTCTGCCCTGGAGGTATCTGTTTCCA
GAAGGAATTGGAGGTCTATCAGTGCTAGTTATGTGCGGGTGCTAGTGTCATATGGTAGTC
AGCATTCTCAAACGTGAAACTCTGCAGTGGCAGCATATCCAAATTTTCTTTATTATCATG
TTTCCTGCTTTTCTGAAAAAGGAAAGAAGTAGAAGCATGTTTTGGTACTTTCATCTGCAC
TGTAGTATCAAATACGTAGACGCATATTGTCCACCTCTTTATTTATGTGCTGATGAATCA
AAGGTACACTATATCCACTATCCAGTGGGTTCATATATTTGAGTTGGTTCTCATATGTAC
TATCCTTTCACAGGTTGCATCTGGACGTTTTGGTGTTACACCAACATTTTTAGTTAATGC
TGAACAAATTGAGATAAAGATTGCACAAGGGGCTAAGCCTGGTGAAGGGGGTCAACTTCC
TGGGAAAAAAAGTCAGTGCATACATAGCTCGGTTGAGAAACTCCAAACCTGGTGTTCCTCT
CATATCCCCACCACCACACCATGACATCTATTCTATTGAGGATCTGGCGCAGCTAATTTT
TGATCTACATCAGGTTTGTTTTGCACAACTACTTCTACAATATCAACTAGTAATGTTGGT
GCTAAGGGAAAACTGCTAGTTTTAAATTAAGTTTTATGTGTGTCACATAAGAATTAGAGT
CCTTAAGCTGCCTTATATGTTAGCTTCAAAATAAATTATGTGTGTCACATGAGAATTAGA
GTCCTTAAGCTGCCTATATATCAACTAGCAGTGCTGTTTAATTATTGATTTCTTTGAGAA
GGAAATCCATTTATTGGTACTAATCAAATATTTAATTCATATTTGACTGGGCAGATCAAT
CCTAAAGCGAAGGTGTCGGTAAAGCTTGTAGCTGAAGCAGGAATTGGAACTGTAGCCTCT
GGAGTATCTAAGGCAAATGCAGACGTAATTCAGGTAGATTAAACATCAAAATTTTAATAA
CTCGTTGATTTAGCTCATACCATTATTGTACGTGGATCTGTATCCCATAAGTGGCTATGC
TTCTGTGGTTTAGTGTTATTTTACATTTTTTTGTACCCTTAACCTTTTGCTGTATGGTGT
TATCTGCTAAGTTGGCCCTCATGAGGCTGCTTAAACCGCCTTTTGCTATTTCATGCTGCA
GATAGTGACATTTGCTGTCTGATGTTCACGATGATAGACCTTCCTATTGTAGTTCATTTT
TTAATGAAATATGAATTTCTCGCTCCCTGTGGATAGCCAGTTGTTAGACATTGCTATAGT
GGCACTTGTCCTGTAACAGACAGACATCACTTTTTAGTGAATTATATGCAATAATCTGTA
GTTTTTTTGGGGGGAAATCCCAGAAATCTAGTAAAATTTCCCAGAACACAAAAATTCCAG
AAATATAGAAATGTTTGTTGCACACACAGACACCATAAGGTAAGCAATGACTTATGAAGA
GGCCGATTTAGTTTAATGAATTGGATGAGCACACTAAACCTGTTGGTTGTTACATCATTG
AGAGAAACCATGCTATGAAATCTTATTTAATTTGTTGTGAGTGGTTTCATCCAAAGTCTA
CTGTACTCATGGGTGAACTAGAGGTTCAGTATACATATTTGGTGATCTAATATGCCATT
TTATTATACAGATATCAGGGCATGATGGTGGAACTGGAGCTAGCCCAATTAGCTCAATCA
AGCATGCTGGGGGTCCTTGGGAACTTGGTCTTACGGAAACACACCAGGTTTTCTACATTT
AATTGCTTGATATAATCAGTTTCTTTTAATACCAAATCACTTTCTTTGTTCTAAGCCTAG
TTAGCTTCCTTCAGACACTCATACAAAATGGATTGAGAGAGAGGGTGGTACTTAGGGTGG
ATGGCGGATTCAGGAGTGGCCTAGATGTCCTTTTGGCTGCTGCCATGGGTGCTGATGAAT
ATGGCTTTGGTTCTGTAGCTATGATAGCTACTGGATGTGTCATGGCACGCATTTGCCACA
CAAACAATTGCCCAGTTGGAGTTGCTAGTCAGGTAGTAGCAGCAGAATTGATTTTTTATT
TGTCCTGCTTGGCTATACCTTACATTCTATGCTCCTGCAGAGAGAAGAGCTACGCGCTAG
GTTCCCTGGTGTTCCTGGTGATCTTGTGAATTACTTCCTCTTTGTTGCAGAGGAGGTATG
GCATCACTCACATTACTAGTGCCTTTTCTGTTAACCTCTTTCTGTGCTTGATTTGCAAAT

TTGGTCATGCATATTTTTGGAGTAACCATGACTCATATTTCAAGTACTCATGCTTCTGTT
CCAGGTACGAGCCACATTAGCCCAGTTGGGTTATGAGAAGCTGGATGATATAATTGGGCG
GACAGATTTACTTAAGCCAAAGCATATCTCTTTGGTGAAAACGCAGCACATTGATCTTGC
ATACCTATTAATGGTATGGAGTTGATAGCTATTCTCATTTGAGTTATTGGCATTTCATGC
CTATCTTGTTATATTTATCGGTGTTCTCGTTTCAGAATGCTGGATTACCCAAATGGAGCA
GCTCTCAGATAAGGAGCCAGGACGTCCACTCTAATGGCCCTGTTCTTGATGAGACAATCC
TTGCAGATCCCGAGGTAATGTTTTTGATAGCTTTTCTTAGTTACGTCGATTAACAACTGT
CCTCCTGAGCAGATTTGTGTACACCCAATATTTTTTTGTCTCAGGAACAAAGAACTGTG
ACGTTGATCAATAGATATTGTGGCCTTAAAGTGAAATGCATAGTTGCTAGGAACCAAGCT
CTGCTGTATGCAGCATTACTTAAATTATATAATGGCATTTTGCAGGTATCCGATGCAATT
GAGAATGAGAAAGAGGTTTCAAAGACATATCCAATTTATAATGTTGATAGGGCTGTTTGC
GGCCGTGTAGCGGGTGCCATTGCTAAGAAGTACGGAGATACAGGCTTTGCAGGCCAGCTG
AACATTACGTATGTCAACTACTTTCTTGTCTGCATAATTCTGTGCCCTTTAACTTTTATT
ATGAGAAGATAGGAAATAAACAATTTCAAACATTTCAGGTTCACCGGAAGTGCAGGGCAG
TCCTTTGGTTGTTTCTGACACCTGGCATGAATGTTCGCCTAGTAGGAGAGGCCAACGATT
ATGTGGGAAAGGTACTAAATACTCAACTGGACTGAAAATACTGTCTGACAAGCAGCTTTA
TAATGCCTTCTATTTCATTTTCTACAGGGTATGGCTGGTGGAGAGCTGGTTGTAGTTCCT
GTAGATGATACAGGATTTGTTCCTGAGGATGCTGCTATAGTTGGGAACACTTGTCTGTAT
GGAGCTACGGGTGGTCAAGTATTTGTGAGAGGCAAGACAGGAGAAAGATTTGCAGTTCGG
AATTCTCTTGGGCAAGCAGTGGTTGAGGGCACAGGAGATCATTGCTGCGAGTACATGACC
GGTGGATGTGTAGTTGTACTCGGCAAGTAAGTACACAGTCTGCATTTTCCCTACTTCGTG
TATTACCCTTCTTGTCCTGATATATATTTCTCAATCCACAGAGTTGGAAGAAACGTTGCA
GCCGGAATGACCGGTGGCCTAGCCTACATGTTAGATGAAGATGATACTCTAGTCCCGAAG
GTATGCAAATTTGGAATCTGTAGCTATACCTTCAGCTGCCCTGTAGCATGCACATCTAGT
GAAAGCCTTGGATTCAAATCTCAAGATCATAACGTGCAAGTGTTTTACTAGGTAAACAAG
GAGATTGTCAAGATGCAGAGAGTGAATGCTCCCGCTGGGCAGATGCAGCTCAAGGGCTTG
ATTGAGGCCTATGTTGTAAGTCTAGTGCACTCTTTGAAAGTTTTGTGCCAAGGTTGGCCA
TTACCCATGTTCTTATACTTTGGATGTCGCTTTTATAGGAAAAAACAGGCAGCACAAAAG
GCGCTAAAATTCTGAGCGAATGGGAGGCATATCTGCCACTCTTCTGGCAGTTGGTGCCAC
CCAGCGAAGAAGACTCGCCTGAAGCTTGTGCCGAGTTTGAGAGAGTACTTGCCAGGCAAA
AAACGGCAGTACAATCTGCTAAGTGATACTAGCCAAAGCCCAATCGCCAAAATGACCAAA
GCTAAACGGAGGGAAAGCGTTCTTACTGCGATACTTGAAAGTGATTTTCTCAGACCGCGG
TCCTTCTGAAGCACTAACGTCACCAAAGGCTGCAATTTTGTGGAAGCTCTGTCCAAGGCA
TCTTCTGGAAACGAGCTTGCAGATACCGTCGAGGATTCTTCTGCACGCTCTTGCAGCCTA
GGTAACCAACCACCCCTGTAAATTGGTTCGCTGACGCTAGGCTGCAAGGCCCGGCCCCTG
TTGAACTGCTGCTTTCTTGTACATGATTCCTGGCTTCGCCTGTGTAATTATATTTTTTGT
TCTGCACACATTAGTTCTTATATTTATACCCGTCCTGTGTTAGGCATGCGAGTTGCATGT
GCTGTAGTTCAGTACAGAATAAGTAAAGTTTATGATGAAGTAGGACTTCTCTGGTGTCGA
CAATTGGTGGAACTTGTTTTTCTTTTTTTCTAAGTTTCTTACGGGGCGTTCAGCGAACTC
TWAACGATTTCCATTATTAACTCGATGGCGCCAGCGTCACCGAGGTTTGAGGTTTGACAA
CGTGTGAGAAGAGAGCCGGAAAGCTCGCGGCTAGGTTTCACCCGACAGCGCGGCGCCCAG
CTCGAGGACTAGCCATACAACGAGGTGGTTTGTTATCTACGATCGGACGTCCTATATCCA
CACCAAATCGTCTGGGTGGCCTGTCCGATTCGTGTGGGTGGCCTGTACGATCAGAAAAAC
GCCACCTAATCGGATCAGCCAAACTCCGCCTAGACGCCTTTGCTGACCGGTACTATCCAC
ACCTTGTCCATTTCCGGGGCGGATATAGAGACACTTGGGTCCGCAAGGGTTGCCTCGTCC
GCATAGATGGCACTGTAGCTCAGCCGCCATTGTTGCTCCGCCGCTGTCCCCGATCCTGAT
CCACAACACCACCCCAATCAGCAATTCCACTGCCCATGCCGTCGACGGACAAGATCACCG
CCATCAGATGCCACCATCGTATGTTGAAGTCTTTTGTTTAAGAGACTTTGTGATGGAGAA
GCTTCACTCCACAACTACACCGTTAATGGGCATAACTACAATAAGGGGCACCTTACCGAT
GGCATCAATCCTCAATGGACGGCGTTTGTGAAGACTATATCGGATCCCCATGATAGAAAA
ATATGTCTCTTTGCACAAGTGCAAGGTGCTAGGAAGGATGTGGAGCGAACATTCTGAGTG
CTCCAAGCTTGTTGGTGTATTGTTTGTGGAGCTGCAATGATGCGGGAAAGCGAAGACACT
TTGACAACTCATGACATGTTGTGTAATCTTGCACAATATGATTGTTGAGGATGAGGGTGA
AGGGGCAACCCGCATGCATGATTTTGAGAAGCCCAGAGTTCAGGTCACGTACCAAAACAA
TAGGCGGAGCATAGTGCCAACTTTCTTGAGATGTATCGACAACTTCAAGATCAACAAGCA
CACATACAATTTCTCAGTGATCTTGTGGAGCATATGTGGATCCACGCTGGAAATCATTGA
ACTATGCTGTTTATGTTTCAGTTATGCACTATGAACAAATTTTATGTTTGGACTATGTTT
GAATTATGTATTTTTATGTATGAATAATTTGTATGTGTGTGCTACTCATTGTTTGTGTGT
GATGATCAACGTGCATGTGTTTGCATGGATTTGAGGTTTCAAAAGTAGGATGGTTGCAAA
TGGGGACATTTGAGGGCTGGACCGCCGCTGCCCACGGTCACGCCGGCAGATGTATAGGGG

CATGGTGTGCTTGTAGATGCTCTTAGGGATTGGTTGGCCGGGGGGGGGGGGGGGGTTCAG
TACATGGAGATCATGGACACCATCTCACCTATGTCACGTAGCTTGCACATCTCCGACTGC
ACGATCTTGCATTGGGCGCTTTTTTCGCGAATACGCAAAGCTTGCGTATCTTCCATTGCT
AGTAGGGAGAGAGTTACAGAACCCGCTTAACGATCCAAAATTGGAAGTCCTAGCAGGTAT
GGGAGAGACTACATGTGGGAAGTGGGGAGTTACAATAGGACATGACTACCGCATGCAGA
CTGCTTAGATAAGATGTTGGCCAAGCCAAAAAAACCTGCTCTGAACCACAGACGAGCTTC
GTCCTGGATGAGCGAGAGAAGACGACGTACAAAGGGAGTTTCACATTGAACACACCGCTT
CTCAAGGAGATTGAAGCTGTGCGTGGGAAGGGTCCTGGATTGGAGTACACACCTCGCCGT
ACCACTCGTTCTGCGACTGTGAACGCGCCACGGAAGTCTAAAGCAACGACGGCTGAGACG
GTTCTTCTTAAAGCGCTAGGCATCACGCCAGAGGGCTTGGCGGTTACCGATGAGACCCAT
GCACAGCTCAGGCAGATGTTCGATTCTCCCATACAGGAACCGCAGCTACGTGCAATTGCC
TCGATTTTTGGCAAGGCCATCCCTTTTGACCTTGGCCAGGAGGTTCCCCGAAGGTGGCAT
TGCTTGCCTAGTTGGCGAGCGTTGGTGCATGTGTGGACAGTCATRGTCGTCGATCAAACT
GGGAGTTGGTGTGCTGGATGTGCGTGGCTTGAACNGTCCGGCGAAGAGAGGCGTTGCGTG
AATTTGCGGGACTCGACGCACCCGGGTCTA

>Chinese Spring B-genome
CGCGCCTGCGTCCCACCGCCGAGTCAAGTGCTGTCTCGCGATTGGGTTGTTCGGGGTGGG
TTTTTGAGTTCGGATTGAAGTGCGGTTATAGTAGATTGGTTGTTTACGAAGTCACGGGCC
ATGACTTGCTTTGTACGGATCATAGTTATTATCTTTTGTCGCTGTAATATGCTTCTGATG
GCAAACATTTTCGTTGGGAAATTTGGGCGAGACCAGGATTCAGGAGTCAAGCCCAGGTAT
CTCCTGAGTCCTGACTACTGTATCGGGTGATAAGGTTCTATGTTGACCTCAAAGTTCATT
GCATGTGCTAGTTTTTTTGGTCCCTAGCTCGTGATGATGCACTTGCATACTATCGCTTTT
GTTATGTGGTGTTCCTTTCATGGGATTTTTAAAGTACATAGTCTGGCAAGTGCACAAAAC
ACAGCGAATGCAGGTGCAATAAACAAGTGCGTGGTAATAAAAGGTTAGGTGACAGCTGTG
GTGGTAGGTACTAATCACGATCAAGTTCCAGGAGTACGACATTGTGTAATGCATAGGTGG
AGAATTTGCAACGTACTAGTACTAGTTTTCCATTTCCTTTCTGGAATGCGTTTGAATGGG
CTGTGACCTGTGAGCTGTGACAGTTATGAGTAATCATAAACCATATGTGCAGTGCTTTAT
ATCTGACATTAGGAATTCCTTTTTTTTGTCAGGCTGCGGATTTGAACGATATCTTAGCAG
AACGTGGAGCTTGTGGTGTTGGGTTTGTCGCAAACTTGAGTAATGAGCCTTCGTTCAACG
TTGTCCGTGATGCTCTTACAGCTCTTGGGTGTATGGAGCACCGTGGTGGCTGTGGATCCG
ACAACGACTCTGGTGATGGGGCAGGATTGATGAGCGGTATACCATGGGACTTGTTTGATG
ACTGGGCCAGCAAGGAAGGGCTTGCTCCTATTGAAAGAACACATACAGGTGTAGGCATGG
TCTTCCTTCCACAAAACGAGAATTCCATGGCAGAAGCAAAAGCTGGTAATGATTCTGATG
CTTTACCACCATACGTCGTACCCATTGTGGATCATTTCTAGTCTTTCTTTTTTGCCGGGA
TCTTGTCTTTAAAGTACAACACTTCCTAATTTTATATGGTCAGAGATTTCTGCTTCGCGA
TGCATGTCCTAGTAAATCTGACTCCGATCTCTATGTACTTGTAATGCTGCCATACTTTGG
TTGCCATGCCATCGTGGTGCTTGAAACCTACGCTTGGAAAGTTCTTTTGCAGGAAAAGTT
TATGCCCTTGGTATAAGAACTTTCCGAAAGGAAATTATTTATTCTGTATTGATAGCCAGA
TTACACATGTGTTTTTTTCACCTCTACTGTACCACAACACATATAGCTTCCTAAGAGTCT
ATTCATTTTGCAGCTGTTGAGAAGGTTTTTACAGATGAAGGCCTTGAGGTTCTTGGCTGG
AGACCTGTTCCTTTCAATCTATCAGTGGTTGGCCCCAATGCAAAAGAAACAATGCCTAAT
ATACTCCAGATATTTGTGAGAATTGCGAAAGAAGATGACGCTGATGACATAGAGAGAGAA
TTATACATCTGCCGGAAGCTGATAGAGAGGGCTACAAAATCTGCTAGTTGGGCAGATGAA
CTATATTTCTGCTCTTTGTCAAGTAGAACTATCATTTACAAGGGAATGCTTCGATCTGAG
GTTCTTGGGCAGTTCTATAAGGACCTTCAGAATGAACTGTACAAATCTCCTTTTGCCATA
TATCATCGAAGATTCAGTACCAATACAAGCCCGAGATGGCCTCTTGCACAACCAATGAGG
TTGCTTGGACACAATGGAGAGATTAACACGATACAGGTTTACATTAACTTCTTCAACTTA
CTGTTAATTTTTACTCCCTGTATCTTACATTATTGTTTAGTGTCTTCCTTTATGCTCGCC
AAATGTTTGATACTGAAAACTAGACATACTTTGGGCATTACTCATAGTGTTCCTTTTCTT
TGCAGGGAAACTTGAACTGGATGCGATCAAGGGAAGCCACAATACAATCTCCTGTATGGC
GAGGCCGTGAGAATGAACTACGCCCATTTGGTGACCCTAAAGCATCCGATTCAGCAAACC
TTGACAGTGCTGCTGAAGTACGAGACTGCACACTGTTCATTAATATCGTAACATAAAGCA
CACATTTCTTTTGCTTCTTGTCCGTACAGCCGCACCATTCACAAGAGTTTGCATACTTGT
CACCACTAGAATATTCCAAGGCCTTCTAGGCTGTGTTGAACATGCTTAATGACACATAAC
TCAGACATCTGTCAAGCTGTTACAAGAGATTAACAATTTGGTTTTTAATGGGTACTGAAT
AAGCTGTCACTTTAACTTGACTATTTTCTGCACCCTTGATTTACAGAGATGAAAGGGACT
AAAAAAGATAAGTAGTTCCATAAATTTCAAACTGTCAAACATACATGCATCTTTTATGCT
ATTTTGAAGCAAAGAAATTTTGGATAGCGAAGTGGTACCTTGACCACTCGATTTAATGCT
AGAAACATGCAGTTGTTGCCAAGTGGCAATCTGACTTATCTGTTTTCACTGTGCATAACA

AATCATATATGCTATTCATAACCCCGCCTTTATTTTGAGTATGTATTTCTACCTTAATTA
CTGGTATGTAATTGTTGAGTCATCACATCCTTTCTTGCACCAGTTACTGTTAAGAAGTGG
TAGAAGTCCTGCTGAAGCTATGATGATGCTCGTCCCCGAGGCATACAAGAACCATCCGAC
ATTATTAGTAAAATACCCGGAGGTATGGGATGCATTAGTGAACATATGTTGTGGCCTTGT
GGGCATGCATGAAGTCTCTTCCATATTAACTTATGCAAACAATTGTGTCCAATTGAAGCT
TCTCTTGGTTTGTGTATTAGGTAATTGACTTCTATGAATACTATAAAGGTCAAATGGAGG
CTTGGGATGGGCCTGCTTTACTTTTGTTTAGGTAATATCTCGTCGATCTGTGATTTACCG
TTTTATATTTTTTGCTATCTTTATTTTCTGATGTGAAGACAGTTTCTTTACTAGTTTGGT
TAAAGTCATTTCATGCATTAGCAGGTTTTGCTTCCTTCTCACTTTGTCCCTCTGTTTCTG
AATAGCTGTCATTTAGAAGTTTTGCAAAACAACCAAGGCACATATAGGAAAGACCACATT
ACTCCCTTTCATGGGGTTACTTGTTATTCTTGGGATGGCATGTTAGTCTACTCAATTGTA
ATAAGTGACATCAGAAATGGGCTAATGTTAGGGAATACAACTATACAAGGAAACAGTGGC
CCAAACTTATCTTGGAGTCATAGCTAGTTTTTCGAGGCTTAAGTACGTATAGCCTTTATT
TAACACATTTAAGTTTGACCTGTAATTAGGAACCAAATTTGTGTGCTTATGGCATGTATT
GGGGAAGGGAGCTAGTATATACCTAACTGCAGGAACTAAATGATACGGCAATTAGGTGTT
TCACTGCAAAGAGAGAATCAGAACACAGAGATGGGTATGTGCTAGCATCACAAGTTGTGG
ACAATATTTAGTCGTAGAAGTTGAAACCTCTGAATGAGAAGTTTCCAAACATGTATTTGT
CTTCAAACAGAGTAGGGAAAATAGCCGTAATTTTTCCGCTGTGCCGTGTTATTTAAAGCA
TAAGATATCCAAATGCTGCTTTTTCCCAACTGAGTAATTTCTTGCAACAGAGTGGGCTAG
ATTTACATGTTCTCTTTTGTATACCTCTATGTAGTGACGGAAGGACGGTAGGGGCATGCC
TTGATCGAAATGGGCTGCGTCCAGCACGCTATTGGAAAACATCAGATGGTTTTGTTTATG
TTGCATCTGAGGTAGGTTCAAGTTACTTCTTCTACTGTCTGAAATTCCTGTTTGGTTACA
ATGTAATCGTAGAGCGATTTTATATCACATATTAGTGAAGTGTCCATTTTGAAATTCTGA
AATTGGAGCATTTGGTTTATCTGCCAATAACTTATTGCAGATATACTGACATCCTTCTAT
ACCAAATCGCAGAGTTACAGATGTCACAAGCTAAACTTAATGTAATATTCAGTAAGTATT
GTGCCTATTTGTTGAAGTGTTAGGTCTCACAGCTACACTAGGTGATTAGGAAAAATTATG
AAGTTACTTGTTAGTGTTTGATTGCTCTTTGAATGCTATGTTGCTCATGCATTTGTGGCA
GCAAGCTTCAATGATCCTACATGGGTTTCTGTTCTTGCCTTTGTGGCTACAAATTCATTC
TACGGTGCCAGATCGTGTAAACCTTTGTTTGAGTAAACTTAGATATATCAATCTGTTTTT
TGTGTTCCAATACTTGACTTGGATGTAGGCATGTAGCAATAACTTGACATTTTAGCGTAT
TTTTGGGCTACACTTCTCAGGCTTGTTCTCTCATATTGTGCAGTACCATTCATCCTACTA
GAGAGTATACGCAGTACCAAGATTAGTAAGTTACTTTTTTATCAATCTGTTTTTTGCGTT
GCAAGACTTGACTTGGATGTAGGCATATAGCAATAACTTGACATTTTAACATATTTTTGG
GCTACACTTCTCAGCTTGTTCTCTCTATATTGTGCTAGTACTTYCATCCTACTAGAGAGT
ACACAGTACCAAGATTGGAAGTTAATTTTTTTATGCTCGCTTTGTCTTAAATAAACTTCC
CTCACACCAGATCCTTTGTTCTGCTGATGAGTATACTCAGAGAGTAAAAGATAAAACTGT
AGTTACCATACATGATGCTTCTTATGGTTGTAGTTTGACTAGTCTAGCTTATTAGATAAG
AGTTTATACTCTAGTAATACCTTTTGACCATCTCTTGGTAGTTGAATCTGATCTGATTAT
TTTAAAAGCATGTCATTTAACTATATGCTAATAGTTGACGAAGTAACCTTGGATCACATT
CCCAAATGTTTTTTTGTTGTTGTGTGGAAACATTCTCAAATGTTCTACAAGAGTTTGGCA
AGTATTACTTTGTAGCATTCTCACCAACTGACTAGCTGTATTAATGAAAATCTACAGGTT
GGTGTTATACCGATGGATGAGTCGAAGGTAGTAATGAAAGGAAGATTAGGTCCTGGAATG
ATGATAACTGTTGACCTAGAGACTGGTCAGGTTAGAATAAAATAAAACAATCTTTTCCTT
GTCTCGCCTTTGATCATCAGAGTCTTCTAAAATGTAGATTCTAAACCTCACGAGTCATCC
CAGGTCCTTGAAAACACAGAAGTGAAGAAGAATGTGGCTTCAGCAAAAACCCTATGGAAC
TTGGTTGCAAGAAAGTACACGTTCAATAAAGCCTGTCAACTTCCAATCCTCTCCTGTCAT
GGACAATGAAACAATTTTGAGACATCAGCAGTAAGAACCATGAAATATTGCTCTATTGAA
CTCCTCTTTACTTTTCCCCTTGTACTTATTTGCTTATGTTTGTTCCAAGAGTTAACATGT
ATGTATGTATGTCTGTCTGGCAATATGCCTGATGCTTATGTTGAGAAGGTTGTGGACTTG
TGGCTGACTATTTCAGTTATGATATATTGCAATGCCTTTTGCTCTTTCTGGGTTATGTAT
ACCTTTTTAAACTATATACATAAAATCATTTGGGTCAACATATTGTATCCATCAAAAAGA
TTTCCACAGATTACATTATGGGATGTCTGAAGTAAACCTAGCTTGATACAGAAGTATAGG
GCATTAGTTTTTTTTTCAGATTTCAGATGATAAACCCCAAAAATGTTACTTTTGATCATG
TCATGCCCAGAAATATACTGCCAAAGAATGTGGCTGATTTTAACGTGTGCTCCATGTCTA
CAACTTACCTATGTTGTTTAAGACATTGTAAGAATTAGAAATATTAGATGGTCAGTCAGA
CCTGATGTTTAATTTAATGTTATTATATATTTTTTAGGGCATTTGGTTATTCCAGTGAAG
ATGTGCAAATGGTAATTGAAACAATGGCTTCACAAGGGAAGGAGCCAACATTTTGCATGG
GTGATGACATTCCATTAGCCGTGTTGTCACAAAAGCCACACATGCTCTTTGATTATTTCA
AGCAGCGATTTGCACAGGTCAGTAAACCCTTAACATTTCAGTAATAGTGTTAAAAAATCT
AGGTTATGAAACTTCCTCTATTACCCTTAAATACTTGCACGATATTGGCAGTCCTTGCCA

AAAAATTCTTATCCACAGTACAGTTAGTTGGGAAAATTACATCTCCATAGTCGCATTTCA
AGGTTATTTTGAAGTAATAACTAATTCCATCCAAATCTTCAATCCATTCAATTATAGGGA
TGACTTGATTTAATCCCTAAGAAATAATCTCCATCTTCATAGAAACATACCTTAGACGGT
GTATATCTATATGGAGGTGTAAATCTAAGCACAAGATATGATACTATCAATAAGGAAGTA
AACCGAACAAACATGGGAACTAATTAGTGTGATCTCTACTTGCCAACTAATACTGCACCA
ACTGAGCCTACATGAACAGTGTGCAGGCGCACTGTGGCTGCACAGCAGTAACGGGTCCTC
ATTTACAAATTATCTTATTAATCAAAGTCATGTCAGCTTTGTCTTATGAATGTATCTCAT
TCAATTTCTAGGTTACAAATCCTGCAATTGATCCACTCAGAGAAGGTTTAGTCATGTCTC
TTGAAGTTAACATTGGTAAACGAGGCAACATCTTGGAGGTTGGCCCTGAAAATGCTGACC
AGGTTAGTTAGTTTTGAAGGTAATGATTTGACCTGTATTCTTGGGGCAATATTTAGATAT
CATCTTATTGACTTCTCAGGTTACCCTGTCAAGTCCTGTGCTGAATGAAGGTGAACTAGA
ATCACTATTGAAGGACCCAAAATTGAAGCCCAAAGTACTCTCGACATACTTCAATATCCG
TAAAGGTCTAGATGGTTCCCTTGAGAATGCAATTAAGGCTCTTTGTGAAGAAGCTGATGC
TGCTGTGAGAAGTGGTTCTCAACTTCTTGTGCTTTCCGATCGTTCTGAAGCACTTGTGAG
TAGCTTCTGACTTCTTAATGTGTTAACACAGATATCTACAGCAATCTTTATGCTCCTTGT
TTATAGACTCGAACTTTATTTTAAGTAGCTTTCATATTTTTGTTCACCTTTCTTTTTTAA
TGGGTAAGAAGCAGTGAATGTGCTTGGTTTGAACTTAATTTGTTTTTACTTCGCTTCATA
ATGGGCAATACTGATTTCTAAAAGTGTACCAGATCCAAATACATGTGTACTTACCATCTA
CATCTATGGTGCACCAGTACAGTACTCCAACAAAATGATTTACTGGTACCTTGATAAGTG
TCAGTTAGTTGCTATTCATGGGCTACCTTTGTCTAATTATGCATCCACCAGGAGCACTAT
TCACTTGAAAACATCGTGATCCAGTGCTAGGTTATGAAGCCTTTATTTTTTTGTCAACTT
CAGAATCTCTCTTGTGCGATGCAATACACTACGGAGATAGAATTATGTATTTTTTTTACT
ACACTTCCTGTATTGCAATTAAACGAATGTATAATGTAGGAACCAACACGGCCTGCCGTC
CCTATACTTTTAGCTGTTGGTGCCATCCACCAGCATTTGATTCAGAATGGCCTCCGCATG
TCAGCTTCAATTGTTGCCGACACTGCTCAGTGTTTCAGCACCCATCAATTTGCCTGTTTG
ATAGGATATGGAGCCAGGTATGACTTTCATTTTTAAAAGATCTGTTTAGTAACAGCTAGA
TCATTACTTATGTTCGCTTCACTTGACATTCTGATCCAGCATAGATGAATATATAAACTC
GCACTCTGTTAGTTTATGAACATGATTATTAAACCAGCATGACTGTTGAATACATACAAT
GCAATCAAGGTGGACTAATGTCATTCATCACTTTTGGTTGCTTATGTCTTTGCATGTCAA
CTTTTTGCAACGTATATTTATGAATTATGATTTCTGTCTTCAGATATCCAATACTATCCT
ATTCCAGGATCGCATAGCAAATCTTCTGGGAGTAGCATCGAAATAAATTATCAACTGAAG
TATTTGATAAAACATCAAACTGAATCAAGTTTTTTCCAGAAGAAATAAATTCCCTTTTCA
AGCGAGTGCACCATAGCCACTTGGCTAGTATTTCCTTCGACAATTGGCGTAGGGCTGAGG
TATAAACGACAATTGTGAATCAGCAGTCGATGTCCTCTGTGGGAACGGAGGGAGTAGAAC
ATAAGACAACTTAAGTCCTCATACTAAGCTGTTAACAAACAATGAGAATACCCTTAAGGC
AGTGCTTCTGTGTGGTTCTTTTCTGTGAAAAAAGGTAGAGGAGGAGCCTTTCTGAAATAT
CTGAATCATCATCCAAAAGAAGTACCGAGTTAGAGGAAGAAAGGATAAAAATAATTCAGT
TAGATGAAGCAGCCACGATAGGTAGAGGAATTGGACTTCGTTCCAGTTTAAATTAGCAAA
TTAGAACCTATTTATTAGGAGGGATTTCTTGAAGATCCGATCATTCCTTTGGTTCTTCAG
CAACTTGTAGTTAGAACTTATATTCAGCTGTGCACATTCTCCATGAACTATTTAGCTCTC
AAGTTACATTGTTGTGTCAGTCGCTAGATTTACTAACATTCATTCTTATGTTGCACATTA
GTGCTATATGCCCATATCTGGCATTGGAAACATGCCGGCAGTGGAGGCTGAGCAACAAAA
CTGTCAATTTGATGCGAAATGGCAAGATGCCCACAGTGACCATCGAGCAGGCTCAAAGAA
ACTTTATCAAGGTATGATTTGGACGGCTTGTAAGTTGTGAGTTTTGACTACTTTGTTGCA
TGTTGTTGTGCTGCTGCCCTTACGTGTGTGAACATAGTTGGGCTCAGTACTACATTTGTG
CATTAATGCTGTAGTCGAGCGTTCAAGTGTGATCTTTGTGTTCCATTTTACATCTGTTAT
TAACTGGTTTATGTTTTTCAGGCAGTAAAATCTGGCCTGCTCAAGATACTCTCAAAAATG
GGCATATCCTTGCTCTCAAGGTTTGTCTTATCTTTGCCATCCAGTGTAATTTAATAATGG
TGCCAGTATGTTGAAGTACTTCTGCAATTGTTTAATGAGTCCACATCTTTTGGATAGTTA
CTGTGGAGCTCAGATCTTTGAAATATATGGTCTTGGCCAAGAAGTTGTCGACCTTGCGTT
CTGTGGGAGTGTATCGAAAATTGGAGGACTCACCCTTAATGAGGTAATTCACAATTATGC
ATATCGTCATGAGTTTTTCAACACCTTTTCTTTTTGGTGTCCATATTCTTGTTGACCTGG
TACATCGTCTGTCTAGAAGAGTGTTTAAATAGATTAACACCTGAGTTGGTTCATGTACCT
TGTTATCTCTTTTGAGACTGTTACCTACTGACAATTCTCCATTTCTTTTATTCTATCCTT
TTCTTGTAAAGCTTGGTCGAGAAACACTATCATTCTGGGTGAGGGCTTTCTCAGAAGATA
CCGCAAAGAGGCTGGAAAACTTCGGATTCATCCAATCCAGACCTGGAGGTAGTATCTCTT
TCATCAGCTGTAACAAAAAATTAGGTTTTGTGAGTTGGGTGCCTGTGTTATGCCAATTTT
AATCCAATATGATTTTTTAATTAAAATGACGTCAATTAACGACCTGAGATCTGGTTGCGG
CAAATTGTACGTGCAATCAACATACAAAGGTCACAACTACTTGAGAAATCTATGTCTCTC
TTGAGTCCTACTTTCGATTCACCAATAGCAAAGTGTCTTCATGGATCCTCTCGTCTCTAG

TTAATTTGTTTTGTTGAAAAGGAGGTGTACTGGAGGGGCCCTTGGTGTTCTGGGTGTTGT
TTTTCAGGGGTGTACGATTGCAATGTTCGGATTTGTCCTAGCCAACCAGGGATAAAAAAT
GCTTACATCTTAACGTGATAGCTAAATCCATTTATCTTTTTTAGCGTTATAGGTACTAAG
GTATGTCCATATAATGAATACTGACAATACCTCCTCCTGACTCCCTAAAGTTACACTTCT
TGTTGCTGAAGGAGTTGCATTTTAATAAATTTGTCGGATCATATGCAATGCAATATTATC
TTACTGTTTATTACGAAATAACATGGTGCCTTTTGCTTCCTGAATAGGTGAATTTCATGC
AAACAATCCTGAGATGTCAAAGCTGCTGCACAAAGCAATTCGTGAAAAAAGTGATAATGC
ATACACCATCTACCAACAACATCTTGCAAGCCGTCCTGTCAATGTTAGTGGTGCTTATCG
TCACTTAGCAGGTGGGCAACCTGTAGATTTTTTCGTTATCACCTTTGGTTTACCATATCT
TCTACGCTGCAGGTTCTTCGAGATCTTGTAGAACTAAAGAGTGAACGGACGCCTATTCCT
ATTGGCAAAGTTGAGCCTGCAACCTCTATCGTTGAGCGTTTTTGCACAGGTGGAATGTCC
TTGGGCGCTATTTCCAGAGAAACACACGAAGCTATTGCAATTGCAATGAATAGGATAGGT
GGGAAATCTAATTCAGGAGAAGGTGGTGAGGTGGGTCCTATAACTATCCATTAGAAAAAT
AAAGCTATTTAGTTATGTCAAGCAGCTCCAGTGGAAACTGCAACATAGAGCTGCTTATGG
ACTTGCTTACTTTCGCAGGACCCAATCCGCTGGAGTCCCCTTACAGATGTTGTTGATGGG
TATTCTGCGACACTTCCTCATCTAAAAGGTCTTCAGAACGGGGACACCGCCACAAGTGCC
ATCAAGCAGGTGCAGCATCAACTGCTTTATTTTTGCACTTTACCTTGGCTATTATATATA
ATTTTGATGCAGATTACGCTTCTATTCTCAAAACCATGACTATGGGTTCCTCTAATTATC
ACTGGGAGATACCATACTCTCATAATGGCTACATTATGAATTCTTCAGTAAATACCGAGT
AGGATTCCACTCAATTTTGTTATGATAACATGGTTAAAAGAGCACTTTGAAAACCAATG
CTCTGAACAACAACATTCTTGCAGATGAACTTGTTTGTAGCCAATTCTCCTTTAGAATAT
GGGGAAATTCACACTGTTGAGTATGCTTGTTTTCCTGTAAAATGGGGCCAGTACGAGTCT
TTTCAGGCCAAGATTACCTTGGGGGCTGAACATAAAAAAATCTTCTATCAGTACATCACTA
GAAGGTGTTTACTGGTTTGCTACTGTAACTGAATGAAATTCGGCACCTGCGCTTCTGCCC
TGGAGGTATCTGTTTCCAGAAGGAATTGGAGGTCTATCAGTGCTAGTTATGTGCGGGGTG
CTATGGTAGTCAGCATTCTCAAATGTGAAACTCTGCAGTGGCAGCATATCCAAATTTTCT
TTATAGATAATGTTTCCTGCTTTTCTGAAAAAGGAAAGAAGTAGAAGCATGTTTTGGTAC
TTTGATTCTGCACTTTAGTATCAAATACGTAGGACGCATATTGTCCACCTCTTTATTTAT
GTGTCCTGAATCAAAGGTACACTATATCCACTATCCAGTGGGTTCATATATTTGAGTTGG
TTCTCATTTGTACTATCCTTTCACAGGTTGCATCTGGACGTTTTGGTGTTACACCAACAT
TTTTAGTTAATGCTGAACAAATTGAGATAAAGATTGCACAAGGGGCTAAGCCTGGTGAAG
GGGGTCAACTTCCTGGGAAAAAAGTCAGTGCATACATAGCTCGGTTGAGAAACTCCAAAC
CTGGTGTTCCTCTCATATCCCCACCACCACACCATGACATCTATTCTATTGAGGATCTAG
CGCAGCTAATTTTTGACCTACATCAGGTTTGTTTTGCACAACTACTTTTACAATATCAAC
AAGTAATGTTTGTGCTAAGGGAAACTGCTAGCTTTAAAATAAGTTTTATGTATGTCACAT
AAGAATTAGAGCCCTTCAGCTGCCTATATGTTAGCTTCAAAATTAATTATGTGCGTCACA
TAAGAATTAGAGTCCTTAAGCTGCCTATGTGTCAATTAGCAATGCTGTTTAATTATTGAT
TTCTTTGAGAAGCAAATCCATTTATTGGTACTAATCAAATATTTAATTCATATTTGACTG
GGCAGATCAATCCTAAAGCGAAGGTGTCGGTAAAGCTTGTAGCTGAAGCAGGAATTGGAA
CTGTAGCCTCTGGGGTATCTAAGGCCAATGCAGACGTAATTCAGGTAGATTAAACATCAA
AATTTTAATAAGTCATTGATTTAGCTCATACCATTATTGTACGTGGATCTGTATCCCGTA
AGTGGCCATGCTTCTGTGGTTTAGTGTTATTTTACATTTTTTTGTATCCTTAACCTTTTG
CTGTAAGGTGCTACTCCCTCCGTCCGAAATTACTTGTCATCAAAATCGATAAAAAGAGAT
GTATCTAGAACTAAAATACGTCTAAATACATCCCCTTTTATCCATTTTGATGACAAGTAT
TTCCGGACAGAGGGAGTATCTGTTAAGTTGGCCCTCATGAGGCTGCGTAAACCGCCTTTT
GCTATTTCATGCTGCAGATAGTGACATTTGCTGTCTGATGTTCACGATGGTAGACCTTCC
TATTATAGTTCATTTTTGTATGAAAAATATGAATTTCTCRCTCCCTGTGAATAGCCAGTT
GTTAGACATTGCTATAGTGGCACTTGTCCTGTAACAGACAGACATCACTTTTTAGTGAAT
TATATGCAATAATCTGTCAGAGTTTTTTTGGGGAAAAATCCCAGAAATCTAGTAAAAATT
CCCAGAACACAAAAAATTCCAGAAATATAGAAATGTTTATTGCACACACAGACACCAATAA
GGTAAGCAATGACTTATGAAGATGCCGATTTAGTTTAATGAATTGGATGAGCACACTAAA
CCTGTTGGTTGTTACATCATTTGAGTCTCTTGAGAGAAACCATGCTATGGAATCTTATTT
AATTTGTTGCGAGTGGTTTCATCCAAAGTCTAATGGGTGAACTAGAGGTTTAGTATACAT
ATTTTGGTGATCTAATATGCCATTTTAATATACAGATATCAGGTCATGATGGTGGTACTG
GAGCTAGCCCAATTAGCTCAATCAAGCATGCTGGGGGTCCTTGGGAACTTGGTCTTACGG
AAACACACCAGGTTTTCTACATTTAATTGCTTGATATACTCAGTTTCTTTTAATATCAAA
TCACTTTCTTTGTTCTAAACCTAGTTAGCTTCCTTCAGACACTCATACAAAATGGATTGA
GAGAGAGGGTGGTACTTAGGGTGGATGGCGGATTCAGGAGTGGCCTAGATGTCCTTTTGG
CTGCTGCCATGGGTGCTGATGAATATGGCTTTGGTTCTGTAGCTATGATAGCTACCGGAT
GTGTCATGGCACGCATTTGCCACACAAACAATTGCCCAGTTGGAGTTGCTAGTCAGGTAG

CAACAGCAGAATTGATATTTTTGTTTGTCTTGCTTGGTTATACCTTACATTCTATGCTCC
TGCAGAGAGAAGAGCTACGCGCTAGGTTCCCTGGTGTTCCTGGTGATCTTGTGAATTACT
TTCTCTTTGTTGCAGAGGAGGTATGGCATCACTCACATTACTGGTGTCTTTTCTGTTAAC
TTCTTTCTGTGCTTGATTTGCAAATTTGGTCATGCATAATTTGGGAGTAACCATGACTCA
TATTTCAAGTACTCATGCTTCTGTTCCAGGTACGAGCCACATTAGCCCAGTTAGGTTATG
AGAAACTGGATGACATAATTGGGCGGACAGATTTACTTAAGCCAAAGCATATCTCTTTGG
TGAAAACGCAGCACATTGATCTTGCATACCTATTAATGGTATGGAGTTGAGCTATTCTCA
TTTGAGTTATTGGCATTTCTTGCCTATCTTGTTATTATATTTATCAGCGTTCTCGTTTCA
GAATGCTGGATTACCCAAATGGAGCAGCTCTCAGATAAGGAGCCAGGACGTCCACTCTAA
TGGCCCTGTTCTTGACGAGACAATCCTTGCAGATCCTGAGGTAATGTTTTTGATAGCTTT
TCTTAGCTACATCGATTAACAACTGTCCTCCTGAGCAGATTTGTGTACACCTAGTAATTT
TTTTGATCGGGAACAAAGAACTGTGACGTTGATCAATAGATATTGTGGCCTAAAGTGAAA
TGCATAGTTGCTAGGAACCAAGCTCTGCTGTATGCAGCATTACTTAAATTATATAATTGC
ATTTTGCAGGTATCCGATGCGATTGAGAACGAGAAAGAGGTTTCAAAGACATATCCAATT
TATAATGTTGATAGGGCTGTTTGCGGCCGTGTAGCGGGTGCCATTGCTAAGAAGTATGGA
GATACAGGCTTTGCAGGCCAGCTGAACATTACGTATGTCAACTACTTTCTTGTCTGCATA
ATTCTGTGCCCTTTAACTTTTATTATGAGAAGATAGGAAATGAACAATTTCAAACATTTC
AGGTTCACCGGAAGTGCAGGACAGTCCTTTGGTTGTTTTTTGACACCTGGCATGAATGTT
CGCCTAGTAGGAGAGGCCAACGATTATGTGGGAAAGGTACTAAATGCTCAACTGGACTGA
CAATACTGTCTGACCAGCAGCTTTATAATGCCTTCTATTTCATTTTCACAGGGTATGGCG
GGTGGAGAGCTGGTTGTAGTTCCTGTAGATGATACAGGATTTGTTCCTGAGGATGCTGCT
ATAGTTGGGAACACTTGTCTGTATGGAGCTACGGGTGGTCAAGTATTTGTGAGAGGCAAG
ACAGGAGAAAGATTTGCAGTTCGGAATTCTCTTGGGCAAGCAGTGGTTGAGGGCACAGGA
GATCATTGCTGCGAATACATGACTGGTGGATGCGTAGTTGTACTTGGCAAGTAAGTACAC
AGTCTGCATTTTTCCCACTTTGTGTATTGCCCTTCTTNNNNNNNNNNNNNNNNNNNNNNN
NNNNNNAGTTGGAAGAAACGTTGCAGCCGGAATGACTGGTGGCCTGGCCTACATGCTAGA
TGAAGATGATACACTAGTCCCGAAGGTACGCAAATTTGGAATCTGTAGCTATACCTTAAG
CTGCCCCGTAGCATGCATCTAGTGAAAGCCTTTTGGATTCACATCTCAAGATCATAACGT
GCAAGTGTTTTAATAGGTAAACAAGGAGATTGTCAAGATGCAGAGAGTGAATGCTCCAGC
TGGGCAGATGCAGCTCAAGGGCTTGATTGAGGCCTATGTTGTAAGTCTAGTGCACTCTTG
GAAAATTTTGCGCCCAAATTGGCCACTCCCCATGTTCTTATAAATTGGATGTCGCTTTTA
CAGGAAAAAAACGGGCAGCACGAAAGGTGCCAAAATTCTGAACGAATGGGAGGCATATCTG
CCACTGTTCTGGCAGTTGGTGCCACCCAGCGAAGAAGACTCGCCTGAAGCTTGTGCTGAG
TTTGAGAGAGTACTTGCCAGGCAAAAAACAGCAGTACAATCTGCCAAGTGATACTAGCCG
AAGCCCAATCGTCAAAATGATCAAAGCTAATCGGAGGTTAAGCGTTCTTACTGCGATACT
TGAAAGTGATTTTCTCAGACCGCGGTCCTTCTAAATCACTAACGTCACCAAAGGCTGCAA
TTTTGTGGAAGCTCTGTCCAAGGCATCTTCTGGAAACGAGCTTGCAGATTCCGTCGAAGA
TTCTTCTGCACGGTCTTGCAGCCTAGGTAACCAACCACCCCTGTAAATTGGTTCGCTGAC
GCTTGGCTGCAAGGCCCGGCCCCTGTTGAACTGCTGCTTTCTTGTACAGGATTCCTGGCT
TCGCCTGTGTAATTGTATTTTTTGTTCTCGACACATTAGTTCTTATATTTATACCCGTCC
TGTGGTAGGCATGCGAGTTGCATGTGCTTTAGTTCAGTATACAGAAATAAATAAAGTGTA
TGATGAAGTAGGACTTCTCAGGTGTCGATAATCGGTGGAAATTGTTTTTCTTTTTTCAAG
TTCTACGGGGCGTCGGCCAACTTCAACGATCTCCATTATTAACTCGATGGCGCCAGCGTC
ACCGAGGTTTGAGGTTTGACGGCGTGTGAGAAGAGAGCCGGAAAGCTCTTACTCTCTTGG
GTCCTACAACGGGGGAAAATATTGGTTGACATGCATCAAACATGTAGGAATCTTCTATAT
CTAAATAGCTAGGCCCCACTAACTATTTTTCTTAACATGCAAACATGCCACATCATTGCA
CAACATGCATGAGAAAAGGCCCACCCACTACCATATATCTCTCAGAATGGAAGCATGACA
CTTCATCATGTATATGCATGAAAAGAAGCCCAATCTAAACATGCAAACATACCTGAAAAG
AGCCAACAAAAGAAGGAAAAAAATAAGCCAAATACTTACATATAACTGGTGAGTTTTGGC
TTTGAATATGAAAATAGCCACCAAATAAGTAAACCCTTATAAAAGCCCAAATGGTAATTG
CCACACGTGTGACACGAAGCACTCCGGTGTTTTTAACAGCTAAAATTGGCATCCGAAAAG
AAAAATCAAACACAAAAATAACTAAAACCTATCACCCGAAATAAGTTGCCATGCTTAACA
ACTAATCTTGCCATCCTCGCATCACTAACTTGCCATAAAACGTTTGGAGTTGCCATGTGT
TCGTGCCACACATGTGGCACTTATCAGAGTCCTATTGTCATGATAAAATATAATGAACAT
GGTTTGCATAACACACACACCGTCAACAACGACAGGTTTCTAATACTCCCTCCGTTCCAA
A

>Chinese Spring D-genome
CGCACCGGCCGGGCGCCCTNAGTAAGCCCGCNCAGGAGGTATGGTTACGCTCTGCTCCCC
ACCCGCCGATCAAGTGCTCGTCTTCGCGTATTGGGTTTGGTTTCGGGGGTTGGGGTTTTT

GTAGGTTTGGATTGTTTGGTGCGGTTGCTAGATTGGTTTATTTACGAAGTCACGGGCCAT
GGCTTGTTTTGACGGATCATAGTTTATTTATGTATTTGCACTGTAATATGCTTCTGATAG
CTATACGGTTGCCAAATTTCTGCGTTGGGAAATTTGGGGGAGATCAGGATTCAGGAGACC
CAGGTATCTCCTGATTACTGTATCGGACGATAAGGTTCTATGTGTTGACTCCAAAGTTCA
TTGCATGTGCTTGTTTTATTAGGTCTCTAGCTCATGATGATGCACTTGCATACTATCGCT
TTTGTTATGTGGTGTTCCTTTCATGGGATTTTTAAAGTACAAAGCGCAGCAAGTGCACAA
AACACAGCAAATGCGGGTGCAATAAACAAGTGTGTGGTTATGAAAGGTTAGGTGACAGCT
GTGGTGGTAGGTACTAATCACGATCAAGTTCCAGGAGTACGACATTGTGTAATGCATAGG
TGGAGAATTTGCAACGTACTAGTACTAGTTTTCCATTTCCGTTCTGGAATGCGTTTGAAT
GGGCTGTGACCTGTGAGCTGTGACAGTTATGAGTAATAATAAACCATATGTGCTGTGCTT
TATATCTGACATTAGGAATTGCTTTTTTCTGTCAGGCTGCGGATTTGAACGATATCTTAG
CAGAACGTGGAGCCTGTGGTGTTGGGTTTGTCGCAAACTTGAGTAATGAGCCTTCGTTCA
ACGTTGTCCGTGATGCTCTTACAGCTCTTGGGTGTATGGAGCACCGTGGTGGCTGTGGAT
CCGACAACGACTCTGGTGATGGGGCAGGATTGATGAGCGGTATACCATGGGACTTGTTTG
ATGACTGGGCCAGCAAGGAAGGGCTTGCTCCTTTTGAAAGAACACATACAGGTGTAGGCA
TGGTCTTCCTTCCACAAAACGAGAATTCTATGGCAGAAGCAAAAGCTGGTAATGATTCTG
ATGCTTAACCACCATACGTTGTCCCCATTGTGGATCATTTCTTGTCTTTCTCTTTTGCTG
GGATCTCATCTTTAAAGTACGACACTTCCTAATTTTATATGGTTAGAGATTTCTGCTTGG
CGATGCATGTCCTAGTAAATCTGACTCCGATCTCTTCGGACTTGTAATGCTGCCATACTT
TGGTTGCCATGCCATTGTGGTGTTTGAAACCTACGGTTGGAAAGTTCTTTTGCAGGAAAA
GTTTATGCCCTTGGTATAAGAACTTTCCGAAAGGAAATTTATTATTTTGTATTGACAGTC
AGATTACACATGTGTTTTTTCAGCTCCACTGTACCACAACACACATAGCTTCCTAAGAGT
CTATTCATTTTGCAGCTGTTGAGAAGGTTTTTACAGATGAAGGCCTTGAGGTTCTTGGCT
GGAGACCGGTTCCTTTCAATCTATCAGTGGTAGGCCGCAATGCAAAAGAAACAATGCCTA
ATATACTCCAGATATTTGTGAGAATTGCGAAAGAAGATGACGCTGATGACATAGAGAGAG
AATTATACATCTGCCGAAAGCTGATAGAGAGGGCTACAAAATCTGCTAGTTGGGCAGATG
AACTATATTTCTGCTCTTTGTCAAGTAGAACTATCATTTACAAGGGAATGCTTCGATCTG
AGGTTCTTGGGCAGTTCTATTTGGACCTTAAGAATGAACTGTACAAATCTCCTTTTGCCA
TATACCATCGAAGATTCAGTACCAACACAAGCCCTAGATGGCCTCTTGCACAACCAATGA
GGTTGCTTGGACACAATGGAGAGATTAACACGATACAGGTTCACATGAACTTCTTCAACT
TACTGTTAATTTTTACTCCCTGTATCTTACATTATTGTTTAGTGTCTTCCTTGTTGCTCG
CCAAATGTTTGATACTGAAAACTAGGCATACTTTGGGCATTACTCATAGCGTTCCTTTTC
TTTGCAGGGAAACTTGAACTGGATGCGATCAAGGGAAGCCACAATACAGTCTCCTGTATG
GCGAGGCCGTGAGAATGAACTACGCCCATTTGGTGACCCTAAAGCATCCGATTCAGCAAA
CCTTGACAGTGCTGCTGAAGTACGAGACTGCACACCGTTCATTAATATCGTAACATAAAG
CATGCATTTATTTTGCTTCTTGTCCATACAACCGCGCCATTCACACGATTTTGCATACTT
GTCACTATAGAATATTCCAAGCCCTTCTGGGCTCTGTTGAACATGCTTAATGACACATAA
CTCAGACATCTGTCAAGATGTTACAAGAGATTAACAATTTGGCTTTTAATGGGTACTGAA
TAAGCTGTTACTTTAATTTGACTATTTTCTGCACCTTGATTTACAGAGATGAAATGAACC
AAATTAAAGTAGTTTCATAAATTTCAAACATACGTGCAACATTTATGTTATTTTGAAGCA
AAGCTTTTGAAATTTTGGATAGCGAAGTGGTACTTTGACTACTCGAATTAATGCTAGAAA
CATATGCAGTTGTTGCTAAGAGGCAATCTGACCTATCTGTTTTCACTGTGCATAACAAAT
CATATATGCTATTCTTAATGTGCATTGAGCACCCCCGCCTTTATTTCGAGTATGTATTTC
TACCTTCATTACTGGTGTGTAATTGTTGAGTCATCACATCCTTTCTTGCACCAGTTACTG
TTAAGAAGTGGTAGAAGTCCTGCTGAAGCTATGATGATGCTCGTCCCCGAGGCATACAAG
AACCATCCGACATTATCAGTAAAATATCCAGAGGTATGGGATGCATTAGTGAACATATGT
TGTGGGCATGCATGAAGTCCCTTCCATATCAACTTATGCAAACAATTGTGTCCAATTGAA
GCTTCTCTTGGTTTGTGTCTTAGGTAATTGACTTCTATGAATACTACAAAGGTCAAATGG
AGGCTTGGGATGGGCCTGCTTTACTTTTGTTTAGGTAATATCTCGTCGATCTGTGATTTT
GCCGTTTTATATTTTTTGCTATCTTTATTTTCTGATGTAAAGACAGTTTCTTTACTAGTT
TGGTTAAAGTCATTTCATGCATTAGCAGGTTTTGCTTCCTTCTCACTTTGTCCCTCTTTT
TCTGAATAGCCGTCATTTAGAAGTTTTGCAAAACAACCAAGGCACATATAGGAAAGACCA
CATTACTCCCTTTCATGGGGTTACTTGTTATTCTTGGGATGGTATGTTAGTCTACTCCAT
TGTAATAAGTGACATCAGAAATGGGCTAATGTTAGGGGAATACAAGAAAACAATGGCCCA
AACTTATCTTGGAGTCATAGCGAGTTTTTACGTATGGCCTTTATTTAACACATTCACAGT
TTGACCTGTAATTAGGAACCAAATTTGTGTGCTTATGGCATGTATTGGGGAAGGGAGGTA
GTATATACCTAACTGCAGGAACTAAAAGATACAGCAAATAGGTGTTTAATTGCAAAGAGA
GAAAATCAGAACACAGAGATGGGTATGTGCTAGCATCACAAGTTGTGGACAATATTTAGT
TGTAGAAGTTGAAACCTTTGACAGAGAAGCTTCCAAACATGTATTTGTCGTCAAACAGAG
TAGGGAAAATAGCCGTAATTTTTCCTCCGTGCCGTGTTATTTAAAGCATAAGATATCCAA

ATGCTGCTTTTTCTCTACTGAGTAATTTCTTGCAACAGAGTGGGCTAGATTTACATGTTC
TCTTTTGTATACCTCTATGTAGTGACGGAAGGACGGTAGGGGCATGCCTTGATCGAAATG
GGCTGCGTCCAGCACGCTATTGGAAAACATCAGATGGTTTTGTTTATGTTGCATCTGAGG
TAGGTTCAAGTTACTTCTACTGTCTGAAATTTCTGTTTAGTTACAATGTTATCGTAGAGC
GATTTTGTATCACACATTAGTGAAGTGTCCATTTTGAAATTCTGAAATTGGAGCATTTGG
TTTATCTGCCAATAACTTATTGCAGATATACTGACATCCTTCTATACCAAATCGCAGAGT
TCAGATGTCACAAGCTAAACTTAATGTAATATTCAGTAAGTATTGTGCCTATTTGTTGAA
GTGTTAGGTCTCACAGCTACACTAGGTGATTAGGACAAATTAAGAAGTTACTTGTTAGTG
TTTGATTGCTATTTGAATGCTATGTCGCTCATGCATTTGTGCCAGCAAGCTTCAAGGATC
CTACATGGGTTTTTGTTCTTGCCTTTGTGGGTACAAATTCATTCTACGGTGACAGATCGT
GTAAACCTTTGTTTGAGTAGAGTTAGATATATCAATCTGTTTTTTGTCGTTSCAAGACTT
GACTTGGATGTAGGCATATAGCAATAACTTGACATTTTAACATATTTTTGGGCTACACTT
CTCAGCTTGTTCTCTCATATTGTGCAGTACTTTCATCCTACTAGAGAGTACACAGTACCA
AGATTGGAAGTTATTTTTTTATGCTCGCTTTGTCTTGAATAAACTTCCCTCACATGAGAT
CCTTTGTTCTGCTGATGCCTATACTCAGAGAGTAAAAGATAAAACTGTTAGTTACCATAC
ATGATGCTTCTTATGGTTGTAGTTTGACTAGTCTAGCTTATTAGATGTGAATTTATACTC
TAGTAATACCTTTGACCATCTCTTGTGTAGTTGAATCTGATCTGATTATTTTAAAAGCAT
GTCATTTAACTATATGCTAATAGTTGACGAAGTAACCTTGGATCACATTCTCAAATGTTT
GTTTTTTGTTGTTGCGTGGAAACATTCTCAAATGTTCTACAAGAGTTTGGCAAGTATTAC
TTTGTAGGATTCTCACCAACTGACTAGCTGTATTAATGAAAATCTACAGGTTGGTGTTAT
ACCGATGGATGAGTCGAAGGTAGTAATGAAAGGAAGATTGGGTCCTGGAATGATGATAAC
AGTTGACCTAGAGACTGGTCAGGTTAGAATAGAATAAAACAATCTTTTCCTTGTCTCGCC
TTTGATCATCAGAGTCTTCTAAAAATGTAGATTCTAAACCTCACGAGTCATCCCAGGTCC
TTGAAAACACAGAAGTGAAGAAGAATGTGGCTTCAGCAAAACCCTATGGAACTTGGTTGC
AAGAAAGTACACGTTCAATAAAGCCTGTCAACTTCCAATCCTCTCCTGTCATGGACAATG
AAACAATTTTGAGACATCAGCAGTAAGAACCATGAAATATTGCTCTATTGAACTCCTCTT
TACTTTTCCCCTTGTACTTATTTGCTTATGTTTGTTCCAAGAGTTAGAATGTATGTATGT
ATGTCTAGCAATATGCCTGATGCTTATGTTGAGAAGGCTGTGGACTTGTGGCTGACTATT
TCAGTGATGGTATATTGCAATGCCTTTTGCTCTTTCTGGGTTATGTAAACCTTTTTAAAC
TATATACATAAAATCATTTGGGTCAACATATTGTGTCCATCAAAAAGATTTCCACAGATT
ATATTATGGGATGTCTGAAGTAAACCTAGCTTGATACAGAAGTATAGGGCTATTAGTTTT
TTTTTCAGATTTCAGAAGATAAACCCCAAAAATGTTACTTATGATCATGTCATGCCCAGA
AATATACTGCCAAAGAATGTGGCTGATTTTCACGTGTGCTCCATGTCTACAACTTACCTA
TGTTGTTGTTTCAGACATTGTAAGAATTAGAGATATTAGATGGTCAGTCAGATTTGATGT
TTAATTTAATGTTATTATATTTTTAGGGCATTTGGTTATTCCAGTGAAGATGTGCAAATG
GTAATTGAAACAATGGCTTCACAAGGGAAGGAGCCAACATTTTGCATGGGCGATGACATT
CCATTAGCCGTGTTGTCACAAAAGCCACACATGCTCTTTGATTATTTCAAGCAGCGATTT
GCACAGGTCAGTAAATCCTTAACATTTCAGTAATAGTGTCAAAAAATCTAGGTTATGAAA
CTTCCTCTATTACTCTTAAATACTTGCACGATATTGGCAGTCCTTGCCAAAAAATTCTTA
TCCACAGTACAGTTAGTTGGGAAAATCACATCTGCGTAGTCACATTCCAAGGTTATTTTG
GAGTAACTAATTCCATCCAATCTTCAATCCATTCAGTTATAGGATGACTTGATTTGACCC
CTAAGAAATAATCTCCATCTTCATAGAAACATACCTTAGACGGTGTATATCTATATGGAG
GTGTAAATCTAAGTACAACATATGATACTATCAATAAGGAAGTAAACCGAACAAACAAGG
GAGCTAATTAGTGTGATCTCTAATTGCCAACTAATACCTACAAAGCATGGATAYGGCAGA
AACTGCCGAATTGCCGTCCTAGTACGGGGGGATACGGGGACACGCGCGGATACGCCGGGA
TACACGTATCCTGCAGTATCCCATCTTTTCTGAATTAAAAAAAGGGGAAGAAATCGGGAT
ACGGCAGATAAGGTGTGGATACGGTGGATAAGGCTGCAGCTGCCTGGACTCGCTTCTCTG
GTCGCTGGCTCGCCGCCGCCGTTTCCCCCAAGCCCAGCTTTCCCCCCGACGAAGAACATC
CATCAAGCAGAGAGGAAGATGGCAAGGTGTTCGAGGACAAGGAAGAGGCACCGGGCGGGG
AGAAAGATGGGGACTGCAGCCAGCAGAAAGGAAGAAGATATAAACGGCGGCGCAGTCTTT
TCTTCTTCTGCTCTCTGCAGCTCTCAGGCGGCTGGAGGAAACGAGAAGAGAACGGCGAGA
GGAGCTGACTAGTCTAGGGTTTCAGGAACTGGACCTTATATGGGCTCTGAGTGGAGAGCT
ACATGGGCCTGAGTGGAGGGTTCGTATGGACTATGGGCTGCCCCAAAGTCTTTTATCAAT
AGAAATGAGGCAACATATATCCAGTAATTCCTTTACAAAAATCATATATCCAGTACAAAA
TCTCTGATCTAAAAATTATCTTTAAATCTTTATTACATGCTAGTATTTTTTCTATATATT
TATATACCCCGCCGTATCCCCGAATGACTGTTTTTGAAAAATGCTGTATCCCCGTCTTTC
CGTCCCCGTGTCCGTGCTTTTTAGACTAATACTGCACCAGCTGGGCCTGCATGAACAGTG
TGCAGGCGCACCGTGGCTGCACAGCAGTAACGGGTCCTCATTTACAAATTATCTTATTAA
TCGAAGTCATGACTGCTTTGTCTTATGATTGTATCTCATTCAATTTCTAGGTTACAAATC
CTGCAATTGATCCACTCAGAGAAGGTTTAGTCATGTCTCTTGAAGTTAACATTGGTAAAC

GAGGCAACATCTTGGAGGTTGGCCCTGAAAATGCTGACCAGGTTAGTTAGTTTTTAAGGT
AATGATTTGACCTGTATTCTTGGGGCAATATTTTGATATCATCTTATTGACTTCTCAGGT
TACCCTATCAAGTCCTGTGCTGAATGAAGGTGAACTAGAATCACTATTGAAGGACCCAAA
ATTAAAGCCCAAAGTACTCTCGACATACTTCAATATCCGTAAAGGTCTAGATGGTTCCCT
TGAGAACGCAATTAAGGCTCTTTGTGAAGAAGCTGATGCTGCTGTGCGGAGTGGCTCTCA
ACTTCTTGTGCTTTCCGATCGTTCTGAAGCGCTTGTGAGTAGCTTCTGACTTCTTAATGT
GTTAACACAGATATCCACAGCAATCTTTATGCTCCTTGTTTATAGACTCGAACTATATTT
CAAGTAGCTTTCATATTCTTGTTCACCTTTCTTTTTCAATGGGTAGGGAAGCAGTGAATG
TGCTTGGTTTGAACTTAAACTATTTTTTACTTCGCTGCATAATGGGCAATACTGATTTCT
AGAAGTGTACCAGATCCAAATACATGTATACTTACTATCTACATCTATGGTGCACCAGTA
CAGTACTACAAAATGATCTACTGGTTCCTTGACAAGTGTCAGTTAGTTACTATTCATGGG
CTACCTTTGTCTAATTATGCATCCACCAGGAGCACTATTCACCATATAGGATAGTACCAA
TCTATATAATTTGAAGGGCTGGACTACAGCGGCGCTTAACGCGCGACCTGTCCTAGTTCG
GACTTTTGGTTGCGTTGGCTAGTGCATGAATCTTGACATGGTATCAGAACCTAAGGTCTC
GAGTTCAAGTCCTGTCTTTTGCAGTTTATCCAAAAATTAACCTAAGAAATTTAATTTATA
CCAAACTGGACACTTGGATATCACAATGTCTTTATTGTCACAATTTAGTACACATAATTG
TGCAAAATAATATAAGACACCCGCGCATCAATATGATATGCCTATAATGATTGATGCATG
TGGATGTACATGGTAGCTTCTTGTCTATGACATTGCTTGCCATATCTAGTACTAATATGA
TTCCACTTCTAAACTATTTGGTCTTGTGACCAGAAAATACAGACATTACTTTTCCTGACA
TACAGTTACTTGAAAACATCACGATGTAGTGCTAGGTTATGAAGCCATTATTTTTTTGTC
AACTTCAGAATCTCTCTTCTGCAATGCAATACACTACGGAGATAGAATCATCTATTTTTT
TACTACACTTCCTGTATTGCAATTAAATGAATGTATGATGTAGGAACCAACACGGCCTGC
CGTCCCAATACTTTTAGCTGTTGGTGCCATCCATCAGCATTTGATTCAGAATGGCCTCCG
CATGTCAGCTTCAATTGTTGCTGACACTGCCCAGTGTTTCAGCACCCATCAATTTGCCTG
TTTGATAGGATATGGAGCCAGGTATGACTTTCATTTTAAAAAGGATTTGTTTAGTAACA
GCTAGATCATTACTTATGTTCGCTTCACTTGACATTCTGATCCAGCATAGATGAATATAT
AAACTCCCACTGTGTTAGTTTATGAACATGATTATTAAACCAGCATGGCTGTTGAATACA
TACAATACAATCAAGGTGAACTAATGTCATTCAGCACTTTTGGTTGCTTATGTCTTCGCA
TGTCAACTTTTTGCAATATATATTTATGATTTCTGTCTTCAGATATCCAATACTACTACC
TCTATCCATCTATATAGGGCCTAATGCGTTTTTCAAGGCTAACTTTGACCACATGTTATA
GCAATAATATATGACATGCAAGTTACACAAAGCATACCATCAAATTCGTACGTGAGAGGA
GCATCCAATGGTATAATTTTTCACATTATACATCTCMATATACTATTAATCTTGTCAATA
GTCAAAAGCAATCTCGAAAAACGCATTTGGCCCTATATATATGGATGGATGGAGTATCTT
ATTCCAGGATCACATAGCAAATCTTCTGGGAGTAGCATCGAAATAAATTATCAACTGAAG
TATTTGATAAAACATCAAACTGATTCAAGTTTTTTCCAGAAGAAACAAATTCCCTTTTCA
AGCTAGTGCACCATAGCCATTTGGCAAGTATTTCCTTCGACAATTGGCGTAGAACTGAGG
TATAAACGATAATTGTGAATCAGCAGTCGATATCCTCTGGAAACTGGGCATGCAGTTTGG
ACTTGCAGAATGAATTAGAACATAAGACAACTTAAGTCCTCATACTAAGCTGTTAACAAA
CAATGTGAATACCCTTAAGAGAGTTCTTCTGTGTGGTTCTTTTCTGTGAAAAATGGTAGA
GGAGGAGCCTTACTGAAATATGAATCATCATCCAAAAGAAGTACCAAGTTAAGAGGAAGA
AAGGATAAAAATAATTCAGTTAGATGAAGCAGATAGGAGGAATTGGACTTCGTTCCAGTT
TAAATTAGCAAATTAGAACCTATTTATTGGGAGGGATTTCTTGAAGATCCAATCATTCCT
TTGGTTCTCAGCAACTTGTAGTTAGAACTTATATTCAGCTGTGCAAATTCTCCAGGAACT
ATTTAGTTCTCAAGTTACAATGTTGTGTCAGTCGCTAGATTTACTAACATTCATTCTTAT
GTTGCACATTAGTGCTATATGCCCATATCTGGCATTGGAAACATGCCGGCAATGGAGGCT
GAGCAACAAAACTGTCAATTTGATGAGAAATGGCAAGATGCCCACAGTGACCATCGAGCA
GGCTCAAAGAAACTTTATCAAGGTATGATTTCTATGGCTTGTAAGTTGTGAGTTTTGACT
AGTTTGTTGCATGTTGTTGTGCTGCTGCCCCTACGTGTGTGAACATAGTTGGACTCAGTA
CTACATTTGTGCATTAATGCTGTAGTCGATCGTTCAAGTGTGATCTTTGTGTTCCATTTT
ACATCTGTTATTAATTGGTTTATGTTTTTCAGGCAGTAAAATCTGGCCTGCTCAAGATAC
TCTCAAAAAATGGGCATATCCTTGCTCTCAAGGTTTGTCTTATCTTTGCCATCCAGTGTAA
TTTAATAATGGTGCCAGTATGCTGAAGTACTTCCTGCAATTGTTTAATGGGTCCACATCT
TTTGGATAGTTACTGTGGAGCTCAGATCTTTGAAATATATGGTCTTGGCCAAGAAGTTGT
CGACCTTGCGTTCTGTGGGAGTGTATCGAAAATTGGAGGACTCACCCTTAATGAGGTAAT
TCACAATTATGCATATAGTCATGAGTTTTTCAACACCTTTCTTTTTGGTTTTGATATTCT
TGTTGACCTGGTACATCGTCTGTCTAGAAGAGTGTTTGAATAGATTAACACCTGAGTTGC
TTCGTGTACCTTTGTTATCTCTTTTGAGACTGTTACCTACTGACAATTCTCCATTTCTTT
TATTCTATCCTTTTCTTGTAAAGCTGGGCCGAGAAACACTATCATTCTGGGTGAGGGCTT
TCTCAGAAGATACCGCAAAGAGGCTGGAAAACTTCGGATTCATCCAATCCAGACCTGGAG
GTAGTATCTCTTTCATCAGCTGTAACAAAAAGTTAGGTTTTGTGAGTTGGGTGCCTGTGT

TCTGCCAATTTTAATCCAATATGATTTTTTAATTAAAATGACATCAATTAACGACCTGAG
ATCTGGTTGCGGCAAATTGTACGTGCAATCAACACATACAAAGGTCACAACTACTTGAGA
AATCTATGTTCCTGTGAGTCCTACTTTTGGTTCACCAATAGCAAAGTGTCTTCATGGATC
CTGTCGTCTCTAGTTAATTTGTTTTGTTGAAAAGGTGGTGTACTGGAGGGGCCATTGGTG
TTCTGGGTGTTGTTTTTCAGGGGTGCACGATTGCAAATGTTCGGATTTGTCCTAGCCAAC
CAGGGATAAAAAATGCTTACATCTTAACGTGATAGCTAAATCCATTTACCTTTTTAGCG
TGATAGGTACTAAAGGTATGTCCATATAATGAATACTGACAATACCTCCTGATTCTTCCC
TAAAACACTTCTTGTTGCTGAAGGAGTTGCATTTTAATAAATTTGTCAGATCATATGCAG
TGCAATATTATCTTACTGTTTATTACTGAAATAACATGGTGCCTTTTGCTTCCTGAATAG
GTGAATTTCATGCAAATAATCCTGAGATGTCAAAGCTGCTGCACAAAGCAATTCGTGAAA
AAAGTGATAATGCATATACCATCTACCAACAACATCTTGCAAGCCGTCCTGTCAATGTTA
GTGGTGCTTATCTTCACTTAGCAGGTGGGCAACCTGTAGAATTTTTTGTTATCACCTTTG
GTTTACCATATCTTCTACGCTGCAGGTTCTTCGAGATCTTGTAGAACTAAAGAGTGAACG
GACGCCTATTCCTATTGGCAAAGTTGAGCCTGCAACCTCTATCGTTGAGCGTTTTTGCAC
AGGTGGAATGTCCTTGGGCGCTATTTCCAGAGAAACACACGAAGCTATTGCAATTGCAAT
GAATAGGATAGGTGGGAAATCTAATTCAGGAGAAGGTGGTGAGGTGGGTCCTATAACTAT
CCATTAGAAGAATAAAGCTATTTAGTTATGTCAAGCGGCTCCAGTGGAAACTGCAACATA
GAGCTGCTTATGGATTTGCTTACTTTTGCAGGACCCAATCCGCTGGAGTCCCCTTACAGA
TGTTGTTGATGGGTATTCTTCGACACTTCCTCATCTAAAAGGTCTTCAGAACGGGGACAC
CGCCACAAGTGCCATTAAGCAGGTGCAGCATCAACTGCTTTATTTTTGCACTTTACCTTG
GCTATTATATATATAATTTTGAGTTGGTTCATATATTTGAGTTGGTTCTCATATGTACTATC
CTTTCACAGGTTGCATCTGGACGTTTTGGTGTTACACCAACATTTTTAGTTAATGCCGAA
CAAATTGAGATAAAGATTGCACAAGGGGCTAAGCCTGGTGAAGGGGGTCAACTTCCTGGC
AAAAAAGTCAGCGCATACATAGCTCGGTTGAGAAACTCCAAACCTGGTGTTCCTCTGATA
TCCCCACCACCACACCATGACATCTATTCTATTGAGGATCTGGCGCAGCTAATTTTTGAC
CTACATCAGGTTTGTTTTGCACAACTACTTATACAATATCAACTAGTAATGTTGGTGCTA
AGGGAAAACTGCTAGCTTTAAAATAAGTTTTATGTATGTCACATAAGAATTAGAGTCCTT
CAGCTGCCTATATGTTAGCTTCAAAATAAATTATGCGTGTCACATAAGAATTAGAGTCCT
TAAGCTGCCTATATGTCAACTAGCAGTGCTGTTTAATTATTGATTTCTTTGAGAAGGAAA
TCCATTTATTGGTACTAATCAAATATTTAATTCATATTTGACTGGGCAGATCAATCCTAA
AGCGAAGGTGTCGGTAAAGCTTGTAGCTGAAGCAGGAATTGGAACTGTAGCCTCTGGAGT
ATCTAAGGCAAATGCAGACGTAATTCAGGTAGATTAAACATCAAAGTTTTAATAAGACAT
TGATTTAGCTCATACAATTATTGTACGTGGATCTGTATCCCATAAGTGGCTATGCTTCTG
TGGTTTAGTGTTATTTTACATTTTTTTTGTACCCTTAACCTTTTGCTGTAAGGTGCTATC
TGCTAAGTTGGCCCTCATGAGGCTGCGTAAACCACCTTTTGCTATTTCATGCTGCAGATA
GTGACATTTGCTGTCTGATGTTCACGATGATAGACCTTCCTATTGTAGTTCATTTTTGTA
TGAAATATGAATTTCTTGCTCCCTGTGGATAGCCAGTTGTTAGACATTGCTATAGTGGCA
CTTGTCCTGTAACAGACAAACATCACTTTTTAGTGAATTATATGCAATAATCTGTCANNN
NNNNNNNNNNNNNNNNNNNNNNNNNNNNNNNNNNNNNNNNNAGAACACACAAATTCCAGA
AATATAGAAATGTTTATTGCACACACAGACACCATAAGGTAAGCAATGACTTATGAAGAC
GCCAATTTAGTTTAATGATGAATTGGATGAGTACACTAAACCTTGAGTCTCTTGAGAGAA
ACCATGCTATGATATCTTATTTAATTTGTTGCGAGTGGTTTCATCCAAAGTCTACTGTAC
TCATGGGTGAACTAGAGGTTTAGTATACATATTTTGGTGATCTAATATGCCATTTTATTA
TACAGATATCAGGTCATGATGGTGGTACTGGAGCTAGCCCAATTAGCTCAATCAAGCATG
CTGGGGGTCCTTGGGAACTTGGTCTTACGGAAACACACCAGGTTTTCTACATTTAATTGC
TTGATATAATCAGTTTTTAATACCAAATCACTTTCTTTGTTCTAAGCCTAGTTAGCTTCC
TTCAGACACTCATACAAAATGGATTGAGAGAGAGGGTGGTACTTAGGGTGGATGGTGGAT
TCAGGAGTGGCCTAGATGTCCTTTTGGCTGCTGCCATGGGTGCTGATGAATATGGCTTTG
GTTCTGTAGCTATGATAGCTACCGGATGTGTCATGGCACGCATTTGCCACACAAACAATT
GCCCAGTTGGAGTTGCTAGTCAGGTAGTAACAGCAGAATTGATATTTTTATTTGTCTTGC
TTGGCTATACCTTACATTCTATGCTCCTGCAGAGAGAGGAGCTACGCGCTAGGTTCCCTG
GTGTTCCTGGTGATCTTGTGAACTACTTCCTCTTTGTTGCAGAGGAGGTATGGCGTCACT
CGCATCACTAGTGCCTTTTCTGTTAACCTCTTTCTGCGCTTGATTTGCAAATTTGGTCAT
GCATATTTTTGGAGTAACCATGACTCATATTTAAAGTACTCATGCTTCTGTTCCAGGTAC
GAGCCACATTAGCCCAGTTGGGTTATGAGAAACTGGATGACATAATTGGGCGGACAGATT
TACTTAAGCCAAAACATATCTCTTTGGTGAAAACGCAGCACATTGATCTTGCATACCTAT
TAATGGTATGGAGTTGATAGCTATTCTCATTTGAGTTATTGGCATTTCATGCCTATCTTG
TTATATTTATCGGCGTTCTCGTTTCAGAATGCTGGATTACCCAAATGGAGCAGCTCTCAG
ATAAGGAGCCAGGACGTCCATTCTAATGGCCCTGTCCTTGATGAGACAATCCTTGCAGAT
CCTGAGGTAATGTTTTTGATAGCTTTTCTTAGTTACATCGATTAGCACCTGCCATGAGCA

GATTTGTGTACACCCAATATTTTTTTTGCTCGGGAACAAAGAACTGTGACGTTGATCAAT
AGATATTGTGGCCTTAAAGTGAAATGCATAGTTGTTAGGAACCAAGCTCTGCTGTATGCA
GCATTACTTAAATTATATAATGACATTTTGCAGGTATCCGATGCAATTGAGAATGAGAAA
GAGGTTTCAAAGACATATCCAATTTATAATGTTGATAGGGCTGTTTGCGGCCGTGTAGCG
GGTGTCATTGCTAAGAAGTACGGAGATACAGGCTTTGCAGGCCAGCTGAACATTACGTAT
GTCAACTACTTTCTTGTCTGCATAATTCTGTGCCCTTTAACTTTTATTATGAGAAGATAG
GAAATAAACAATTTCAAACATTTCAGGTTCACCGGAAGTGCAGGACAGTCCTTTGGTTGT
TTTCTGACACCTGGCATGAATGTTCGCCTAGTAGGAGAGGCCAACGATTATGTGGGGAAG
GTACTAAATGCTCAACTGGACTGAAAATACTGTCTGACCAGCAGCTTTATAATGCCTTCT
ATTTCATTTTCACAGGGTATGGCGGGTGGAGAGCTGGTTGTAGTTCCTGTAGATGATACA
GGATTTGTTCCTGAGGATGCTGCTATAGTTGGGAACACTTGTCTGTATGGAGCTACGGGT
GGTCAAGTATTTGTGAGAGGCAAGACAGGAGAAAGATTTGCAGTTCGGAATTCTCTTGGG
CAAGCAGTGGTTGAGGGCACAGGAGATCATTGCTGCGAGTACATGACCGGTGGATGTGTA
GTTGTACTCGGCAAGTAAGTACACAGTCTGCATTTTCCCCACTTTGTGTATTATTACCCT
TCTTGTCCTGATATATATTTCTCAATCCACAGAGTTGGAAGAAACGTTGCAGCCGGAATG
ACCGGTGGCCTAGCCTACATGTTAGATGAAGATGATACACTAGTCCCGAAGGTATGCAAA
TTTGGAATCTGTAGCTATACCTTCAGCCGCCGTGTAGCATGCATCTAGTGAAAGCCTTGG
ATTCAAATCTCAAGATCATAACGTGCAAGTGTTTTAATAGGTTAACAAGGAGATTGTCAA
GATGCAGAGAGTGAATGCTCCAGCTGGGCAGATGCAGCTCAAGGGTTTGATTGAGGCCTA
TGTTGTAAGTCAAGTTGCACTCTTTGACAGTTTTGTGCCAAAATTGGCCATTCCCCATGT
TCTCATATTTTGGATGTCGCTTTTATAGGAAAAAACGGGCAGCACGAAAGGCGCTAAAAT
TCTGAGCGAATGGGAGGCATATCTGCCACTGTTCTGGCAGTTGGTGCCACCCAGCGAAGA
AGACTCACCAGAAGCTTGTGCTGAGTTTGAGAGAGTACTTGCCAGGCAAAAAACAGCAGT
GCAATCTGCCAAGTGATACTAGCCAAAGCCCAATCGCCAAAATGACCAAAGCTAAATGGA
GGGAAAGCGTTCTTATTGCGATACTGGAAAGTGATCTTCTCAGACCGCGGTCCTTCTGAA
GCACTAACGTCACCAAAGGCTGCAATTTTGTGGAAGCTCTGTCCAAGGCATCTTCTGGAA
ACGAGCTTGCAGACTCCGTCGAGGATTCTTCTGCACGCTCTTGCAGCCTAGGTAACCAAC
CACCCCTGTAAATTGGTTCGCTGACGCTAGGCTGCAAGGCCCGGCCCCTGTTGAACTGCC
GCTTTCTTGTACAGGATTCCTGGCTTCGCTGTGTAATTATATTTTTTGTTCTGGACACAT
TAGTTCTTATATTTATACCCGTCCTGTGGTAGGCATGCGAGTTGCATGTGCTTTAGTTCA
GTATACAGAATAAATAAAGTGTATGATGAAGTAGGACTTCTCAGGTGTCGATAATCGGTG
GAACTTGTTTTTCTTTTTTCAAGTTCTACGGGGCGTCGGCCAACTTAAACGATTTCCATT
ATTAACTCGATGGCGCCAGCGTCACCGAGGTTTGAGGTTTGACAACGTGTGAGAAGAGAG
CCGGAAAGCTCGCGGCTAGGTTTCACCCGACAGCGCGGCGCCCAGCTCGAGGACTAGCCA
TACAACGAGGTGGTTTGTTATCTACGATCGGACGTCCTATATCCACACCAAATCGTCTGG
GTGGCCTGTCCGATTCGTGTGGGTGGCCTGTACGATCAGAAAAACGCCACCTAATCGGAT
CAGCCAAACTCCGCCTAGACGCCTTTGCTGACCGGTACTATCCACACCTTGTCCATTTCC
GGGGCGGATATAGAGACACTTGGGTCAGCCATGTAGGACTGACCGCAAGGGTTGCCTCGT
CCGCATAGATGGCACTGTAGCTCAGCCGCCATTGTTGCTCCGCCGCTGTCCCCGATCCTG
ATCCACAACACCACCCCAATCAGCAATTCCACTGCCCATGCCGTCGACGGACAAGATCAC
CGCCATCAGATGCCACCATCGTATGTTGAAGTCTTTTGTTTAAGAGACTTTGTGATGGAG
AAGCTTCACTCCACAACTACACCGTCAATGGGCATAACTACAATAAGGGGCACCTTACCG
ATGGCATCAATCCTCAATGGACGGCGTTTGTGAAGACTATATCGGATCCCCATGATAGAA
AAATATGTCTCTTTGCACAAGTGCAAGGTGCTAGGAAGGATGTGGAGCGAACATTCTGAG
TGCTCCAAGCTTGTTGGTGTATTGTTTGTGGAGCTGCAATGATGCGGGAAAGCGAAGACA
CTTTGACAACTCATGACATGTTGTGTAATCTTGCACAATATGATTGTTGAGGATGAGGGT
GAAGGGGCAACCCGCATGCATGATTTTGAGAAGCCCAGAGTTCAGGTC

Text S2

>GOGAT Chinese Spring A-genome
AADLNDILAERGACGVGFVANLSNEPSFNVVRDALTALGCMEHRGGCGSDNDSGDGAGLM
SGIPWDLFDDWASKEGLAPFERTHTGVGMVFLPQNENSMAEAKAAVEKVFTDEGLEVLGW
RPVPFNLSVVGPNAKETMPNILQIFVRIAKEDDADDIERELYICRKLIERATKSASWADE
LYFCSLSSRTIIYKGMLRSEVLGQFYLDLKNELYKSPFAIYHRRFSTNTSPRWPLAQPMR
LLGHNGEINTIQGNLNWMRSREATIQSPVWRGRENELRPFGDPKASDSANLDSAAELLLR
SGRSPAEAMMMLVPEAYKNHPTLSVKYPEVIDFYEYYKGQMEAWDGPALLLFSDGRTVGA
CLDRNGLRPARYWKTSDGFVYVASEVGVIPMDESKVVMKGRLGPGMMITVDLETGQVLEN
TEVKKNVASAKPYGTWLQESTRSIKPVNFQSSPVMDNETILRHQQAFGYSSEDVQMVIET
MASQGKEPTFCMGDDIPLAVLSQKPHMLFDYFKQRFAQVTNPAIDPLREGLVMSLEVNIG
KRGNILEVGPENADQVTLSSPVLNEGELESLLKDPKLKPKVLSTYFNIRKGLDGSLENAI
KALCEEADAAVRSGSQLLVLSDRSEALEPTRPAVPILLAVGAIHQHLIQNGLRMSASIVA
DTAQCFSTHQFACLIGYGASAICPYLALETCRQWRLSNKTVNLMRNGKMPTVTIEQAQRN
FIKAVKSGLLKILSKMGISLLSSYCGAQIFEIYGLGQEVVDLAFCGSVSKIGGLTLNELG
RETLSFWVRAFSEDTAKRLENFGFIQSRPGGEFHANNPEMSKLLHKAIREKSDNAYTIYQ
QHLASRPVNVLRDLVELKSERTPIPIGKVEPATSIVERFCTGGMSLGAISRETHEAIAIA
MNRIGGKSNSGEGGEDPIRWSPLTDVVDGYSATLPHLKGLQNGDTATSAIKQVASGRFGV
TPTFLVNAEQIEIKIAQGAKPGEGGQLPGKKVSAYIARLRNSKPGVPLISPPPHHDIYSI
EDLAQLIFDLHQINPKAKVSVKLVAEAGIGTVASGVSKANADVIQISGHDGGTGASPISS
IKHAGGPWELGLTETHQTLIQNGLRERVVLRVDGGFRSGLDVLLAAAMGADEYGFGSVAM
IATGCVMARICHTNNCPVGVASQREELRARFPGVPGDLVNYFLFVAEEVRATLAQLGYEK
LDDIIGRTDLLKPKHISLVKTQHIDLAYLLMNAGLPKWSSSQIRSQDVHSNGPVLDETIL
ADPEVSDAIENEKEVSKTYPIYNVDRAVCGRVAGAIAKKYGDTGFAGQLNITFTGSAGQS
FGCFLTPGMNVRLVGEANDYVGKGMAGGELVVVPVDDTGFVPEDAAIVGNTCLYGATGGQ
VFVRGKTGERFAVRNSLGQAVVEGTGDHCCEYMTGGCVVVLGKVGRNVAAGMTGGLAYML
DEDDTLVPKVNKEIVKMQRVNAPAGQMQLKGLIEAYVEKTGSTKGAKILSEWEAYLPLFW
QLVPPSEEDSPEACAEFERVLARQKTAVQSAK

>GOGAT Chinese Spring B-genome
AADLNDILAERGACGVGFVANLSNEPSFNVVRDALTALGCMEHRGGCGSDNDSGDGAGLM
SGIPWDLFDDWASKEGLAPIERTHTGVGMVFLPQNENSMAEAKAAVEKVFTDEGLEVLGW
RPVPFNLSVVGPNAKETMPNILQIFVRIAKEDDADDIERELYICRKLIERATKSASWADE
LYFCSLSSRTIIYKGMLRSEVLGQFYKDLQNELYKSPFAIYHRRFSTNTSPRWPLAQPMR
LLGHNGEINTIQGNLNWMRSREATIQSPVWRGRENELRPFGDPKASDSANLDSAAELLLR
SGRSPAEAMMMLVPEAYKNHPTLLVKYPEVIDFYEYYKGQMEAWDGPALLLFSDGRTVGA
CLDRNGLRPARYWKTSDGFVYVASEVGVIPMDESKVVMKGRLGPGMMITVDLETGQVLEN
TEVKKNVASAKPYGTWLQESTRSIKPVNFQSSPVMDNETILRHQQAFGYSSEDVQMVIET
MASQGKEPTFCMGDDIPLAVLSQKPHMLFDYFKQRFAQVTNPAIDPLREGLVMSLEVNIG
KRGNILEVGPENADQVTLSSPVLNEGELESLLKDPKLKPKVLSTYFNIRKGLDGSLENAI
KALCEEADAAVRSGSQLLVLSDRSEALEPTRPAVPILLAVGAIHQHLIQNGLRMSASIVA
DTAQCFSTHQFACLIGYGASAICPYLALETCRQWRLSNKTVNLMRNGKMPTVTIEQAQRN
FIKAVKSGLLKILSKMGISLLSSYCGAQIFEIYGLGQEVVDLAFCGSVSKIGGLTLNELG
RETLSFWVRAFSEDTAKRLENFGFIQSRPGGEFHANNPEMSKLLHKAIREKSDNAYTIYQ
QHLASRPVNVLRDLVELKSERTPIPIGKVEPATSIVERFCTGGMSLGAISRETHEAIAIA
MNRIGGKSNSGEGGEDPIRWSPLTDVVDGYSATLPHLKGLQNGDTATSAIKQVASGRFGV
TPTFLVNAEQIEIKIAQGAKPGEGGQLPGKKVSAYIARLRNSKPGVPLISPPPHHDIYSI
EDLAQLIFDLHQINPKAKVSVKLVAEAGIGTVASGVSKANADVIQISGHDGGTGASPISS
IKHAGGPWELGLTETHQTLIQNGLRERVVLRVDGGFRSGLDVLLAAAMGADEYGFGSVAM
IATGCVMARICHTNNCPVGVASQREELRARFPGVPGDLVNYFLFVAEEVRATLAQLGYEK
LDDIIGRTDLLKPKHISLVKTQHIDLAYLLMNAGLPKWSSSQIRSQDVHSNGPVLDETIL
ADPEVSDAIENEKEVSKTYPIYNVDRAVCGRVAGAIAKKYGDTGFAGQLNITFTGSAGQS
FGCFLTPGMNVRLVGEANDYVGKGMAGGELVVVPVDDTGFVPEDAAIVGNTCLYGATGGQ
VFVRGKTGERFAVRNSLGQAVVEGTGDHCCEYMTGGCVVVLGKVGRNVAAGMTGGLAYML
DEDDTLVPKVNKEIVKMQRVNAPAGQMQLKGLIEAYVEKTGSTKGAKILNEWEAYLPLFW
QLVPPSEEDSPEACAEFERVLARQKTAVQSAK

>GOGAT Chinese Spring D-genome
AVSARAVLDLPRRRAPQKPAQEAADLNDILAERGACGVGFVANLSNEPSFNVVRDALTAL

GCMEHRGGCGSDNDSGDGAGLMSGIPWDLFDDWASKEGLAPFERTHTGVGMVFLPQNENS
MAEAKAAVEKVFTDEGLEVLGWRPVPFNLSVVGRNAKETMPNILQIFVRIAKEDDADDIE
RELYICRKLIERATKSASWADELYFCSLSSRTIIYKGMLRSEVLGQFYLDLKNELYKSPF
AIYHRRFSTNTSPRWPLAQPMRLLGHNGEINTIQGNLNWMRSREATIQSPVWRGRENELR
PFGDPKASDSANLDSAAELLLRSGRSPAEAMMMLVPEAYKNHPTLSVKYPEVIDFYEYYK
GQMEAWDGPALLLFSDGRTVGACLDRNGLRPARYWKTSDGFVYVASEVGVIPMDESKVVM
KGRLGPGMMITVDLETGQVLENTEVKKNVASAKPYGTWLQESTRSIKPVNFQSSPVMDNE
TILRHQQAFGYSSEDVQMVIETMASQGKEPTFCMGDDIPLAVLSQKPHMLFDYFKQRFAQ
VTNPAIDPLREGLVMSLEVNIGKRGNILEVGPENADQVTLSSPVLNEGELESLLKDPKLK
PKVLSTYFNIRKGLDGSLENAIKALCEEADAAVRSGSQLLVLSDRSEALEPTRPAVPILL
AVGAIHQHLIQNGLRMSASIVADTAQCFSTHQFACLIGYGASAICPYLALETCRQWRLSN
KTVNLMRNGKMPTVTIEQAQRNFIKAVKSGLLKILSKMGISLLSSYCGAQIFEIYGLGQE
VVDLAFCGSVSKIGGLTLNELGRETLSFWVRAFSEDTAKRLENFGFIQSRPGGEFHANNP
EMSKLLHKAIREKSDNAYTIYQQHLASRPVNVLRDLVELKSERTPIPIGKVEPATSIVER
FCTGGMSLGAISRETHEAIAIAMNRIGGKSNSGEGGEDPIRWSPLTDVVDGYSSTLPHLK
GLQNGDTATSAIKQVASGRFGVTPTFLVNAEQIEIKIAQGAKPGEGGQLPGKKVSAYIAR
LRNSKPGVPLISPPPHHDIYSIEDLAQLIFDLHQINPKAKVSVKLVAEAGIGTVASGVSK
ANADVIQISGHDGGTGASPISSIKHAGGPWELGLTETHQTLIQNGLRERVVLRVDGGFRS
GLDVLLAAAMGADEYGFGSVAMIATGCVMARICHTNNCPVGVASQREELRARFPGVPGDL
VNYFLFVAEEVRATLAQLGYEKLDDIIGRTDLLKPKHISLVKTQHIDLAYLLMNAGLPKW
SSSQIRSQDVHSNGPVLDETILADPEVSDAIENEKEVSKTYPIYNVDRAVCGRVAGVIAK
KYGDTGFAGQLNITFTGSAGQSFGCFLTPGMNVRLVGEANDYVGKGMAGGELVVVPVDDT
GFVPEDAAIVGNTCLYGATGGQVFVRGKTGERFAVRNSLGQAVVEGTGDHCCEYMTGGCV
VVLGKVGRNVAAGMTGGLAYMLDEDDTLVPKVNKEIVKMQRVNAPAGQMQLKGLIEAYVE
KTGSTKGAKILSEWEAYLPLFWQLVPPSEEDSPEACAEFERVLARQKTAVQSAK
